# Supplementary material for: Synthesis and Biological Evaluation of Highly Active 7-Anilino Triazolopyrimidines as Potent Antimicrotubule Agents
Source: Pharmaceutics. 2022 Jun 2;14(6):1191. doi: 10.3390/pharmaceutics14061191 (PMC9230136; doi:10.3390/pharmaceutics14061191)
Supplement: Supplementary file 1 [file pharmaceutics-14-01191-s001.zip › pharmaceutics-1704929-supplementary.pdf]

Supplementary data: Synthesis and Biological Evaluation of Highly Active 7-Anilino Triazolopyrimidines as Potent Antimicrotubule Agents

Paola Oliva, Romeo Romagnoli, Barbara Cacciari, Stefano Manfredini, Chiara Padroni, Andrea Brancale, Salvatore Ferla, Ernest Hamel, Diana Corallo, Sanja Aveic, Noemi Milan, Roberta Bortolozzi, Elena Mariotto, and Giampietro Viola

Synthetic procedure and characterization of

3-aryl-5-amino-1*H*-1,2,4-triazoles (**9a-c**) 4

Synthetic procedure and characterization of

3-anilino-5-amino-1*H*-1,2,4-triazoles (**9d-h**) 4

Synthetic procedure and characterization of

3-benzyl/2'-phentlethyl/3'-phenylpropylamino-5-amino-1*H*-1,2,4-triazoles (**9i-ad**) 5-8

Characterization compounds **10a-ad** 9-12

Characterization compounds **11a-ad** 13-15

^{1}H-NMR and ^{13}C-NMR spectra of compound **7a** 16

^{1}H-NMR and ^{13}C-NMR spectra of compound **7b** 17

^{1}H-NMR and ^{13}C-NMR spectra of compound **7c** 18

^{1}H-NMR and ^{13}C-NMR spectra of compound **7d** 19

^{1}H-NMR and ^{13}C-NMR spectra of compound **7f** 20

^{1}H-NMR and ^{13}C-NMR spectra of compound **7h** 21

^{1}H-NMR and ^{13}C-NMR spectra of compound **7i** 22

^{1}H-NMR and ^{13}C-NMR spectra of compound **7j** 23

^{1}H-NMR and ^{13}C-NMR spectra of compound **7l** 24

^{1}H-NMR and ^{13}C-NMR spectra of compound **7m** 25

^{1}H-NMR and ^{13}C-NMR spectra of compound **7n** 26

^{1}H-NMR and ^{13}C-NMR spectra of compound **7o** 27

^{1}H-NMR and ^{13}C-NMR spectra of compound **7p** 28

^{1}H-NMR and ^{13}C-NMR spectra of compound **7q** 29

^{1}H-NMR and ^{13}C-NMR spectra of compound **7s** 30

^{1}H-NMR and ^{13}C-NMR spectra of compound **7t** 31

^{1}H-NMR and ^{13}C-NMR spectra of compound **7u** 32

^{1}H-NMR and ^{13}C-NMR spectra of compound **7v** 33

^{1}H-NMR and ^{13}C-NMR spectra of compound **7w** 34

^{1}H-NMR and ^{13}C-NMR spectra of compound **7x** 35

^{1}H-NMR and ^{13}C-NMR spectra of compound **7y** 36

^{1}H-NMR and ^{13}C-NMR spectra of compound **7aa** 37

^{1}H-NMR and ^{13}C-NMR spectra of compound **7ab** 38

^{1}H-NMR and ^{13}C-NMR spectra of compound **7ac** 39

^{1}H-NMR and ^{13}C-NMR spectra of compound **7ad** 40

^{1}H-NMR and ^{13}C-NMR spectra of compound **8a** 41

^{1}H-NMR and ^{13}C-NMR spectra of compound **8b** 42

^{1}H-NMR and ^{13}C-NMR spectra of compound **8c** 43

^{1}H-NMR and ^{13}C-NMR spectra of compound **8e** 44

^{1}H-NMR and ^{13}C-NMR spectra of compound **8f** 45

^{1}H-NMR and ^{13}C-NMR spectra of compound **8g** 46

^{1}H-NMR and ^{13}C-NMR spectra of compound **8h** 47

^{1}H-NMR and ^{13}C-NMR spectra of compound **8i** 48

^{1}H-NMR and ^{13}C-NMR spectra of compound **8j** 49

^{1}H-NMR and ^{13}C-NMR spectra of compound **8k** 50

^{1}H-NMR and ^{13}C-NMR spectra of compound **8l** 51

^{1}H-NMR and ^{13}C-NMR spectra of compound **8m** 52

^{1}H-NMR and ^{13}C-NMR spectra of compound **8o** 53

^{1}H-NMR and ^{13}C-NMR spectra of compound **8p** 54

^{1}H-NMR and ^{13}C-NMR spectra of compound **8q** 55

^{1}H-NMR and ^{13}C-NMR spectra of compound **8r** 56

^{1}H-NMR and ^{13}C-NMR spectra of compound **8s** 57

^{1}H-NMR and ^{13}C-NMR spectra of compound **8t** 58

^{1}H-NMR and ^{13}C-NMR spectra of compound **8u** 59

^{1}H-NMR and ^{13}C-NMR spectra of compound **8v** 60

Synthetic procedure for the preparation of 3-aryl-5-amino-1*H*-1,2,4-triazoles (9a-c)

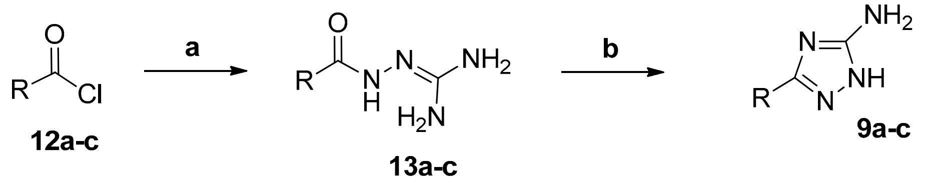

9a, **12a**, **13a**, R=p-F-C_6H_4
9b, **12b**, **13b**, R=p-Cl-C_6H_4
9c, **12c**, **13c**, R=p-OCH_3-C_6H_4

Scheme S1. **Reagents**. **a**: aminoguanidine hydrogen carbonate pyridine, 0 °C for 30 min. then 12 h rt; **b**: water, microwave irradiation, 100 W;

Synthesis of 3-aryl-5-amino-1*H*-1,2,4-triazoles **9a-c** (**9a**, R=*p*-F-C_6H_4; **9b**, R=*p*-Cl-C_6H_4; **9c**, R=*p*-OMe-C_6H_4) was accomplished using a three-step procedure reported in the Scheme S1 according to literature method previously described by us. Briefly, the reaction of aroyl chloride **12a-c** with aminoguanidine hydrogen carbonate using pyridine as solvent furnished the corresponding aroyl amidoguanidines **13a-c**, followed by subsequent ring closure of the intermediate in water under microwave irradiation, to yield 3-substituted-5-amino-1*H*-1,2,4-triazoles **9a-c**.

For the preparation and characterization of compounds **9a-c** and **13a-c** see: R. Romagnoli, F. Prencipe, P. Oliva, S. Baraldi, P.G Baraldi, A. Brancale, S. Ferla, E. Hamel, R. Bortolozzi, G. Viola. 3-Aryl/heteroaryl-5-amino-1-(3',4',5'-trimethoxybenzoyl)-1,2,4-triazoles as antimicrotubule agents. design, synthesis, antiproliferative activity and inhibition of tubulin polymerization. Bioorg. Chem. 80C (2018) 361-374.

Synthetic procedure for the preparation of 3-arylamino-5-amino-1*H*-1,2,4-triazoles (9d-h)

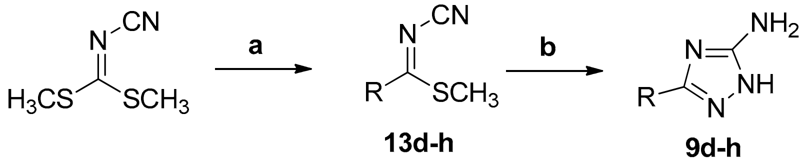

9d, **13d**, R=C_6H_5NH
9e, **13e**, R=4'-F-C_6H_4NH
9f, **13f**, R=4'-Cl-C_4H_4NH
9g, **13g**, R=4'-CH_3-C_4H_4NH
9h, **13h**, R=4'-$OCH_3C_6H_4NH$

Scheme S2. Reagents. **a**: $ArNH_2$, *i*-PrOH, reflux; **b**: $NH_2NH_2.H_2O$, MeOH, rx, 18 h

Compounds **9d-h** were prepared according to the synthetic pathway reported in Scheme S2. The condensation of dimethyl cyanodithioimidocarbonate with the appropriate substituted aniline resulted in the formation of imidates **13d-h**, which were cyclized into the corresponding 5-amino-1*H*-[1,2,4]-triazole derivatives **9d-h** in the presence of hydrazine hydrate in refluxingmethanol .

For the preparation and characterization of compounds **9d-h** and **13d-h** see: R. Romagnoli, P. G. Baraldi, M. Kimatrai Salvador, F. Prencipe, V. Bertolasi, M. Cancellieri, A. Brancale, E. Hamel, I. Castagliuolo, F. Consolaro, E. Porcu, G. Basso, G. Viola. Synthesis, antimitotic and antivascular activity of 1-(3',4',5'-trimethoxybenzoyl)-3-arylamino-5-amino-1,2,4-triazoles. J. Med. Chem. 57 (2014) 6795-6808.

Synthetic procedure for the preparation of 3-substituted-5-amino-1*H*-1,2,4-triazoles (9i-ad)

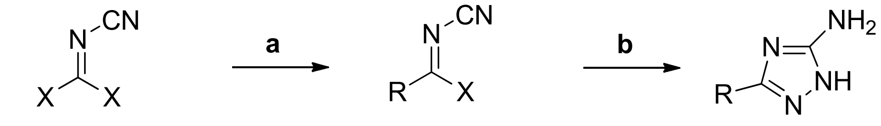

X=OPh or SCH_3 **13i-ad** **9i-ad**

X=OPh for **13j-k**, **13m** and **13o**
X=CH_3 for **13i**, **13l**, **13n** and **13p-ad**

13i, **9i**, R=$C_6H_5CH_2NH$
13j, **9j**, R=4'-pyridinyl-CH_2
13k, **9k**, R=3'-pyridinyl-CH_2
13l, **9l**, R=4'-F-$C_6H_4CH_2NH$
13m, **9m**, R=3'-F-$C_6H_4CH_2NH$
13n, **9n**, R=4'-Cl-$C_6H_4CH_2NH$
13o, **9o**, R=3'-Cl-$C_6H_4CH_2NH$
13p, **9p**, R=4'-CH_3-$C_6H_4CH_2NH$
13q, **9q**, R=3'-CH_3-$C_6H_4CH_2NH$
13r, **9r**, R=2'-CH_3-$C_6H_4CH_2NH$
13s, **9s**, R=4'-OCH_3-$C_6H_4CH_2NH$
13t, **9t**, R=3'-OCH_3-$C_6H_4CH_2NH$
13u, **9u**, R=2'-OCH_3-$C_6H_4CH_2NH$
13v, **9v**, R=3',4'-$(OCH_3)_2$-$C_6H_3CH_2NH$
13w, **9w**, R=4'-CF_3-$C_6H_4CH_2NH$
13x, **9x**, R=4'-CN-$C_6H_4CH_2NH$
13y, **9y**, R=3',4'-(OCH_2O)-$C_6H_3CH_2NH$
13z, **9z**, R=$C_6H_5(CH_2)_2NH$
13aa, **9aa**, R=4'-F-$C_6H_4(CH_2)_2NH$
13ab, **9ab**, R=4'-Cl-$C_6H_4(CH_2)_2NH$
13ac, **9ac**, R=4'-OCH_3-$C_6H_4(CH_2)_2NH$
13ad, **9ad**, R=$C_6H_5(CH_2)_3NH$

Scheme S3. Reagents. a: Appropriate $ArCH_2NH_2$ or $Ar(CH_2)_2NH_2$ or $C_6H_5(CH_2)_3NH_2$, *i*-PrOH, room temperature; **b**: $NH_2NH_2.H_2O$, MeOH, reflux, 18 h

Synthesis of compounds **9i-ad** was accomplished using a two-step protocol described in Scheme S3. The condensation of dimethyl cyanodithioimidocarbonate or diphenoxymethylidenecyanamide with the appropriate benzyl/phenylethylamine or 3-phenylpropylamine resulted in the formation of imidates **13i-ad**, which were cyclized into the corresponding 3-substituted-5-amino-1*H*-1,2,4-triazoles **9i-ad** with hydrazine hydrate in refluxing methanol.

General procedure (a) for the preparation of compounds 13i-ad. To a solution of dimethyl cyanodithioimidocarbonate (292 mg, 2 mmol) or diphenoxymethylidenecyanamide (500 mg, 2 mmol) in isopropanol (10 mL) was added the appropriate amine (2 mmol, 1 equiv.) and the mixture was stirred at room temperature for 24 h. After this time, the mixture was filtered, the solid residue washed with ethyl ether to furnish the final compound **13i-ad** that was used for the next reaction without any purification.

Compounds **13i**, **13l**, **13n**, **13p-q**, **13s-v**, **13z**, **13y** and **13ab-ad** (X=CH_3) have been already described and for their characterization see: P. Oliva, V. Onnis, E. Balboni, E. Hamel, F. Estévez-Sarmiento, J. Quintana, F. Estévez, A. Brancale, S. Ferla, S. Manfredini, R. Romagnoli. Synthesis and biological evaluation of 2-substituted benzyl/phenylethylamino-4-amino-5-aroyl thiazoles as apoptosis inducing anticancer agents Molecules, 25 (2020) 2177.

*(Z)- phenyl N'-cyano-N-(pyridin-4-ylmethyl)carbamimidate (**13j**).* Following general procedure (a) reported in Scheme S3, by the condensation of diphenoxymethylidenecyanamide and pyridin-4-ylmethanamine, compound **13j** was obtained as a yellow solid, yield 67%, mp 110-112 °C. ^{1}H-NMR (DMSO-d_6) δ: 4.60 (bs, 2H), 7.02-7.10 (m, 2H), 7.26-7.45 (m, 6H), 7.51 (d, *J*=6.0 Hz, 1H), 8.57 (d, *J*=8.0 Hz, 1H). MS (ESI): $[M+1]^+$=253.1.

*(Z)- phenyl N'-cyano-N-(pyridin-3-ylmethyl)carbamimidate (**13k**).* Following general procedure (a) reported in Scheme S3, by the condensation of diphenoxymethylidenecyanamide and pyridin-3-ylmethanamine, compound **13k** was obtained as a white solid, yield 56%, mp 167-169 °C. ^{1}H-NMR (DMSO-d_6) δ: 4.60 (bs, 2H), 7.08 (d, *J*=7.0 Hz, 1H), 7.24-7.57 (m, 5H), 7.69-7.86 (m, 1H), 8.62 (d, *J*=8.0 Hz, 1H). MS (ESI): $[M+1]^+$=253.1.

*(Z)-phenyl N'-cyano-N-(3-fluorobenzyl)carbamimidate (**13m**).* Following general procedure (a) reported in Scheme S3, by the condensation of diphenoxymethylidenecyanamide and 3-fluorobenzylamine, compound **13m** was obtained as a white solid, yield 73%, mp 215-217 °C. ^{1}H-NMR (DMSO-d_6) δ: 4.54 (bs, 2H), 7.02-7.56 (m, 9H), 8.92 (bs, 1H). MS (ESI): $[M+1]^+$=270.1.

*(Z)-phenyl N'-cyano-N-(3-chlorobenzyl)carbamimidate (**13o**).* Following general procedure (a) reported in Scheme S3, by the condensation of diphenoxymethylidenecyanamide and 3-chlorobenzylamine, compound **13o** was obtained as a white solid, yield 68%, mp 201-203 °C. ^{1}H-NMR (DMSO-d_6) δ: 4.44 (bs, 2H), 7.06 (d, *J*=8.0 Hz, 1H), 7.33-7.55 (m, 8H), 8.54 (bs, 1H). MS (ESI): $[M+1]^+$=288.1.

*(Z)-methyl N'-cyano-N-(2-methylbenzyl)carbamimidothioate (**13r**).* Following general procedure (a) reported in Scheme S3, by the condensation of dimethyl cyanodithioimidocarbonate and 2-methylbenzylamine, compound **13r** was obtained as a white solid, yield 92%, mp 185-188 °C. ^{1}H-NMR ($CDCl_3$) δ: 2.30 (s, 3H), 2.64 (s, 3H), 4.42 (d, *J*=6.2 Hz, 2H), 6.90-6.94 (m, 3H), 7.36 (t, *J*=8.2 Hz, 1H). MS (ESI): $[M+1]^+$=220.2.

*(Z)-methyl N'-cyano-N-(4-trifluoromethylbenzyl)carbamimidothioate (**13w**).* Following general (a) procedure reported in Scheme S3, by the condensation of dimethyl cyanodithioimidocarbonate and 4-trifluoromethylbenzylamine, compound **13w** was obtained as a yellow solid, yield >95%, mp 173-175 °C. ^{1}H-NMR ($CDCl_3$) δ: 2.52 (s, 3H), 4.58 (d, J=5.6 Hz, 2H), 7.40 (d, J=8.2 Hz, 2H), 7.61 (d, *J*=8.2 Hz, 2H). MS (ESI): $[M+1]^+$=274.3.

*(Z)-methyl N'-cyano-N-(4-cyanobenzyl)carbamimidothioate (**13x**).* Following general procedure (a) reported in Scheme S3, by the condensation of dimethyl cyanodithioimidocarbonate and 4-cyanobenzylamine, compound **13x** was obtained as a yellow solid, yield: 76%, mp 225-227 °C. ^{1}H-NMR (DMSO-d_6) δ: 2.62 (s, 3H), 4.54 (bs, 2H), 7.44 (d, *J*=8.2 Hz, 2H), 7.78 (d, *J*=8.2 Hz, 2H), 8.86 (bs, 1H). MS (ESI): $[M+1]^+$=231.3.

*(Z)-methyl N'-cyano-N-(4-fluorophenethyl)carbamimidothioate (**13aa**).* Following general (a) procedure reported in Scheme S3, by the condensation of dimethyl cyanodithioimidocarbonate and 4-fluorophenylethylamine, compound **13aa** was obtained as a white solid, yield 90%, mp 173-175 °C. ^{1}H-NMR ($CDCl_3$) δ: 2.46 (s, 3H), 2.91 (t, *J*=7.2 Hz, 2H), 3.60 (bs, 2H), 7.07 (t, *J*=8.4 Hz, 2H), 7.20-7.22 (m, 2H). MS (ESI): $[M+1]^+$=238.3.

General procedure (b) for the preparation of 3-substituted-5-amino-1*H*-1,2,4-triazoles (9i-ad). To a stirred suspension of compound **13i-ad** (2 mmol) in methanol (10 mL) was added hydrazine monohydrate (0.2 mL, 4 mmol, 2 equiv.) and the mixture was heated under reflux for 18 h. After this time, the volatiles were removed and the residue suspended with ethyl ether (10 mL) was sonicated for 10 min. The resultant solid was collected by filtration and then used for the next reaction without any purification.

Compounds **9i**, **9n**, **9s**, **9v** and **9ad** have already been reported and for their characterization see: V.M. Chernyshev, V.A. Rakitov, A.V. Astakhov, A.N. Sokolov, N.D. Zemlyakov, V.A. Taranushich. Regioselective synthesis of alkyl derivatives of 3,5-diamino-1,2,4-triazole. Russ. J. Appl. Chem. 79. (2006), 624-630.

Compound **9z** was previously described and for its characterization see: A.V. Dolzhenko, A.V. Dolzhenko, W.K. Chui. Synthesis of 5,7-diamino[1,2,4]triazolo[1,2-a][1,3,5]triazines via annulation of 1,3,5-triazine ring onto 3(5)-amino-1,2,4-triazoles. Heterocycles 71 (2007) 429-436.

N^3-(pyridin-4-ylmethyl)-1H-1,2,4-triazole-3,5-diamine (**9j**). Following general procedure (b) reported in the Scheme S3, compound **9j** was obtained as a white solid. Yield 63%; mp 210-212 °C. ^{1}H-NMR (d_6-DMSO) δ: 4.24 (d, J=6.38 Hz, 2H), 5.39 (br. s., 2H), 6.19 (br. s., 1H), 7.25-7.32, (m, 2H), 8.42 - 8.49 (m, 2H). MS (ESI): $[M+1]^+$=191.08.

N^3-(pyridin-3-ylmethyl)-1H-1,2,4-triazole-3,5-diamine (**9k**). Following general procedure (b) reported in the Scheme S3, compound **9k** was obtained as a white solid. Yield 67%; mp 198-200 °C. ^{1}H-NMR (d_6-DMSO) δ: 4.23 (d, J=6.2 Hz, 2H), 5.43-5.91 (m, 2H) 7.31 (dd, J=7.70 and 4.84 Hz, 1H) 7.71 (dt, J=7.87 and 1.90 Hz, 1H) 8.38-8.45 (m. 1H) 8.52 (d, J=1.76 Hz. 1H), 10.64 (br. s., 1H). MS (ESI): $[M+1]^+$=191.2.

N^3-(4-fluorobenzyl)-1H-1,2,4-triazole-3,5-diamine (**9l**). Following general procedure (b) reported in the Scheme S3, compound **9l** was obtained as a white solid. Yield 87%; mp 178-180 °C. ^{1}H-NMR (d_6-DMSO) δ: 4.20 (d, J=6.4 Hz, 2H), 5.43 (bs, 2H), 5.77-6.11 (m, 1H), 7.06-7.14 (m, 2H), 7.30-7.37 (m, 2H), 10.35 - 10.45 (bs, 1H). MS (ESI): $[M+1]^+$=208.3.

N^3-(3-fluorobenzyl)-1H-1,2,4-triazole-3,5-diamine (**9m**). Following general procedure (b) reported in the Scheme S3, compound **9m** was obtained as a white solid. Yield 53%; mp 184-186 °C. ^{1}H-NMR (d_6-DMSO) δ: 4.23 (d, J=6.4 Hz, 2H), 5.48 (bs, 1H), 7.01 (dt, J=8.6 and 2.2 Hz, 1H), 7.08-7.17 (m, 2H), 7.28-7.37 (m, 1H), 10.6 (bs, 1H). MS (ESI): $[M+1]^+$=208.2.

N^3-(3-chlorophenethyl)-1H-1,2,4-triazole-3,5-diamine (**9o**). Following general procedure (b) reported in the Scheme S3 compound **9o** was obtained as a white solid. Yield 93%; mp 186-188 °C. ^{1}H-NMR (d_6-DMSO) δ: 4.22 (d, J=6.2 Hz, 2H), 5.27-5.68 (m, 2H), 7.22-7.28 (m, 2H), 7.31 (d, J=7.5 Hz, 1H), 7.36 (s, 1H), 10.4 (bs, 1H). MS (ESI): $[M+1]^+$=224.3.

N^3-(4-methylbenzyl)-1H-1,2,4-triazole-3,5-diamine (**9p**). Following general procedure (b) reported in the Scheme S3, compound **9p** was obtained as a white solid. Yield 87%; mp 188-190 °C. ^{1}H-NMR (d_6-DMSO) δ: 2.25 (s, 3H), 4.14 (d, J=6.2 Hz, 2H), 5.27 (bs, 2H), 5.85 (t, J=6.2 Hz, 1H), 7.05 (d, J=8.0 Hz, 2H), 7.16 (d, J=8.0 Hz, 2H), 10.7 (bs, 1H). MS (ESI): $[M+1]^+$=203.9.

N^3-(3-methylbenzyl)-1H-1,2,4-triazole-3,5-diamine (**9q**). Following general procedure (b) reported in the Scheme S3, compound **9q** was obtained as a white solid. Yield >95%; mp 173-175 °C. ^{1}H-NMR (d_6-DMSO) δ: 2.23 (s, 3H), 4.17 (d, J=6.4 Hz, 2H), 4.56 (bs, 1H), 5.62 (bs, 2H), 6.96 (t, J=6.4 Hz, 1H), 7.00 (dd, J=8.4 and 2.6 Hz, 1H), 7.08 (dd, J=8.4 and 2.6 Hz, 1H), 7.14 (s, 1H), 7.20 (t, J=8.4 Hz, 1H), 10.6 (bs, 1H). MS (ESI): $[M+1]^+$=204.2.

N^3-(2-methylbenzyl)-1H-1,2,4-triazole-3,5-diamine (**9r**). Following general procedure (b) reported in the Scheme S3, compound **9r** was obtained as a white solid. Yield 92%; mp 162-164 °C. ^{1}H-NMR (d_6-DMSO) δ: 2.28 (s, 3H), 4.18 (d, J=6.4 Hz, 2H), 5.62 (bs, 2H), 6.96 (t, J=6.4 Hz, 1H), 7.10-7.15 (m, 2H), 7.20-7.24 (m, 2H), 10.6 (bs, 1H). MS (ESI): $[M+1]^+$=204.2.

N^3-(3-methoxybenzyl)-1H-1,2,4-triazole-3,5-diamine (**9t**). Following general procedure (b) reported in the Scheme S3, compound **9t** was obtained as a white solid. Yield 88%; mp 178-180 °C. ^{1}H-NMR (CD_3OD) δ: 3.82 (s, 3H), 4.34 (s, 2H), 6.80 (dd, J=7.4 and 2.4 Hz, 1H), 6.90-6.95 (m, 2H), 7.22 (t, J=7.4 Hz, 1H). MS (ESI): $[M+1]^+$=220.3.

N^3-(2-methoxybenzyl)-1H-1,2,4-triazole-3,5-diamine (**9u**). Following general procedure (b) reported in the Scheme S3, compound **9u** was obtained as a white solid. Yield 89%; mp 201-203 °C. ^{1}H-NMR (CD_3OD) δ: 3.80 (s, 3H), 4.32 (s, 2H), 6.86 (t, J=8.2 Hz, 1H), 6.94 (d, J=8.2 Hz, 1H), 7.18-7.22 (m, 2H). MS (ESI): $[M+1]^+$=220.2.

N^3-(4-(trifluoromethyl)benzyl)-1H-1,2,4-triazole-3,5-diamine (**9w**). Following general procedure (b) reported in the Scheme S3, compound **9w** was obtained as a yellow solid. Yield 86%; mp 173-175 °C. ^{1}H-NMR (d_6-DMSO) δ: 4.28 (d, J=6.3 Hz, 2H), 5.64 (bs, 2H), 6.22 (bs, 1H), 7.49 (d, J=8.1 Hz, 2H), 7.63 (d, J=8.1 Hz, 2H), 10.7 (bs, 1H). MS (ESI): $[M+1]^+$=258.3.

4-(((5-amino-1H-1,2,4-triazol-3-yl)amino)methyl)benzonitrile (**9x**). Following general procedure (b) reported in the Scheme S3, compound **9x** was obtained as a white solid. Yield 74%; mp 258-260 °C. ^{1}H-NMR (d_6-DMSO) δ: 4.27 (d, J=6.0 Hz, 2H), 5.62 (bs, 2H), 6.03 (bs, 1H), 7.46 (d, J=8.1 Hz, 2H), 7.74 (d, J=8.1 Hz, 2H), 10.8 (bs, 1H). MS (ESI): $[M+1]^+$=215.3.

N^3-(benzo[d][1,3]dioxol-5-ylmethyl)-1H-1,2,4-triazole-3,5-diamine (**9y**). Following general procedure (b) reported in the Scheme S3, compound **9y** was obtained as a white solid. Yield 93%; mp 204-206 °C. ^{1}H-NMR (d_6-DMSO) δ: 4.12 (d, J=6.4 Hz, 2H), 4,24 (bs, 1H), 5.38 (bs, 2H), 5.97 (s, 2H), 6.74 (dd, J=8.2 and 2.2 Hz, 1H), 6.80 (d, J=8.2 Hz, 1H), 6.88 (d, J=2.2 Hz, 1H), 10.8 (bs, 1H). MS (ESI): $[M+1]^+$=234.2.

N^3-(4-Fluorophenethyl)-1H-1,2,4-triazole-3,5-diamine (**9aa**). Following general procedure (b) reported in the Scheme S3, compound **9aa** was obtained as a white solid. Yield >95%; mp 210-212 °C. ^{1}H-NMR (d_6-DMSO) δ: 2.74 (t, J=7.5 Hz, 2H), 3.16 (m, 2H), 3.31 (m, 2H), 5.22 (bs, 2H), 5.80 (bs, 1H), 7.04 (t, J=8.4 Hz, 2H), 7.20-7.22 (m, 2H), 10.6 (bs, 1H). MS (ESI): $[M+1]^+$=222.2.

N^3-(4-chlorophenethyl)-1H-1,2,4-triazole-3,5-diamine (**9ab**). Following general procedure (b) reported in the Scheme S3, compound **9ab** was obtained as a white solid. Yield 95%; mp 202-204 °C. ^{1}H-NMR (d_6-DMSO) δ: 2.70 (t, J=7.2 Hz, 2H), 3.16 (m, 2H), 3.36 (t, J=6.4 Hz, 2H), 5.24 (bs, 2H), 5.84 (bs, 1H), 6.83 (d, J=8.4 Hz, 2H), 7.11 (d, J=8.4 Hz, 2H), 10.6 (bs, 1H). MS (ESI): $[M+1]^+$=224.3.

N^3-(4-methoxyphenethyl)-1H-1,2,4-triazole-3,5-diamine (**9ac**). Following general procedure (b) reported in the Scheme S3, compound **9ac** was obtained as a pink solid. Yield >95%; mp 252-254 °C. ^{1}H-NMR (d_6-DMSO) δ: 2.71 (t, J=7.2 Hz, 2H), 3.16 (m, 2H), 3.32 (t, J=6.4 Hz, 2H), 3.71 (s, 3H), 5.24 (bs, 2H), 5.84 (bs, 1H), 6.85 (d, J=8.4 Hz, 2H), 7.13 (d, J=8.4 Hz, 2H), 10.6 (bs, 1H). MS (ESI): $[M+1]^+$=234.3.

Characterization of compounds 10a-ad

*2-(4-Fluorophenyl)-5-methyl-[1,2,4]triazolo[1,5-a]pyrimidin-7-ol (**10a**).* Following general procedure A, starting from 3-(4-fluorophenyl)-1*H*-1,2,4-triazol-5-amine (**9a**), compound **10a** was obtained as a white solid. Yield: 74%, mp 234-236 °C. ^{1}H-NMR (DMSO-d_6) δ: 2.31 (s, 3H), 5.83 (s, 1H), 7.32 (t, J=8.8 Hz, 2H), 8.10-8.14 (m, 2H), 13.2 (bs, 1H). MS (ESI): $[M+1]^+$=245.3.

*2-(4-Chlorophenyl)-5-methyl-[1,2,4]triazolo[1,5-a]pyrimidin-7-ol (**10b**).* Following general procedure A, starting from 3-(4-chlorophenyl)-1*H*-1,2,4-triazol-5-amine (**9b**), compound **10b** was obtained as a white solid. Yield: 90%, mp 270-272 °C. ^{1}H-NMR (DMSO-d_6) δ: 2.32 (s, 3H), 5.84 (s, 1H), 7.56 (dd, J=8.8 and 2.0 Hz, 2H), 8,07 (dd, J=8.8 and 2.0 Hz, 2H), 13.3 (bs, 1H). MS (ESI): $[M+1]^+$=261.2.

*5-Methyl-2-(p-tolyl)-[1,2,4]triazolo[1,5-a]pyrimidin-7-ol (**10c**).* Following general procedure A, starting from 3-(*p*-tolyl)-1*H*-1,2,4-triazol-5-amine (**9c**), compound **10c** was obtained as a pink solid. Yield: 68%, mp 252-254 °C. ^{1}H-NMR (DMSO-d_6) δ: 2.33 (s, 3H), 2.38 (s, 3H), 5.84 (s, 1H), 7.33 (d, J=7.6 Hz, 2H), 7.99 (d, J=7.6 Hz, 2H), 13.4 (bs, 1H). MS (ESI): $[M+1]^+$=241.4.

*5-Methyl-2-(phenylamino)-[1,2,4]triazolo[1,5-a]pyrimidin-7-ol (**10d**).* Following general procedure A, starting from *N^3*-phenyl-4*H*-1,2,4-triazole-3,5-diamine **9d**, compound **10d** was obtained as a white solid. Yield: 88%, mp 250-252 °C. ^{1}H-NMR (DMSO-d_6) δ: 2.26 (s, 3H), 5.73 (s, 1H), 6.84 (t, J=7.6 Hz, 1H), 7.26 (t, J=7.6 Hz, 2H), 7.62 (d, J=6.8 Hz, 2H), 9.50 (s, 1H), 12.8 (bs, 1H). MS (ESI): $[M+1]^+$=242.3.

*2-((4-Fluorophenyl)amino)-5-methyl-[1,2,4]triazolo[1,5-a]pyrimidin-7-ol (**10e**).* Following general procedure A, starting from N^3-(4-fluorophenyl)-1*H*-1,2,4-triazole-3,5-diamine (**9e**), compound **10e** was obtained as a white solid. Yield: 75%, mp 252-254 °C. ^{1}H-NMR (DMSO-d_6) δ: 2.26 (s, 3H), 5.72 (s, 1H), 7.11 (t, J=9.2 Hz, 2H), 7.62-7.65 (m, 2H), 9.54 (s, 1H), 11.8 (bs, 1H). MS (ESI): $[M+1]^+$=260.3.

*2-((4-Chlorophenyl)amino)-5-methyl-[1,2,4]triazolo[1,5-a]pyrimidin-7-ol (**10f**).* Following general procedure A, starting from *N^3*-(4-chlorophenyl)-1*H*-1,2,4-triazole-3,5-diamine (**9f**), compound **10f** was obtained as a white solid. Yield: 82%, mp 260-262 °C. ^{1}H-NMR (DMSO-d_6) δ: 2.26 (s, 3H), 5.75 (s, 1H), 7.31 (d, J=8.4 Hz, 2H), 7.64 (d, J=8.4 Hz, 2H), 9.71 (s, 1H), 12.9 (bs, 1H). MS (ESI): $[M+1]^+$=276.3.

*5-Methyl-2-(p-tolylamino)-[1,2,4]triazolo[1,5-a]pyrimidin-7-ol (**10g**).* Following general procedure A, starting from *N^3*-(*p*-tolyl)-1*H*-1,2,4-triazole-3,5-diamine (**9g**), compound **10g** was obtained as a white solid. Yield: 87%, mp 250-252 °C. ^{1}H-NMR (DMSO-d_6) δ: 2.22 (s, 3H), 2.26 (s, 3H), 5.71 (s, 1H), 7.05 (d, J=8.4 Hz, 2H), 7.51 (d, J=8.4 Hz, 2H), 9.38 (s, 1H), 12.8 (bs, 1H). MS (ESI): $[M+1]^+$=256.3.

*2-((4-Methoxyphenyl)amino)-5-methyl-[1,2,4]triazolo[1,5-a]pyrimidin-7-ol (**10h**).* Following general procedure A, starting from N^3-(4-methoxyphenyl)-1*H*-1,2,4-triazole-3,5-diamine (**9h**), compound **10h** was obtained as a pink solid. Yield: 76%, mp 234-236 °C. ^{1}H-NMR (DMSO-d_6) δ:

2.26 (s, 3H), 3.70 (s, 3H), 5.71 (s, 1H), 6.85 (d, J=9.2 Hz, 2H), 7.53 (d, J=9.2 Hz, 2H), 9.27 (s, 1H), 12.7 (bs, 1H). MS (ESI): $[M+1]^+$=272.3.

*2-(Benzylamino)-5-methyl-[1,2,4]triazolo[1,5-a]pyrimidin-7-ol (**10i**)*. Following general procedure A, starting from N^3-benzyl-1*H*-1,2,4-triazole-3,5-diamine (**9i**), compound **10i** was obtained as a white solid. Yield: 72%, mp 232-234 °C. ^{1}H-NMR (DMSO-d_6) δ: 2.22 (s, 3H), 4.37 (d, J=6.4 Hz, 2H), 5.64 (s, 1H), 7.13 (t, J=6.4 Hz, 1H), 7.20-7.21 (m, 1H), 7.30-7.34 (m, 4H), 12.8 (bs, 1H). MS (ESI): $[M+1]^+$=256.4.

*5-Methyl-2-((pyridin-4-ylmethyl)amino)-[1,2,4]triazolo[1,5-a]pyrimidin-7-ol (**10j**)*. Following general procedure A, starting from N^3-(pyridin-4-ylmethyl)-1*H*-1,2,4-triazole-3,5-diamine (**9j**), compound **10j** was obtained as a pink solid. Yield: 72%, mp 263-265 °C. ^{1}H-NMR (DMSO-d_6) δ: 2.22 (s, 3H), 4.41 (d, J=6.4 Hz, 2H), 5.64 (s, 1H), 7.22 (s, 1H), 7.32 (d, J=6.2 Hz, 2H), 8.45-8.51 (m, 2H), 12.7 (bs, 1H). MS (ESI): $[M+1]^+$=257.2.

*5-Methyl-2-((pyridin-3-ylmethyl)amino)-[1,2,4]triazolo[1,5-a]pyrimidin-7-ol (**10k**)*. Following general procedure A, starting from N^3-(pyridin-3-ylmethyl)-1*H*-1,2,4-triazole-3,5-diamine (**9k**), compound **10k** was obtained as a pink solid. Yield: 76%, mp 252-254 °C. ^{1}H-NMR (DMSO-d_6) δ: 2.64 (s, 3H), 4.41 (d, J=6.2 Hz, 2H), 5.64 (s, 1H), 7.17 (t, J=6.2 Hz, 1H), 7.34 (dd, J=7.2 and 4.8 Hz, 1H), 7.74 (d, J=7.70 Hz, 1 H), 8.44 (dd, J=4.8 and 1.6 Hz, 1H), 8.56 (d, J=1.6 Hz, 1H), 12.8 (bs, 1H). MS (ESI): $[M+1]^+$=257.1.

*2-((4-Fluorobenzyl)amino)-5-methyl-[1,2,4]triazolo[1,5-a]pyrimidin-7-ol (**10l**)*. Following general procedure A, starting from N^3-(4-fluorobenzyl)-1*H*-1,2,4-triazole-3,5-diamine (**9l**), compound **10l** was obtained as a pink solid. Yield: 81%, mp 240-242 °C. ^{1}H-NMR (DMSO-d_6) δ: 2.22 (s, 3H), 4.36 (d, J=6.4 Hz, 2H), 5.63 (s, 1H), 7.08-7.13 (m, 3H), 7.34-7.41 (m, 2H), 12.7 (bs, 1H). MS (ESI): $[M+1]^+$=274.1.

*2-((3-Fluorobenzyl)amino)-5-methyl-[1,2,4]triazolo[1,5-a]pyrimidin-7-ol (**10m**)*. Following general procedure A, starting from N^3-(3-fluorobenzyl)-1*H*-1,2,4-triazole-3,5-diamine (**9m**), compound **10m** was obtained as a pink solid. Yield: 61%, mp 250-252 °C. ^{1}H-NMR (DMSO-d_6) δ: 2.22 (s, 3H), 4.40 (d, J=6.4 Hz, 2H), 5.64 (s, 1H), 7.04 (dt, J=8.5 and 2.9 Hz, 1H), 7.11-7.21 (m, 3H), 7.32-7.39 (m, 1H), 12.7 (bs, 1H). MS (ESI): $[M+1]^+$=274.1.

*2-((4-Chlorobenzyl)amino)-5-methyl-[1,2,4]triazolo[1,5-a]pyrimidin-7-ol (**10n**)*. Following general procedure A, starting from N^3-(4-chlorobenzyl)-1*H*-1,2,4-triazole-3,5-diamine (**9n**), compound **10n** was obtained as a pink solid. Yield: 82%, mp 258-260 °C. ^{1}H-NMR (DMSO-d_6) δ: 2.20 (s, 3H), 4.34 (d, J=6.4 Hz, 2H), 5.61 (s, 1H), 7.11 (t, J=6.4 Hz, 1H), 7.34 (s, 4H), 12.7 (bs, 1H). MS (ESI): $[M+1]^+$=290.7.

*2-((3-Chlorobenzyl)amino)-5-methyl-[1,2,4]triazolo[1,5-a]pyrimidin-7-ol (**10o**)*. Following general procedure A, starting from N^3-(3-chlorobenzyl)-1*H*-1,2,4-triazole-3,5-diamine (**9o**), compound **10o** was obtained as a pink solid. Yield: 83%, mp 234-236 °C. ^{1}H-NMR (DMSO-d_6) δ: 2.32 (s, 3H), 4.39 (d, J=6.4 Hz, 2H), 5.64 (s, 1H), 7.17 (s, 1H), 7.28-7.36 (m, 4H), 12.7 (bs, 1H). MS (ESI): $[M+1]^+$=290.1.

*5-Methyl-2-((4-methylbenzyl)amino)-[1,2,4]triazolo[1,5-a]pyrimidin-7-ol (**10p**)*. Following general procedure A, starting from N^3-(4-methylbenzyl)-1*H*-1,2,4-triazole-3,5-diamine (**9p**), compound **10p** was obtained as a pink solid. Yield: 68%, mp 262-264 °C. ^{1}H-NMR (DMSO-d_6) δ: 2.22 (s, 3H), 2.26 (s, 3H), 4.32 (d, J=6.4 Hz, 2H), 5.62 (s, 1H), 7.02 (t, J=6.4 Hz, 1H), 7.11 (d, J=8.0 Hz, 2H), 7.20 (d, J=8.0 Hz, 2H), 13.2 (bs, 1H). MS (ESI): $[M+1]^+$=270.3.

*5-Methyl-2-((3-methylbenzyl)amino)-[1,2,4]triazolo[1,5-a]pyrimidin-7-ol (**10q**).* Following general procedure A, starting from N^3-(3-methylbenzyl)-1*H*-1,2,4-triazole-3,5-diamine (**9q**), compound **10q** was obtained as a pink solid. Yield: 65%, mp 180-182 °C. ^{1}H-NMR (DMSO-d_6) δ: 2.22 (s, 3H), 2.46 (s, 3H), 4.32 (d, J=6.4 Hz, 2H), 5.61 (s, 1H), 7.01 (t, J=6.4 Hz, 1H), 7.02-7.12 (m, 4H), 12.6 (bs, 1H). MS (ESI): $[M+1]^+$=270.3.

*5-Methyl-2-((2-methylbenzyl)amino)-[1,2,4]triazolo[1,5-a]pyrimidin-7-ol (**10r**).* Following general procedure A, starting from N^3-(2-methylbenzyl)-1*H*-1,2,4-triazole-3,5-diamine (**9r**), compound **10r** was obtained as a pink solid. Yield: 64%, mp 236-238 °C. ^{1}H-NMR (DMSO-d_6) δ: 2.20 (s, 3H), 2.29 (s, 3H), 4.32 (d, J=6.4 Hz, 2H), 5.61 (s, 1H), 7.00 (t, J=6.4 Hz, 1H), 7.08-7.14 (m, 3H), 7.32 (t, J=8.4 Hz, 1H), 12.6 (bs, 1H). MS (ESI): $[M+1]^+$=270.4.

*2-((4-Methoxybenzyl)amino)-5-methyl-[1,2,4]triazolo[1,5-a]pyrimidin-7-ol (**10s**).* Following general procedure A, starting from N^3-(4-methoxybenzyl)-1*H*-1,2,4-triazole-3,5-diamine (**9s**), compound **9s** was obtained as a white solid. Yield: 70%, mp 270-272 °C. ^{1}H-NMR (DMSO-d_6) δ: 2.22 (s, 3H), 3.71 (s, 3H), 4.29 (d, J=6.4 Hz, 2H), 5.63 (s, 1H), 6.85 (d, J=8.4 Hz, 2H), 7.03 (t, J=6.4 Hz, 1H), 7.24 (d, J=8.4 Hz, 2H), 12.8 (bs, 1H). MS (ESI): $[M+1]^+$=286.4.

*2-((3-Methoxybenzyl)amino)-5-methyl-[1,2,4]triazolo[1,5-a]pyrimidin-7-ol (**10t**).* Following general procedure A, starting from N^3-(3-methoxybenzyl)-1*H*-1,2,4-triazole-3,5-diamine (**9t**), compound **10t** was obtained as a pink solid. Yield: 84%, mp 260-262 °C. ^{1}H-NMR (DMSO-d_6) δ: 2.21 (s, 3H), 3.71 (s, 3H), 4.33 (d, J=6.4 Hz, 2H), 5.62 (s, 1H), 6.77 (dd, J=8.4 and 2.0 Hz, 1H), 6.88-6.91 (m, 2H), 7.09 (t, J=8.4 Hz, 1H), 7.20 (t, J=8.4 Hz, 1H), 12.7 (bs, 1H). MS (ESI): $[M+1]^+$=286.4.

*2-((2-Methoxybenzyl)amino)-5-methyl-[1,2,4]triazolo[1,5-a]pyrimidin-7-ol (**10u**).* Following general procedure A, starting from N^3-(2-methoxybenzyl)-1*H*-1,2,4-triazole-3,5-diamine (**9u**), compound **10u** was obtained as a pink solid. Yield: 75%, mp 148-150 °C. ^{1}H-NMR (DMSO-d_6) δ: 2.20 (s, 3H), 3.80 (s, 3H), 4.33 (d, J=6.0 Hz, 2H), 5.61 (s, 1H), 6.85-6.90 (m, 2H), 6.94 (d, J=7.6 Hz, 1H), 7.18-7.21 (m, 2H), 12.6 (bs, 1H). MS (ESI): $[M+1]^+$=286.4.

*2-((3,4-dimethoxybenzyl)amino)-5-methyl-[1,2,4]triazolo[1,5-a]pyrimidin-7-ol (**10v**).* Following general procedure A, starting from N^3-(3,4-dimethoxybenzyl)-1*H*-1,2,4-triazole-3,5-diamine (**9v**), compound **10v** was obtained as a purple solid. Yield: 75%, mp 252-254 °C. ^{1}H-NMR (DMSO-d_6) δ: 2.20 (s, 3H), 3.69 (s, 3H), 3.71 (s, 3H), 4.27 (d, J=6.4 Hz, 2H), 5.61 (s, 1H), 6.84-6.86 (m, 2H), 6.96-6.98 (m, 2H), 12.7 (bs, 1H). MS (ESI): $[M+1]^+$=316.4.

*5-Methyl-2-((4-(trifluoromethyl)benzyl)amino)-[1,2,4]triazolo[1,5-a]pyrimidin-7-ol (**10w**).* Following general procedure A, starting from N^3-(4-(trifluoromethyl)benzyl)-1*H*-1,2,4-triazole-3,5-diamine (**9w**), compound **10w** was obtained as a white solid. Yield: 78%, mp 202-204 °C. ^{1}H-NMR (DMSO-d_6) δ: 2.20 (s, 3H), 4.44 (d, J=6.4 Hz, 2H), 5.62 (s, 1H), 7.02 (t, J=6.4 Hz, 1H), 7.51 (d, J=8.4 Hz, 2H), 7.64 (d, J=8.4 Hz, 2H), 13.0 (bs, 1H). MS (ESI): $[M+1]^+$=324.7.

*4-(((7-Hydroxy-5-methyl-[1,2,4]triazolo[1,5-a]pyrimidin-2-yl)amino)methyl)benzonitrile (**10x**).* Following general procedure A, starting from 4-((5-amino-1*H*-1,2,4-triazol-3-yl)amino)methyl)benzonitrile (**9x**), compound **10x** was obtained as a pink solid. Yield: 83%, mp 250-252 °C. ^{1}H-NMR (DMSO-d_6) δ: 2.20 (s, 3H), 4.43 (d, J=6.4 Hz, 2H), 5.62 (s, 1H), 7.24 (t, J=6.4 Hz, 1H), 7.48 (d, J=8.4 Hz, 2H), 7.75 (d, J=8.4 Hz, 2H), 12.6 (bs, 1H). MS (ESI): $[M+1]^+$=281.3.

*2-((Benzo[d][1,3]dioxol-5-ylmethyl)amino)-5-methyl-[1,2,4]triazolo[1,5-a]pyrimidin-7-ol (**10y**).* Following general procedure A, starting from N^3-(benzo[*d*][1,3]dioxol-5-ylmethyl)-1*H*-1,2,4-triazole-3,5-diamine (**9y**), compound **10y** was obtained as a white solid. Yield: 78%, mp 263-265 °C. ^{1}H-NMR (DMSO-d_6) δ: 2.20 (s, 3H), 4.24 (d, J=6.4 Hz, 2H), 5.62 (s, 1H), 5.94 (s, 2H), 6.78-6.82 (m, 2H), 6.88 (s, 1H), 7.04 (t, J=5.6 Hz, 1H), 12.7 (bs, 1H). MS (ESI): $[M+1]^+$=300.4.

*5-Methyl-2-(phenethylamino)-[1,2,4]triazolo[1,5-a]pyrimidin-7-ol (**10z**).* Following general procedure A, starting from N^3-phenethyl-1*H*-1,2,4-triazole-3,5-diamine (**9z**), compound **10z** was obtained as a pink solid. Yield: 88%, mp 204-206 °C. ^{1}H-NMR (DMSO-d_6) δ: 2.23 (s, 3H), 2.85 (t, J=7.2 Hz, 2H), 3.37-3.39 (m, 2H), 5.64 (s, 1H), 6.61 (t, J=6.4 Hz, 1H), 7.20-7.29 (m, 5H), 12.6 (bs, 1H). MS (ESI): $[M+1]^+$=270.6.

*2-((4-Fluorophenethyl)amino)-5-methyl-[1,2,4]triazolo[1,5-a]pyrimidin-7-ol (**10aa**).* Following general procedure A, starting from N^3-(4-fluorophenethyl)-1*H*-1,2,4-triazole-3,5-diamine (**9aa**), compound **10aa** was obtained as a pink solid. Yield: 81%, mp 270-272 °C. ^{1}H-NMR (DMSO-d_6) δ: 2.21 (s, 3H), 2.82 (t, J=7.2 Hz, 2H), 3.30-3.36 (m, 2H), 5.62 (s, 1H), 6.59 (t, J=6.4 Hz, 1H), 7.08 (t, J=8.4 Hz, 2H), 7.24 (t, J=8.4 Hz, 2H), 12.6 (bs, 1H). MS (ESI): $[M+1]^+$=288.3.

*2-((4-Chlorophenethyl)amino)-5-methyl-[1,2,4]triazolo[1,5-a]pyrimidin-7-ol (**10ab**).* Following general procedure A, starting from N^3-(4-chlorophenethyl)-1*H*-1,2,4-triazole-3,5-diamine (**9ab**), compound **10ab** was obtained as a white solid. Yield: 84%, mp 262-264 °C. ^{1}H-NMR (DMSO-d_6) δ: 2.21 (s, 3H), 2.75 (t, J=7.2 Hz, 2H), 3.16-3.22 (m, 2H), 5.60 (s, 1H), 5.84 (t, J=6.4 Hz, 1H), 7.20 (d, J=8.4 Hz, 2H), 7.30 (d, J=8.4 Hz, 2H), 10.6 (bs, 1H). MS (ESI): $[M+1]^+$=304.7.

*2-((4-Methoxyphenethyl)amino)-5-methyl-[1,2,4]triazolo[1,5-a]pyrimidin-7-ol (**10ac**).* Following general procedure A, starting from N^3-(4-methoxyphenethyl)-1*H*-1,2,4-triazole-3,5-diamine (**9ac**), compound **10ac** was obtained as a pink solid. Yield: 85%, mp 252-254 °C. ^{1}H-NMR (DMSO-d_6) δ: 2.21 (s, 3H), 2.76 (t, J=7.2 Hz, 2H), 3.28-3.30 (m, 2H), 3.70 (s, 3H), 5.62 (s, 1H), 6.56 (t, J=6.4 Hz, 1H), 6.82 (d, J=8.7 Hz, 2H), 7.30 (d, J=8.7 Hz, 2H), 10.7 (bs, 1H). MS (ESI): $[M+1]^+$=300.3.

*5-Methyl-2-((3-phenylpropyl)amino)-[1,2,4]triazolo[1,5-a]pyrimidin-7-ol (**10ad**).* Following general procedure A, starting from N^3-(3-phenylpropyl)-1*H*-1,2,4-triazole-3,5-diamine (**9ad**), compound **10ad** was obtained as a pink solid. Yield: 68%, mp 252-254 °C. ^{1}H-NMR (DMSO-d_6) δ: 1.79-1.84 (m, 2H), 2.20 (s, 3H), 2.62 (t, J=7.2 Hz, 2H), 3.14 (t, J=7.2 Hz, 2H), 5.61 (s, 1H), 6.62 (t, J=6.4 Hz, 1H), 7.14-7.26 (m, 5H), 12.6 (bs, 1H). MS (ESI): $[M+1]^+$=284.3.

Characterization of compounds 11a-ad

*7-Chloro-2-(4-fluorophenyl)-5-methyl-[1,2,4]triazolo[1,5-a]pyrimidine (**11a**).* Following general procedure B, compound **11a** was obtained as a yellow solid. Yield: 78%, mp 154-156 °C. ^{1}H-NMR (DMSO-d_6) δ: 2.63 (s, 3H), 7.40 (t, J=8.8 Hz, 2H), 7.64 (s, 1H), 8.24-8.27 (m, 2H). MS (ESI): $[M+1]^+$=263.5.

*7-Chloro-2-(4-chlorophenyl)-5-methyl-[1,2,4]triazolo[1,5-a]pyrimidine (**11b**).* Following general procedure B, compound **11b** was obtained as a yellow solid. Yield: 81%, mp 173-175 °C. ^{1}H-NMR (DMSO-d_6) δ: 2.64 (s, 3H), 7.63 (dd, J=8.4 and 2.0 Hz, 2H), 7.91 (s, 1H), 8.21 (dd, J=8.4 and 2.0 Hz, 2H). MS (ESI): $[M+1]^+$=279.4.

7-Chloro-5-methyl-2-(p-tolyl)-[1,2,4]triazolo[1,5-a]pyrimidine (11c). Following general procedure B, **11c** was obtained as an orange solid. Yield: 57%, mp 173-175 °C. ^{1}H-NMR (DMSO-*d6*) δ: 2.39 (s, 3H), , 2.62 (s, 3H), 7.36 (d, J=8.4 Hz, 2H), 7.62 (s, 1H), 8.09 (d, J=8.4 Hz, 2H). MS (ESI): $[M+1]^+$=259.2.

7-Chloro-5-methyl-N-phenyl-[1,2,4]triazolo[1,5-a]pyrimidin-2-amine (11d). Following general procedure B, compound **11d** was obtained as a brownish solid. Yield: 75%, mp 135-136 °C. ^{1}H-NMR (DMSO-*d6*) δ: 2.55 (s, 3H), 6.93 (t, J=8.4 Hz, 1H), 7.32 (t, J=8.4 Hz, 2H), 7.41 (s, 1H), 7.69 (d, J=8.4 Hz, 2H), 9.98 (s, 1H). MS (ESI): $[M+1]^+$=260.2.

7-Chloro-N-(4-fluorophenyl)-5-methyl-[1,2,4]triazolo[1,5-a]pyrimidin-2-amine (11e). Following general procedure B, compound **11e** was obtained as a yellow solid. Yield: 53%, mp 192-194 °C. ^{1}H-NMR (DMSO-*d6*) δ: 2.55 (s, 3H), 7.16 (t, J=8.8 Hz, 2H), 7.41 (s, 1H), 7.68-7.72 (m, 2H), 10.0 (s, 1H). MS (ESI): $[M+1]^+$=278.3.

7-Chloro-N-(4-chlorophenyl)-5-methyl-[1,2,4]triazolo[1,5-a]pyrimidin-2-amine (11f). Following general procedure B, compound **11f** was obtained as a yellow solid. Yield: 69%, mp 186-188 °C. ^{1}H-NMR (DMSO-*d6*) δ: 2.34 (s, 3H), 6,96 (d, J=8.4 Hz, 2H), 7.12 (s, 1H), 7.56 (d, J=8.4 Hz, 2H), 9.74 (s, 1H). MS (ESI): $[M+1]^+$=295.2.

7-Chloro-5-methyl-N-(p-tolyl)-[1,2,4]triazolo[1,5-a]pyrimidin-2-amine (11g). Following general procedure B, compound **11g** was obtained as a yellow solid. Yield: 54%, mp 201-202 °C. ^{1}H-NMR (DMSO-*d6*) δ: 2.25 (s, 3H), 2.55 (s, 3H), 7.10 (d, J=8.8 Hz, 2H), 7.39 (s, 1H), 7.57 (d, J=8.8 Hz, 2H), 9.87 (s, 1H). MS (ESI): $[M+1]^+$=274.3.

7-Chloro-N-(4-methoxyphenyl)-5-methyl-[1,2,4]triazolo[1,5-a]pyrimidin-2-amine (11h). Following general procedure B, compound **11h** was obtained as a brownish solid. Yield: 73%, mp 242-244 °C. ^{1}H-NMR (DMSO-*d6*) δ: 2.42 (s, 3H), 3.72 (s, 3H), 6.90 (d, J=8.8 Hz, 2H), 7.38 (s, 1H), 7.59 (d, J=8.8 Hz, 2H), 9.77 (s, 1H). MS (ESI): $[M+1]^+$=290.4.

N-Benzyl-7-chloro-5-methyl-[1,2,4]triazolo[1,5-a]pyrimidin-2-amine (11i). Following general procedure B, compound **11i** was obtained as a white solid. Yield: 72%, mp 232-234 °C. ^{1}H-NMR ($CDCl_3$) δ: 2.62 (s, 3H), 4.67 (s, 2H), 3.50 (bs, 1H), 6.92 (s, 1H), 7.28 (d, J=6.4 Hz, 2H), 7.29 (t, J=6.4 Hz, 1H), 7.39 (d, J=6.4 Hz, 2H). MS (ESI): $[M+1]^+$=274.2.

7-Chloro-5-methyl-N-(pyridin-4-ylmethyl)-[1,2,4]triazolo[1,5-a]pyrimidin-2-amine (11j). Following general procedure B, compound **11j** was obtained as a brown solid. Yield: 44%, mp 210-212 °C. ^{1}H-NMR (DMSO-*d6*) δ: 3.12 (s, 3H), 4.44 (d, J=6.4 Hz, 2H), 5.78 (s, 1H), 7.34 (d, J=6.2 Hz, 2H), 7.34 (d, J=6.2 Hz, 2H), 7.34 (d, J=6.2 Hz, 2H), 7.83 (t, J=6.4 Hz, 1H). MS (ESI): $[M+1]^+$=275.1.

7-Chloro-5-methyl-N-(pyridin-3-ylmethyl)-[1,2,4]triazolo[1,5-a]pyrimidin-2-amine (11k). Following general procedure B, compound **11k** was obtained as a brown solid. Yield: 57%, mp 234-236 °C. ^{1}H-NMR (DMSO-*d6*) δ: 2.22 (s, 3H), 4.54 (d, J=6.2 Hz, 2H), 7.26 (s, 1H), 7.36 (dd, J=7.2 and 4.8 Hz, 1H), 7.82 (d, J=7.70 Hz, 1 H), 8.46 (dd, J=4.8 and 1.6 Hz, 1H), 8.56 (d, J=1.6 Hz, 1H), 8.63 (t, J=6.2 Hz, 1H). MS (ESI): $[M+1]^+$=275.1.

7-Chloro-N-(4-fluorobenzyl)-5-methyl-[1,2,4]triazolo[1,5-a]pyrimidin-2-amine (11l). Following general procedure B, compound **11l** was obtained as a brown solid. Yield: 72%, mp 156-158 °C. ^{1}H-NMR (DMSO-*d6*) δ: 2.50 (s, 3H), 4.46 (d, J=6.4 Hz, 2H), 7.11-7.18 (m, 1H), 7.25-7.27 (m, 1H), 7.26 (s, 1H), 7.38-7.43 (m, 2H), 7.68 (t, J=6.5 Hz, 1H). MS (ESI): $[M+1]^+$=292.05.

*7-Chloro-N-(3-fluorobenzyl)-5-methyl-[1,2,4]triazolo[1,5-a]pyrimidin-2-amine (**11m**).* Following general procedure B, compound **11m** was obtained as a brown solid. Yield: 58%, mp 162-164 °C. ^{1}H-NMR (DMSO-*d*$_6$) δ: 2.52 (s, 3H), 4.46 (d, J=6.4 Hz, 2H), 5.74 (s, 1H), 7.22 (dt, J=8.5 and 2.9 Hz, 1H), 7.13-7.22 (m, 2H), 7.32-7.42 (m, 1H), 7.72 (t, J=6.5 Hz, 1H). MS (ESI): $[M+1]^+$=292.01.

*7-Chloro-N-(4-chlorobenzyl)-5-methyl-[1,2,4]triazolo[1,5-a]pyrimidin-2-amine (**11n**).* Following general procedure B, compound **11n** was obtained as a brown solid. Yield: 55%, mp 174-176 °C. ^{1}H-NMR ($CDCl_3$) δ: 2.62 (s, 3H), 4.63 (s, 2H), 5.84 (bs, 1H), 6.93 (s, 1H), 7.30 (d, J=9.2 Hz, 2H), 7.36 (d, J=9.2 Hz, 2H). MS (ESI): $[M+1]^+$=307.9.

*7-Chloro-N-(3-chlorobenzyl)-5-methyl-[1,2,4]triazolo[1,5-a]pyrimidin-2-amine (**11o**).* Following general procedure B, compound **11o** was obtained as a brown solid. Yield: 52%, mp 163-165 °C. ^{1}H-NMR (DMSO-*d*$_6$) δ: 2.23 (s, 3H), 4.47 (d, J=6.4 Hz, 2H), 5.76 (s, 1H), 7.26-7.40 (m, 3H), 7.42 (s, 1H), 7.75 (t, J=6.5 Hz, 1H). MS (ESI): $[M+1]^+$=308.0.

*7-Chloro-5-methyl-N-(4-methylbenzyl)-[1,2,4]triazolo[1,5-a]pyrimidin-2-amine (**11p**).* Following general procedure B, **11p** was obtained as a pink solid. Yield: 58%, mp 180-182 °C. ^{1}H-NMR (DMSO-*d*$_6$) δ: 2.24 (s, 3H), 2.46 (s, 3H), 4.39 (d, J=6.0 Hz, 2H), 7.08 (d, J=7.6 Hz, 2H), 7.21 (d, J=7.6 Hz, 2H), 7.23 (s, 1H), 7.60 (t, J=6.0 Hz, 1H). MS (ESI): $[M+1]^+$=288.4.

*7-Chloro-5-methyl-N-(3-methylbenzyl)-[1,2,4]triazolo[1,5-a]pyrimidin-2-amine (**11q**).* Following general procedure B, **11q** was obtained as a pink solid. Yield: 73%, mp 154-156 °C. ^{1}H-NMR ($CDCl_3$) δ: 2.34 (s, 3H), 2.61(s, 3H), 4.63 (s, 2H), 5.62 (bs, 1H), 6.90 (s, 1H), 7.02 (dd, J=7.6 and 1.8 Hz, Hz, 1H), 7.16-7.21 (m, 3H). MS (ESI): $[M+1]^+$=287.8.

*7-Chloro-5-methyl-N-(2-methylbenzyl)-[1,2,4]triazolo[1,5-a]pyrimidin-2-amine (**11r**).* Following general procedure B, **11r** was obtained as a pink solid. Yield: 78%, mp 120-122 °C. ^{1}H-NMR ($CDCl_3$) δ: 2.40 (s, 3H), 2.63 (s, 3H), 4.64 (s, 2H), 6.16 (bs, 1H), 6.98 (s, 1H), 7.16-7.19 (m, 3H), 7.36 (dd, J=6.0 and 1.6 Hz, Hz, 1H). MS (ESI): $[M+1]^+$=287.8.

*7-Chloro-N-(4-methoxybenzyl)-5-methyl-[1,2,4]triazolo[1,5-a]pyrimidin-2-amine (**11s**).* Following general procedure B, compound **11s** was obtained as a pink solid. Yield: 63%, mp 210-212 °C. ^{1}H-NMR (DMSO-*d*$_6$) δ: 2.46 (s, 3H), 3.69 (s, 3H), 4.37 (d, J=6.4 Hz, 2H), 6.84 (d, J=8.4 Hz, 2H), 7.23 (s, 1H), 7.25 (d, J=8.4 Hz, 2H), 7.59 (t, J=6.4 Hz, 1H). MS (ESI): $[M+1]^+$=303.8.

*7-Chloro-N-(3-methoxybenzyl)-5-methyl-[1,2,4]triazolo[1,5-a]pyrimidin-2-amine (**11t**).* Following general procedure B, compound **11t** was obtained as a purple solid. Yield: 54%, mp 180-182 °C. ^{1}H-NMR ($CDCl_3$) δ: 2.61 (s, 3H), 3.80 (s, 3H), 4.64 (s, 2H), 6.12 (bs. 1H), 6.84 (d, J=8.4 Hz, 1H), 6.93-6.97 (m, 3H), 7.24 (t, J=8.4 Hz, 1H). MS (ESI): $[M+1]^+$=303.8.

*7-Chloro-N-(2-methoxybenzyl)-5-methyl-[1,2,4]triazolo[1,5-a]pyrimidin-2-amine (**11u**).* Following general procedure B, compound **11u** was obtained as a purple solid. Yield: 61%, mp 192-194 °C. ^{1}H-NMR ($CDCl_3$) δ: 2.59 (s, 3H), 3.87 (s, 3H), 4.64 (s, 2H), 5.74 (bs. 1H), 6.81-6.94 (m, 3H), 7.21-7.32 (m, 1H), 7.42 (d, J=8.2 Hz, 1H). MS (ESI): $[M+1]^+$=303.7.

*7-Chloro-N-(3,4-dimethoxybenzyl)-5-methyl-[1,2,4]triazolo[1,5-a]pyrimidin-2-amine (**11v**).* Following general procedure B, compound **11v** was obtained as a pink solid. Yield: 68%, mp 148-150 °C. ^{1}H-NMR ($CDCl_3$) δ: 2.64 (s, 3H), 3.84 (s, 3H), 3.88 (s, 3H), 4.58 (s, 2H), 6.80 (d, J=8.0 Hz, 1H), 6.94 (d, J=8.0 Hz, 1H), 6.96 (s, 1H), 7.06 (s, 1H). MS (ESI): $[M+1]^+$=334.8.

7-Chloro-5-methyl-N-(4-(trifluoromethyl)benzyl)-[1,2,4]triazolo[1,5-a]pyrimidin-2-amine ***(11w)***. Following general procedure B, compound **11w** was obtained as a pink solid. Yield: 66%, mp 166-168 °C. ^{1}H-NMR ($CDCl_3$) δ: 2.64 (s, 3H), 3.00 (bs, 1H), 4.72 (s, 2H), 7.02 (s, 1H), 7.54 (d, J=8.4 Hz, 2H), 7.58 (d, J=8.4 Hz, 2H). MS (ESI): $[M+1]^+$=342.3.

4-(((7-Chloro-5-methyl-[1,2,4]triazolo[1,5-a]pyrimidin-2-yl)amino)methyl)benzonitrile ***(11x)***. Following general procedure B, compound **11x** was obtained as an orange solid. Yield: 66%, mp 208-210 °C. ^{1}H-NMR ($CDCl_3$) δ: 2.62 (s, 3H), 4.73 (s, 2H), 6.00 (bs, 1H), 6.95 (s, 1H), 7.50 (d, J=8.4 Hz, 2H), 7.61 (d, J=8.4 Hz, 2H). MS (ESI): $[M+1]^+$=299.7.

N-(benzo[d][1,3]dioxol-5-ylmethyl)-7-chloro-5-methyl-[1,2,4]triazolo[1,5-a]pyrimidin-2-amine ***(11y)***. Following general procedure B, compound **11y** was obtained as a pink solid. Yield: 74%, mp 168-170 °C. ^{1}H-NMR ($CDCl_3$) δ: 2.60 (s, 3H), 4.56 (s, 2H), 5.63 (s, 1H), 5.94 (s, 2H), 6.75 (d, J=8.0 Hz, 1H), 6.82 (dd, J=8.0 and 2,4 Hz, 1H), 6.89-6.91 (m, 2H). MS (ESI): $[M+1]^+$=318.7.

7-Chloro-5-methyl-N-phenethyl-[1,2,4]triazolo[1,5-a]pyrimidin-2-amine ***(11z)***. Following general procedure B, compound **11z** was obtained as a white solid. Yield: 67%, mp 118-120 °C. ^{1}H-NMR ($CDCl_3$) δ: 2.61 (s, 3H), 2.98 (t, J=7.2 Hz, 2H), 3.70 (t, J=7.2 Hz, 2H), 5.42 (bs, 1H), 6.90 (s, 1H), 7.23-7.32 (m, 5H). MS (ESI): $[M+1]^+$=288.5.

7-Chloro-N-(4-fluorophenethyl)-5-methyl-[1,2,4]triazolo[1,5-a]pyrimidin-2-amine ***(11aa)***. Following general procedure B, compound **11aa** was obtained as a brown solid. Yield: 58%, mp 186-188 °C. ^{1}H-NMR ($CDCl_3$) δ: 2.61 (s, 3H), 2.95 (t, J=7.2 Hz, 2H), 3.70 (t, J=7.2 Hz, 2H), 5.28 (bs, 1H), 6.88 (s, 1H), 6.99 (d, J=8.4 Hz, 2H), 7.18-7.23 (m, 2H). MS (ESI): $[M+1]^+$=306.7.

7-Chloro-N-(4-chlorophenethyl)-5-methyl-[1,2,4]triazolo[1,5-a]pyrimidin-2-amine ***(11ab)***. Following general procedure B, compound **11ab** was obtained as a pink solid. Yield: 64%, mp 180-182 °C. ^{1}H-NMR ($CDCl_3$) δ: 2.63 (s, 3H), 2.96 (t, J=7.2 Hz, 2H), 3.70 (t, J=7.2 Hz, 2H), 5.82 (bs, 1H), 6.97 (s, 1H), 7.17 (d, J=8.4 Hz, 2H), 7.26 (d, J=8.4 Hz, 2H). MS (ESI): $[M+1]^+$=323.3.

7-Chloro-N-(4-methoxyphenethyl)-5-methyl-[1,2,4]triazolo[1,5-a]pyrimidin-2-amine ***(11ac)***. Following general procedure B, compound **11ac** was obtained as a white solid. Yield: 64%, mp 186-188 °C. ^{1}H-NMR ($CDCl_3$) δ: 2.60 (s, 3H), 2.91 (t, J=7.2 Hz, 2H), 3.69 (t, J=7.2 Hz, 2H), 3.79 (s, 3H), 5.22 (bs, 1H), 6.83 (d, J=8.4 Hz, 2H), 6.86 (s, 1H), 7.15 (d, J=8.4 Hz, 2H). MS (ESI): $[M+1]^+$=318.3.

7-Chloro-5-methyl-N-(3-phenylpropyl)-[1,2,4]triazolo[1,5-a]pyrimidin-2-amine ***(11ad)***. Following general procedure B, compound **11ad** was obtained as a black solid. Yield: 54%, mp 142-144 °C. ^{1}H-NMR (DMSO-d_6) δ: 1.96-2.04 (m, 2H), 2.61 (s, 3H), 2.74 (t, J=7.2 Hz, 2H), 3.47 (t, J=7.2 Hz, 2H), 5.60 (bs, 1H), 7.16-7.26 (m, 6H). MS (ESI): $[M+1]^+$=302.3.

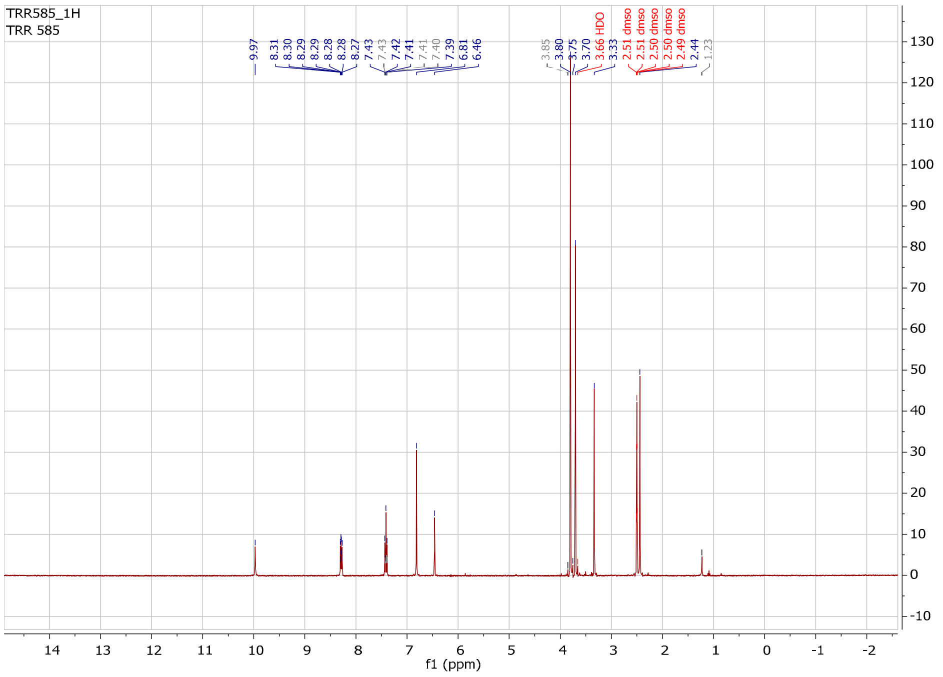

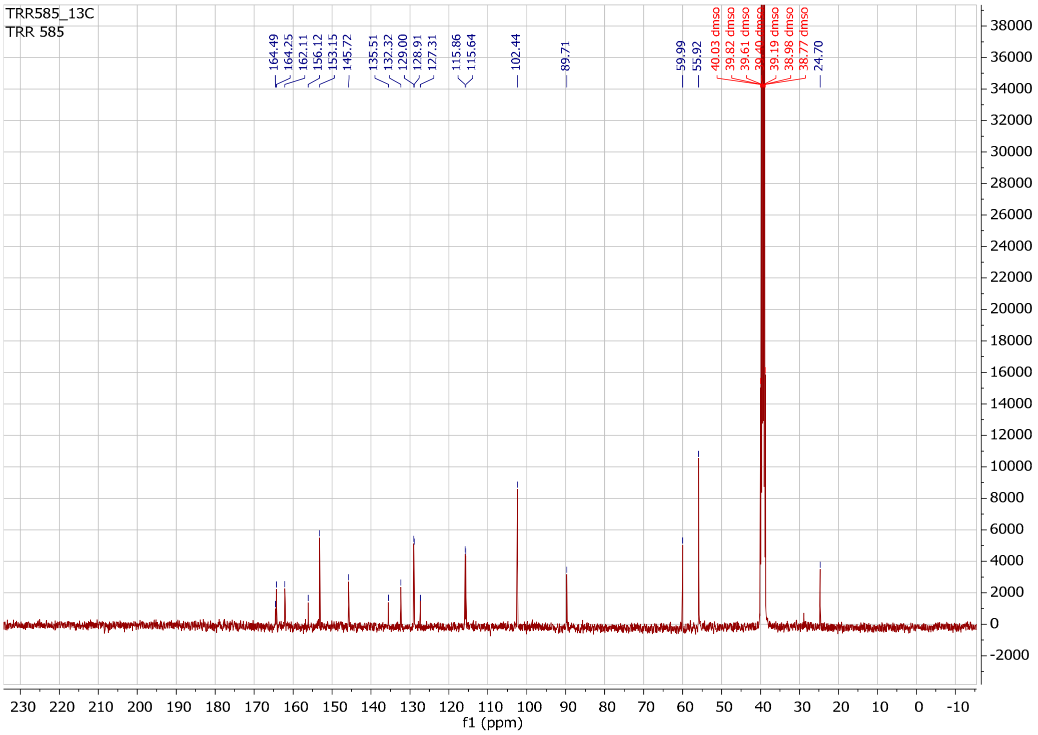

^{1}H-NMR and ^{13}C-NMR spectra of compound **7a**

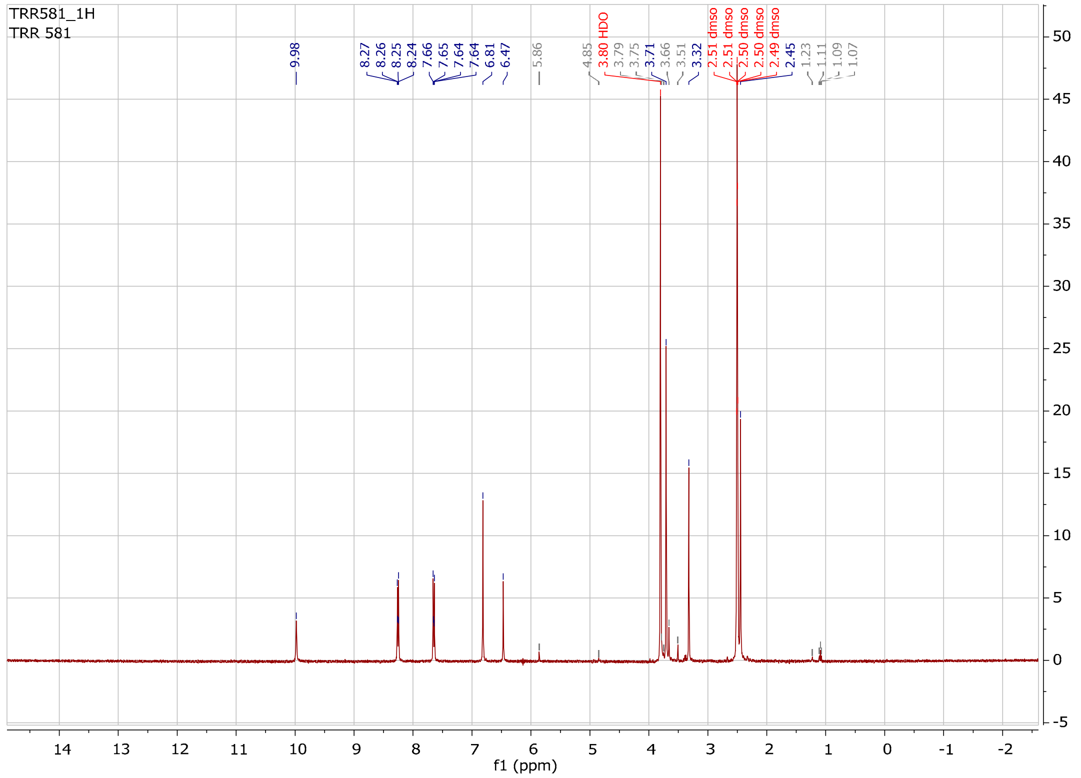

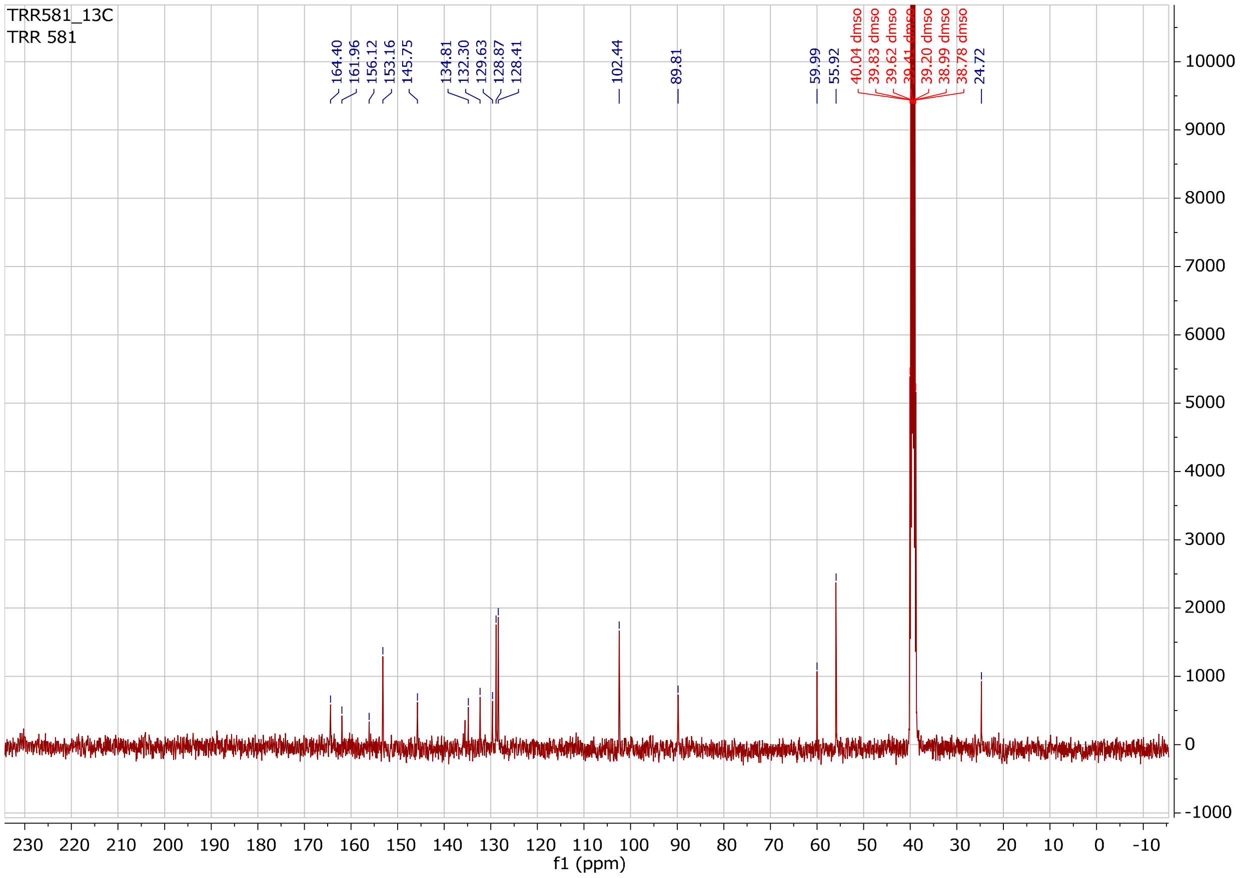

^{1}H-NMR and ^{13}C-NMR spectra of compound **7b**

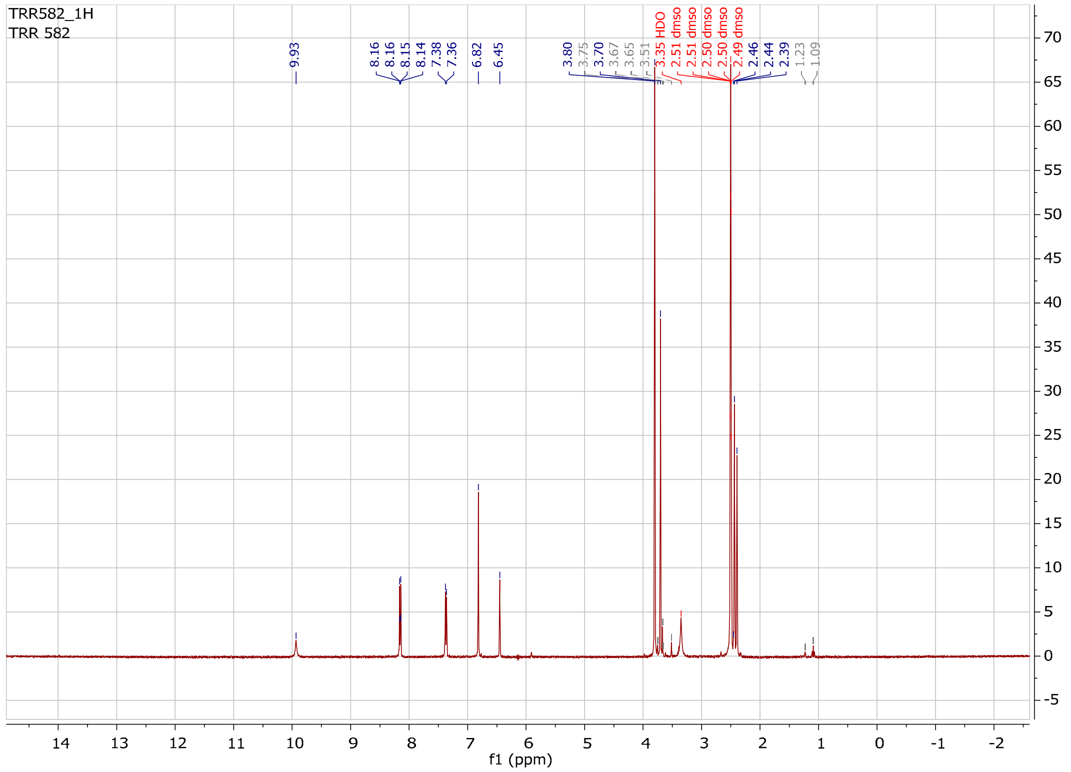

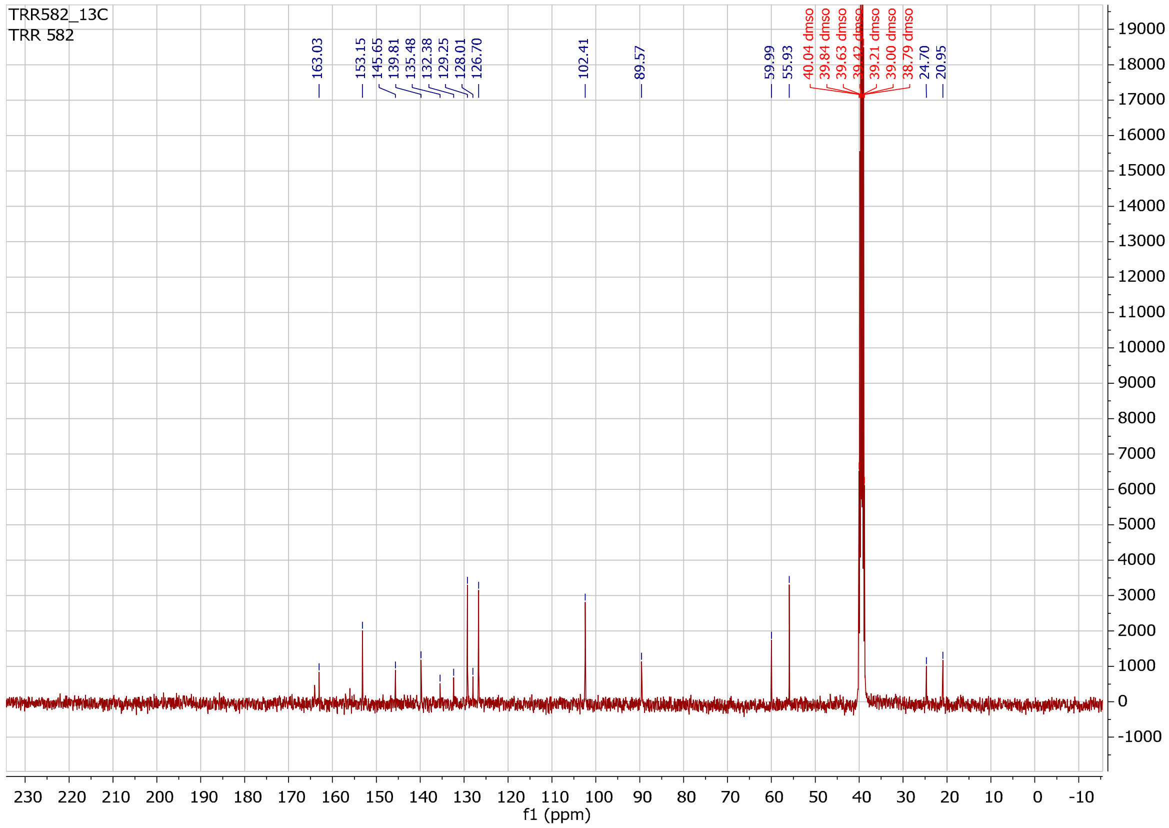

^{1}H-NMR and ^{13}C-NMR spectra of compound **7c**

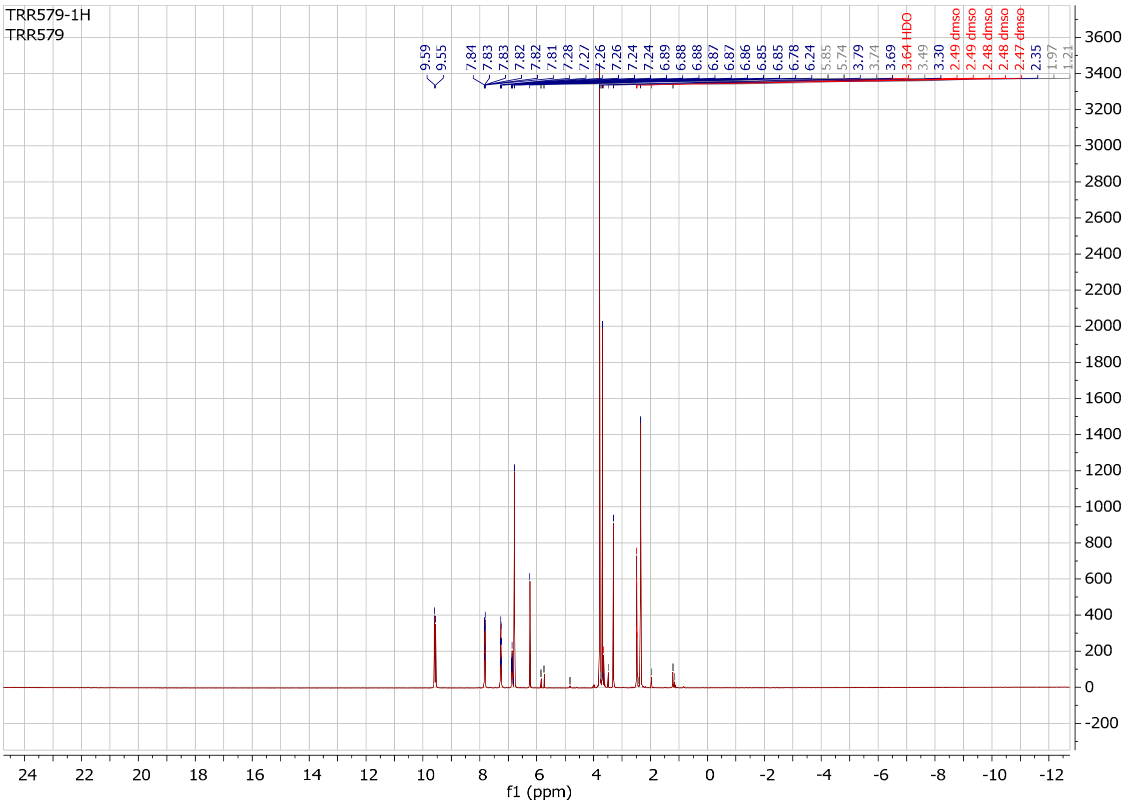

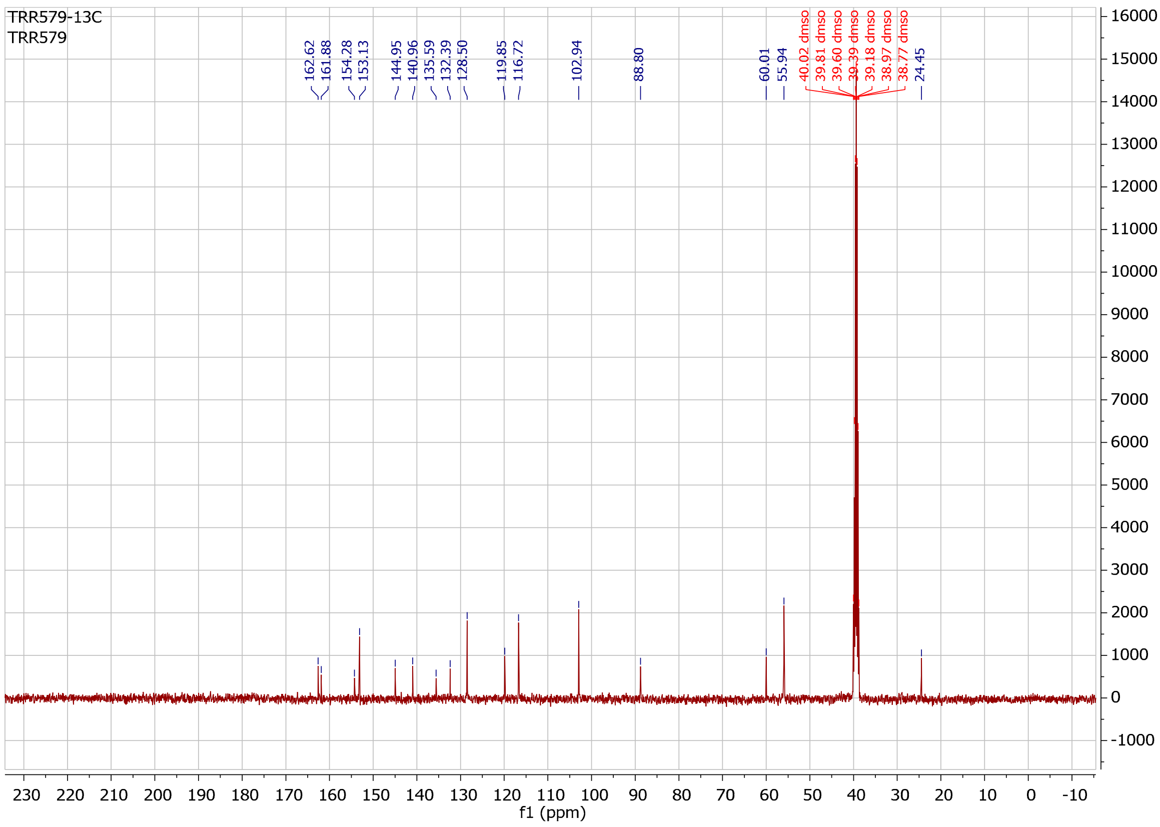

^{1}H-NMR and ^{13}C-NMR spectra of compound **7d**

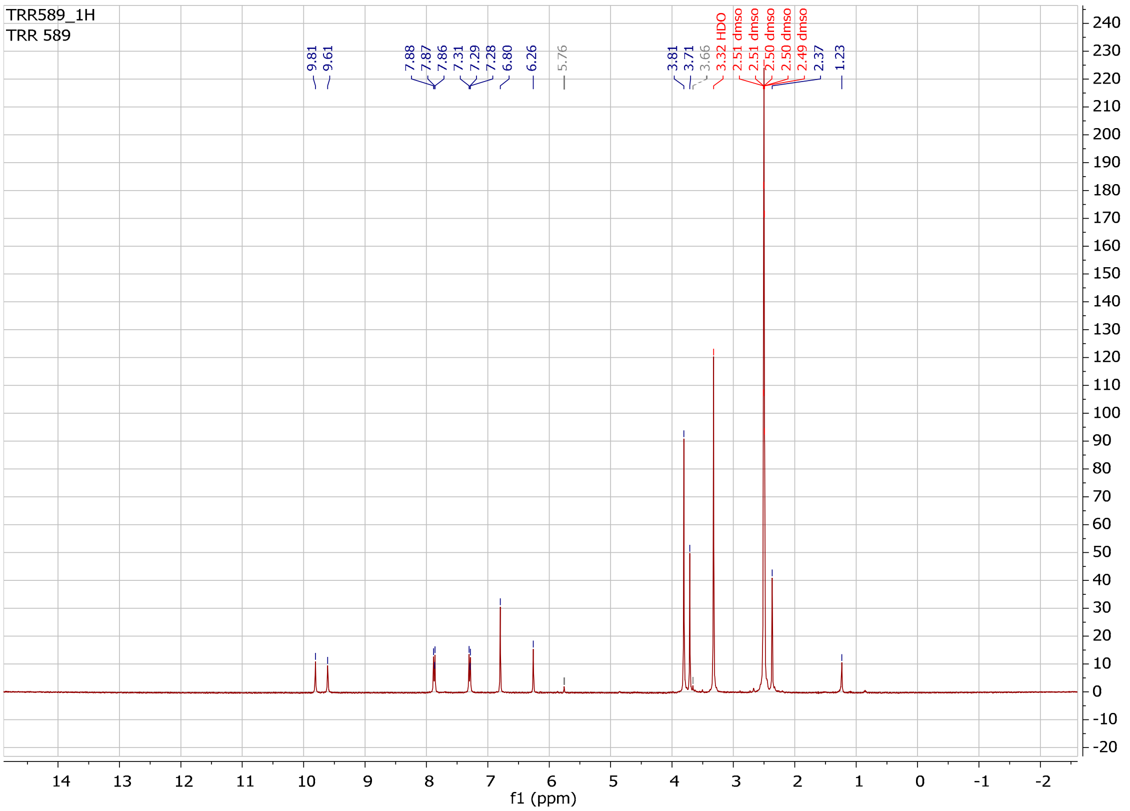

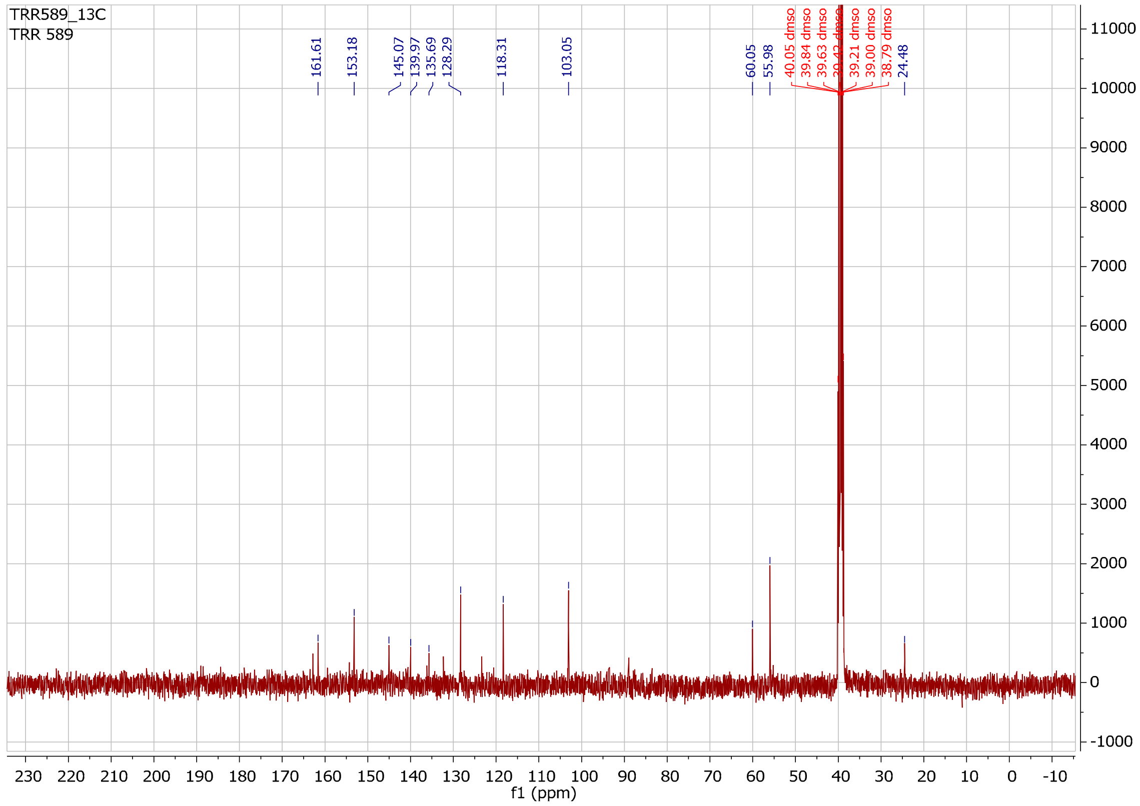

^{1}H-NMR and ^{13}C-NMR spectra of compound **7f**

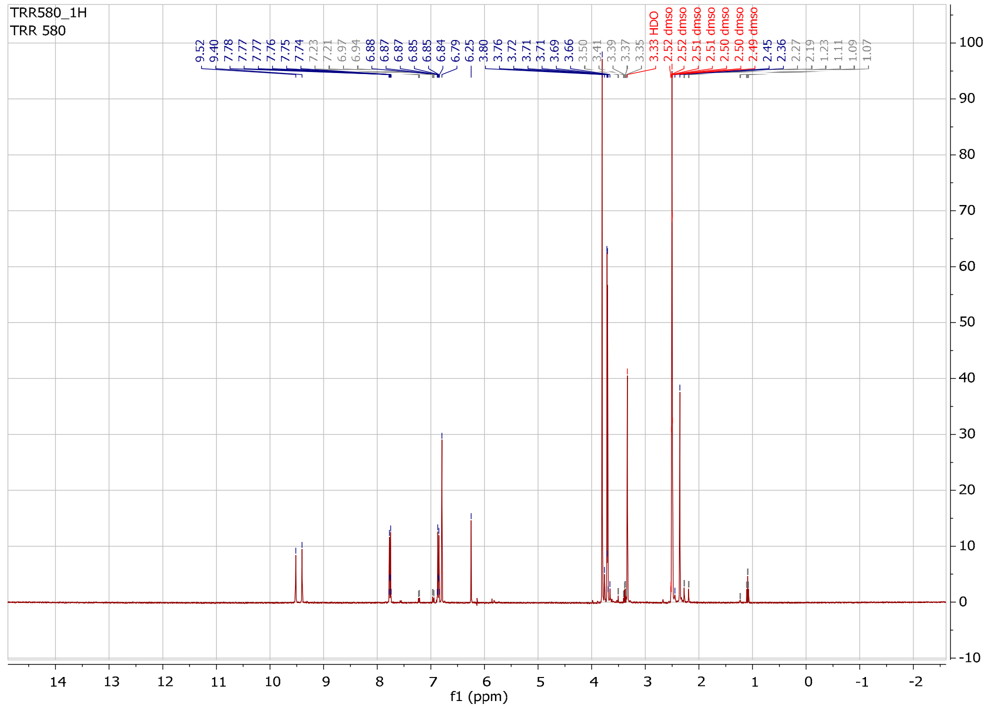

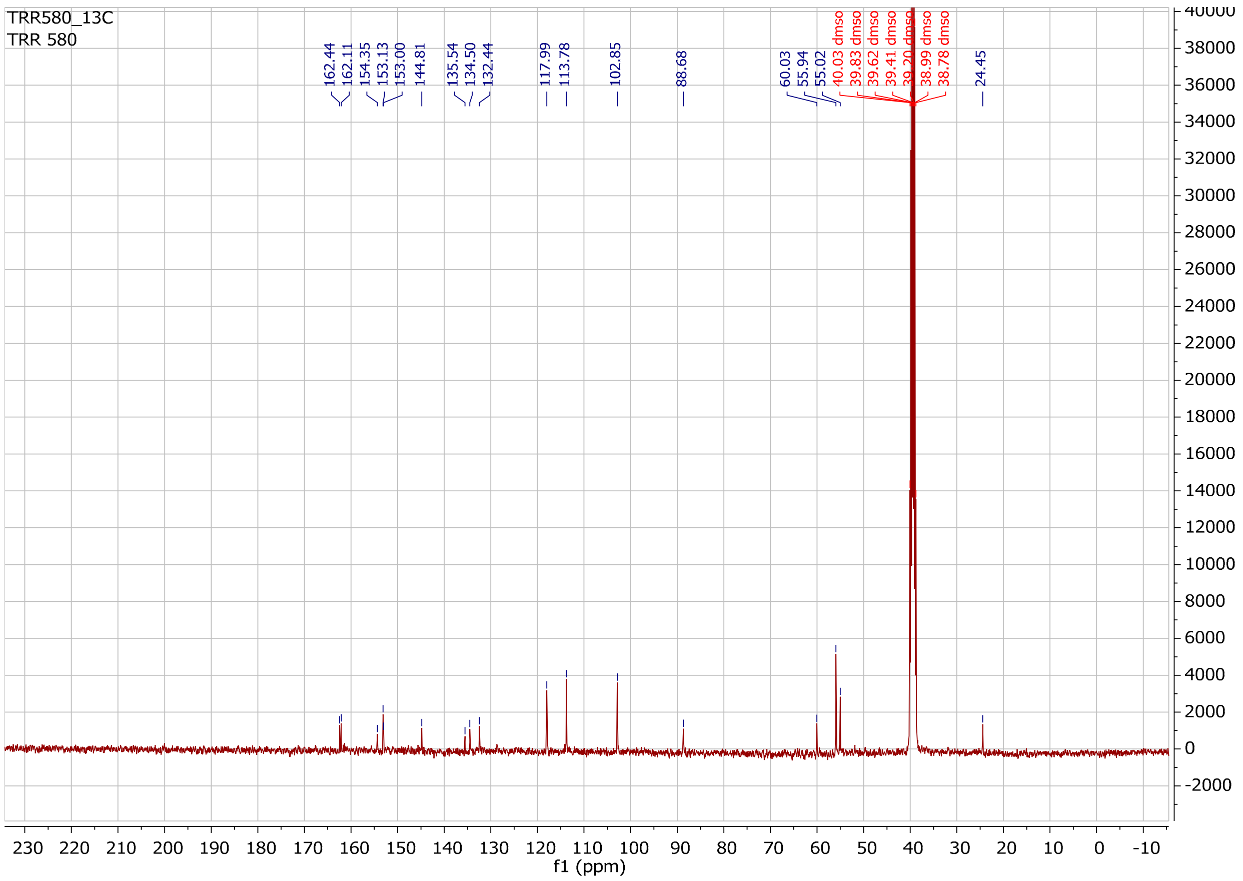

^{1}H-NMR and ^{13}C-NMR spectra of compound **7h**

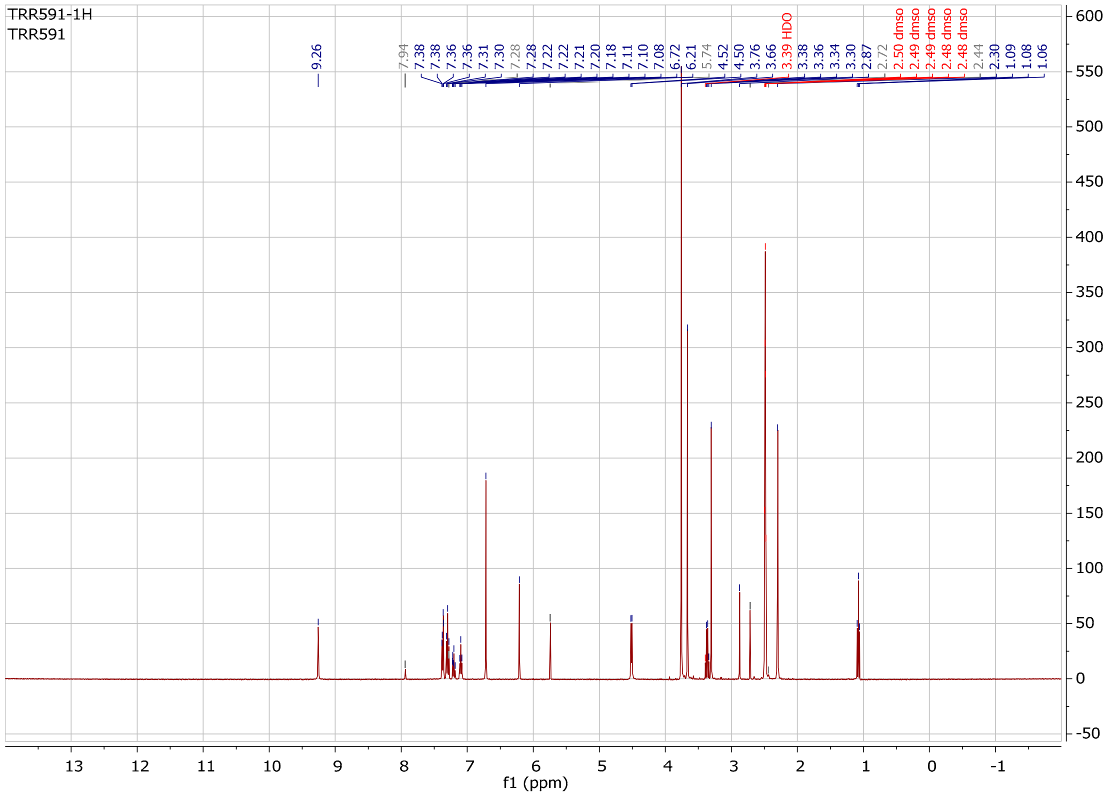

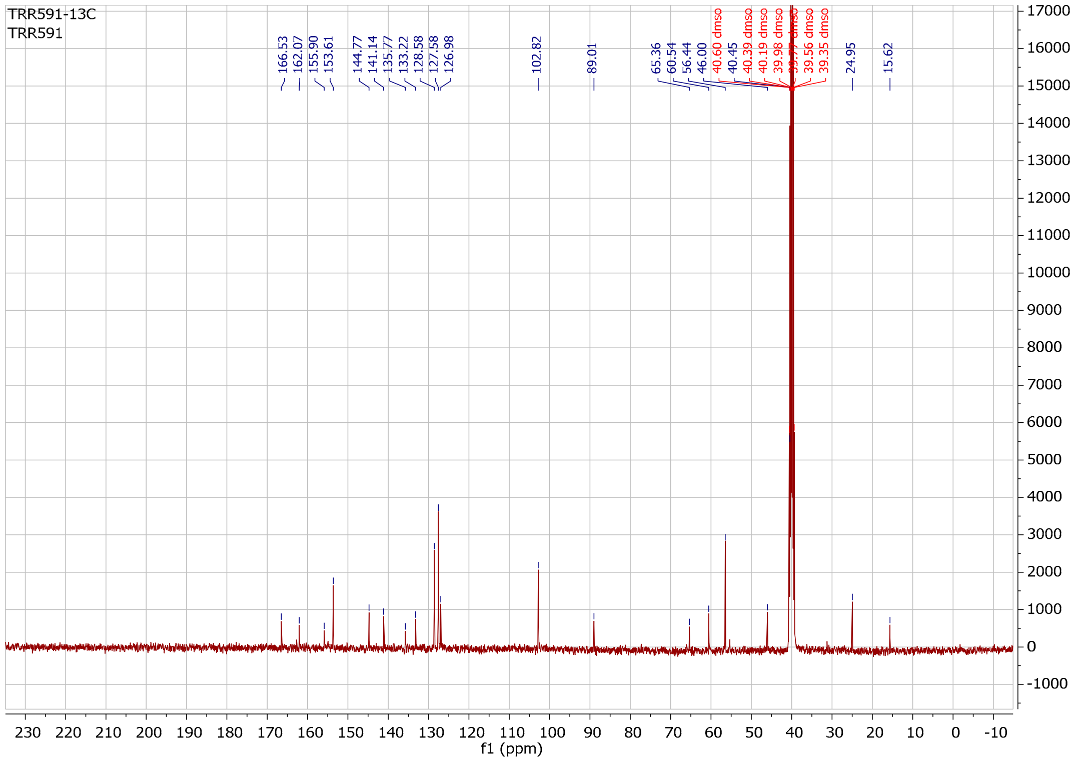

^{1}H-NMR and ^{13}C-NMR spectra of compound **7i**

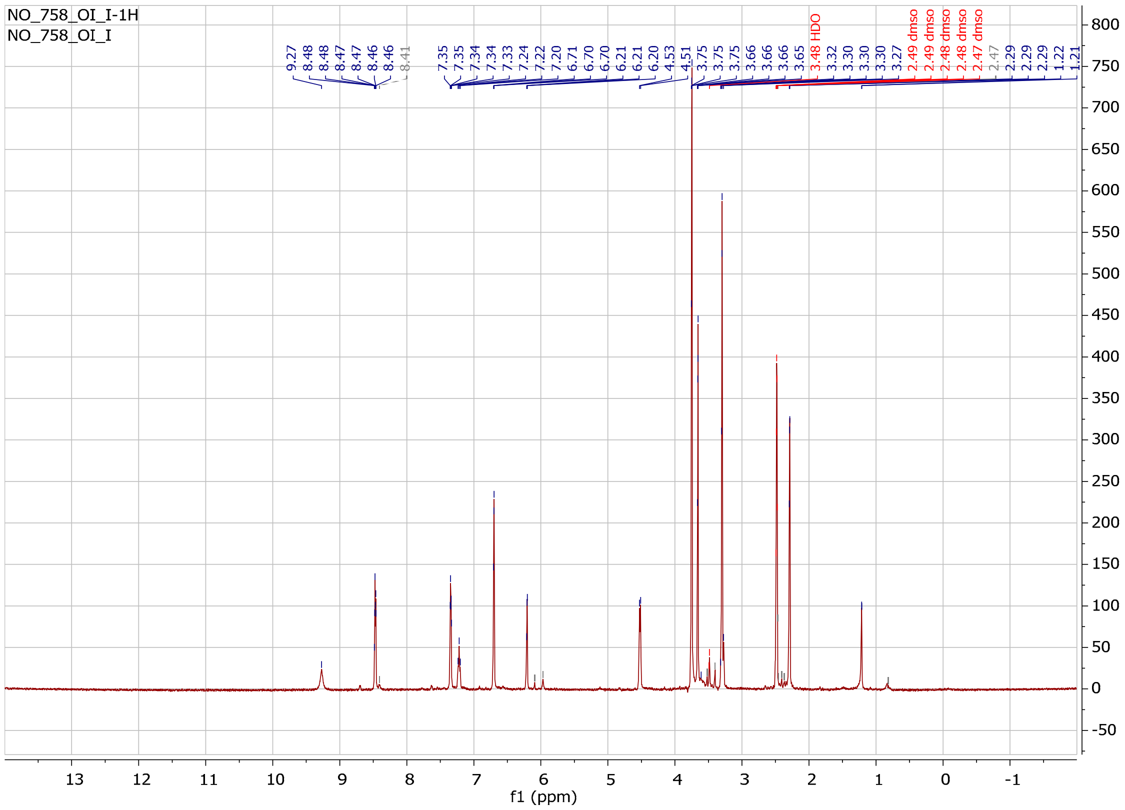

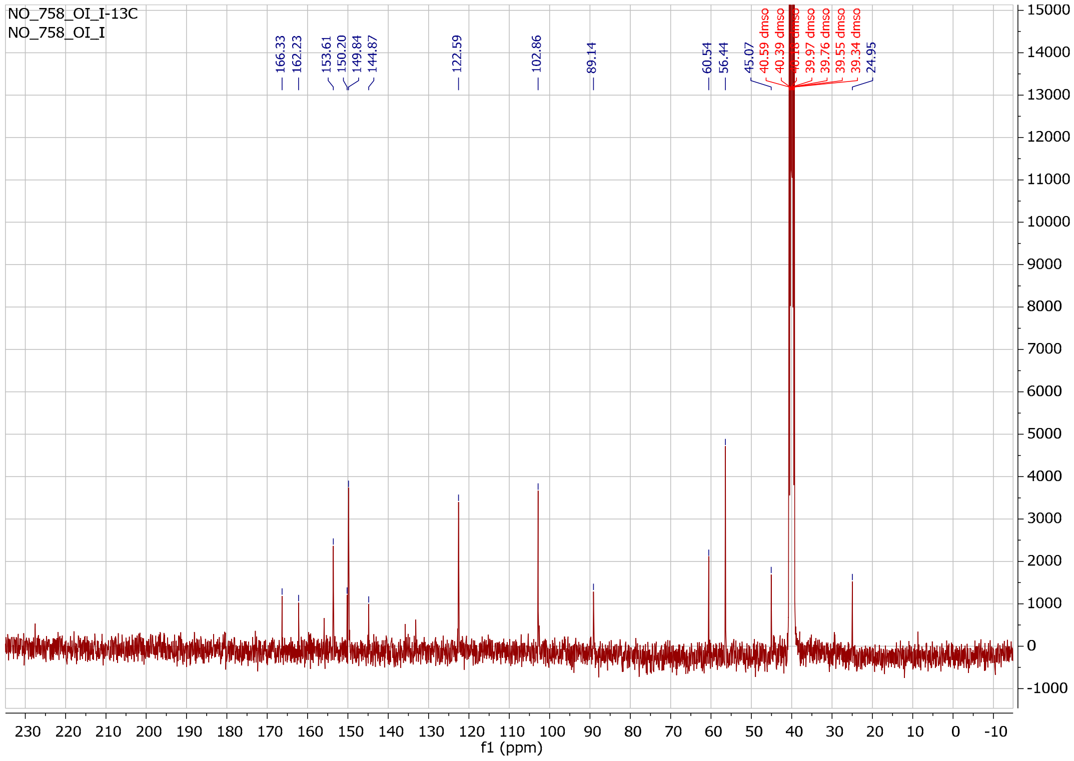

^{1}H-NMR and ^{13}C-NMR spectra of compound **7j**

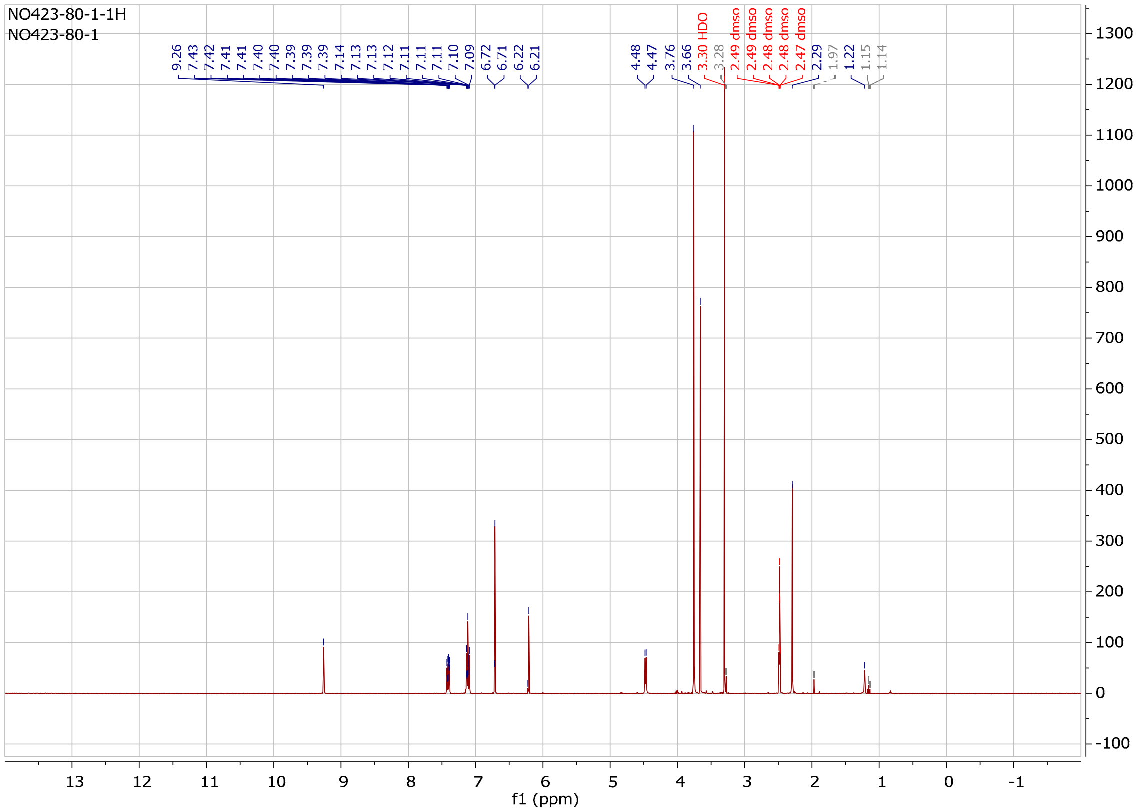

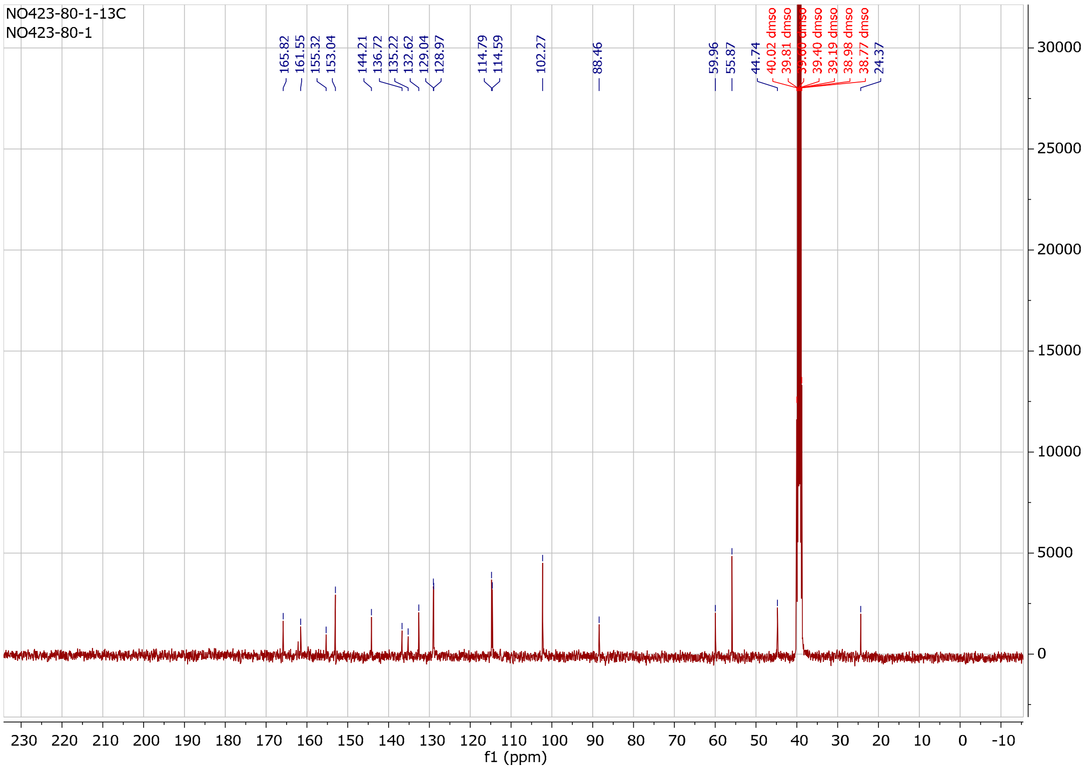

^{1}H-NMR and ^{13}C-NMR spectra of compound **7l**

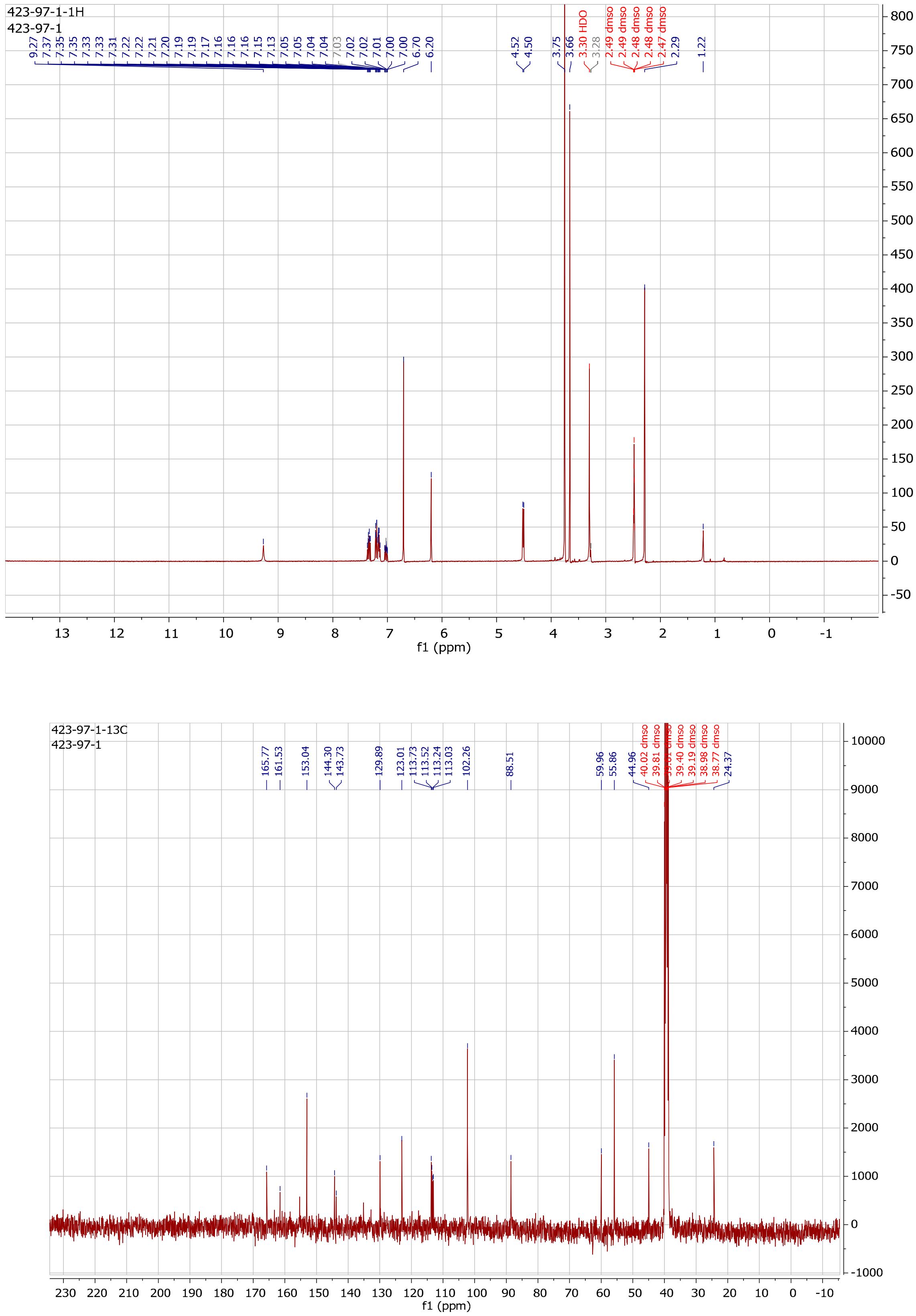

^{1}H-NMR and ^{13}C-NMR spectra of compound **7m**

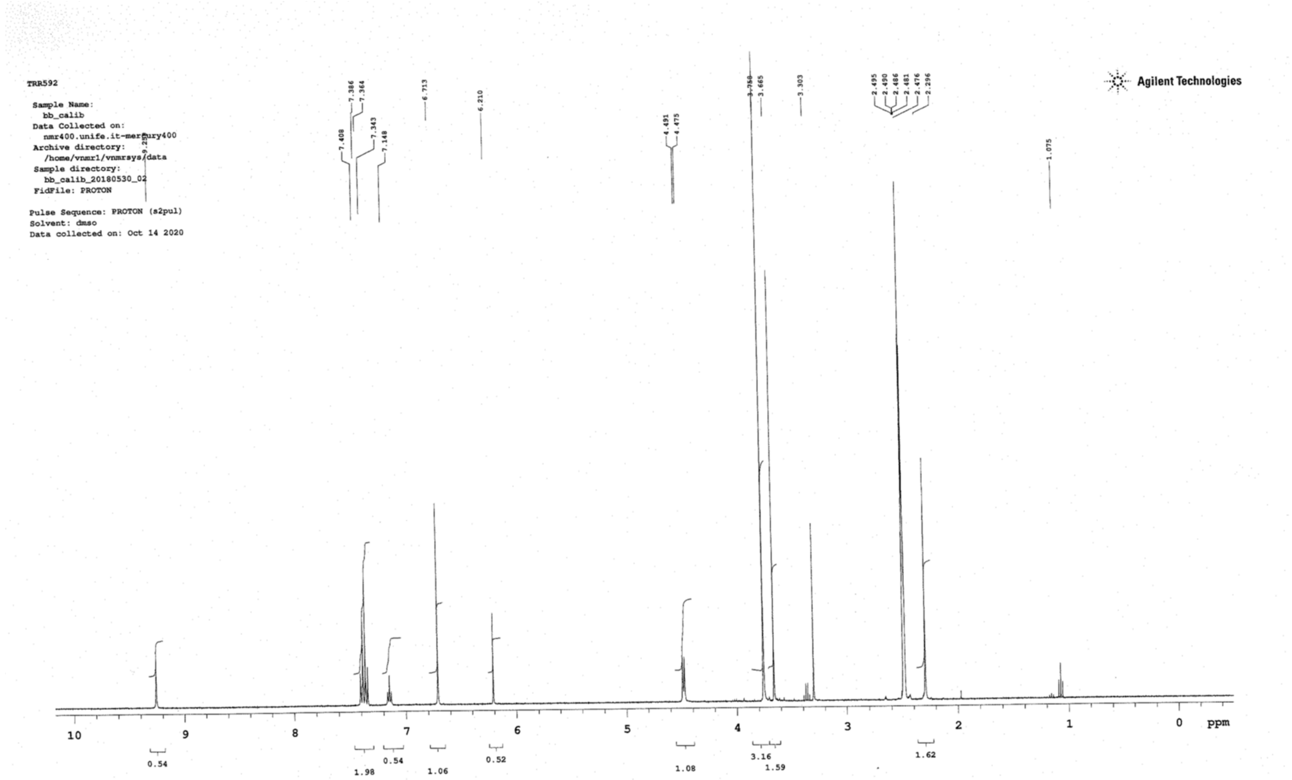

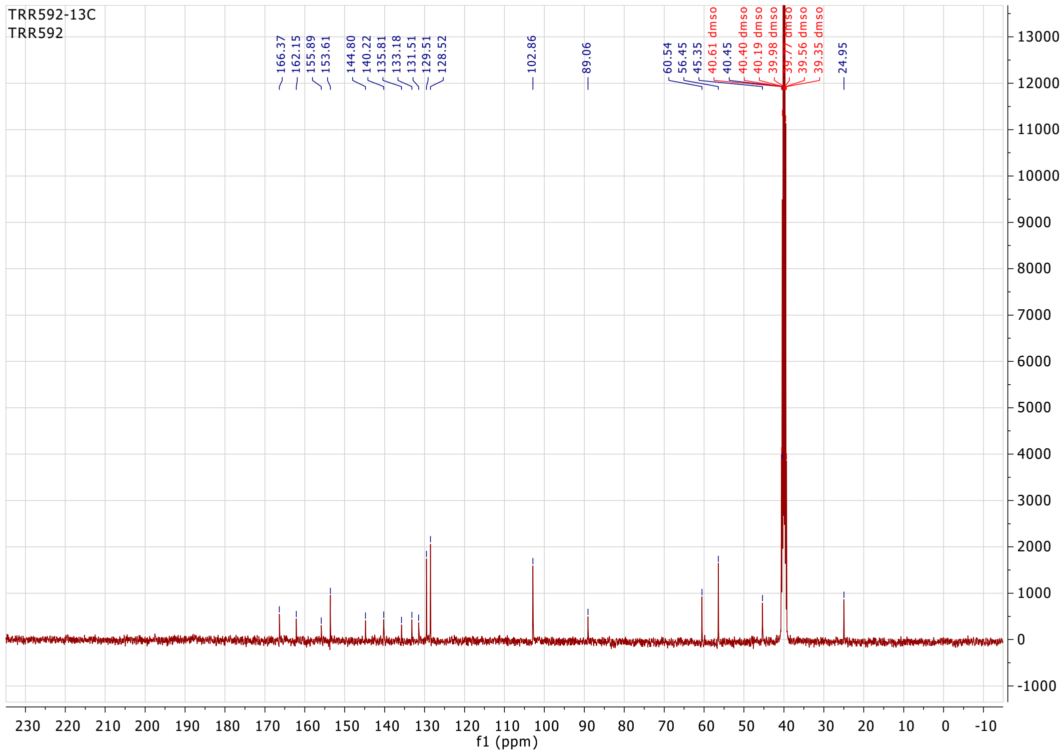

^{1}H-NMR and ^{13}C-NMR spectra of compound **7n**

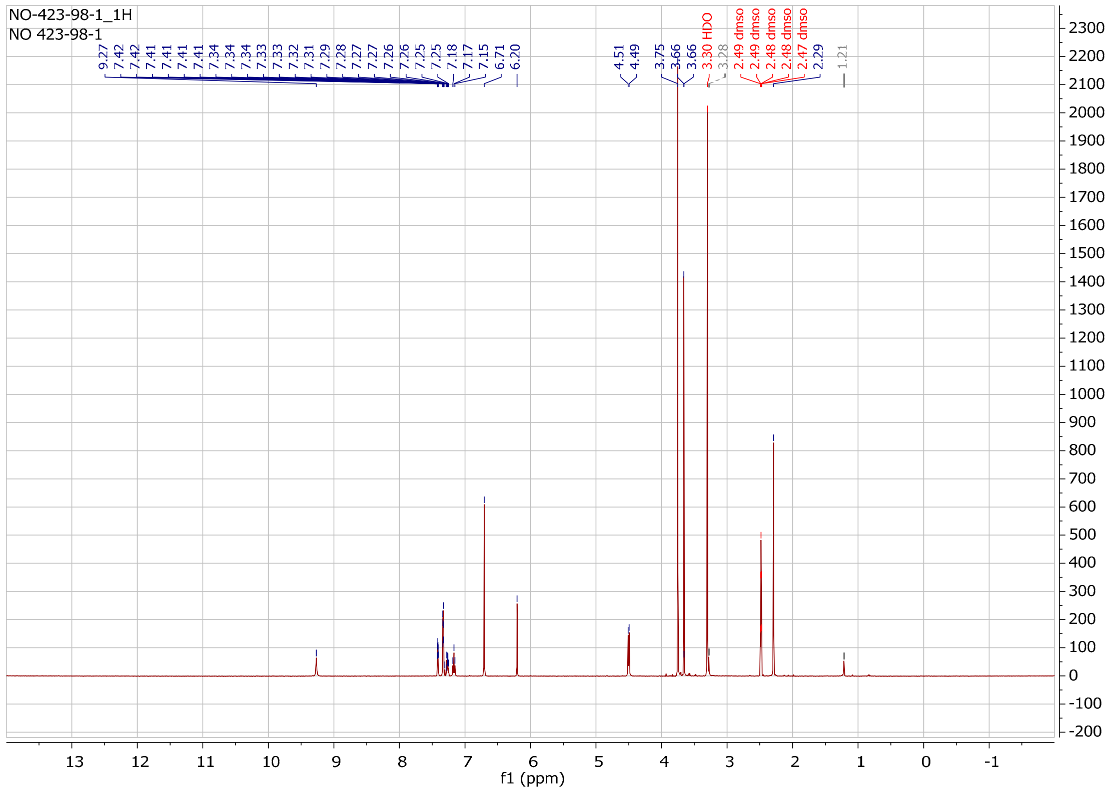

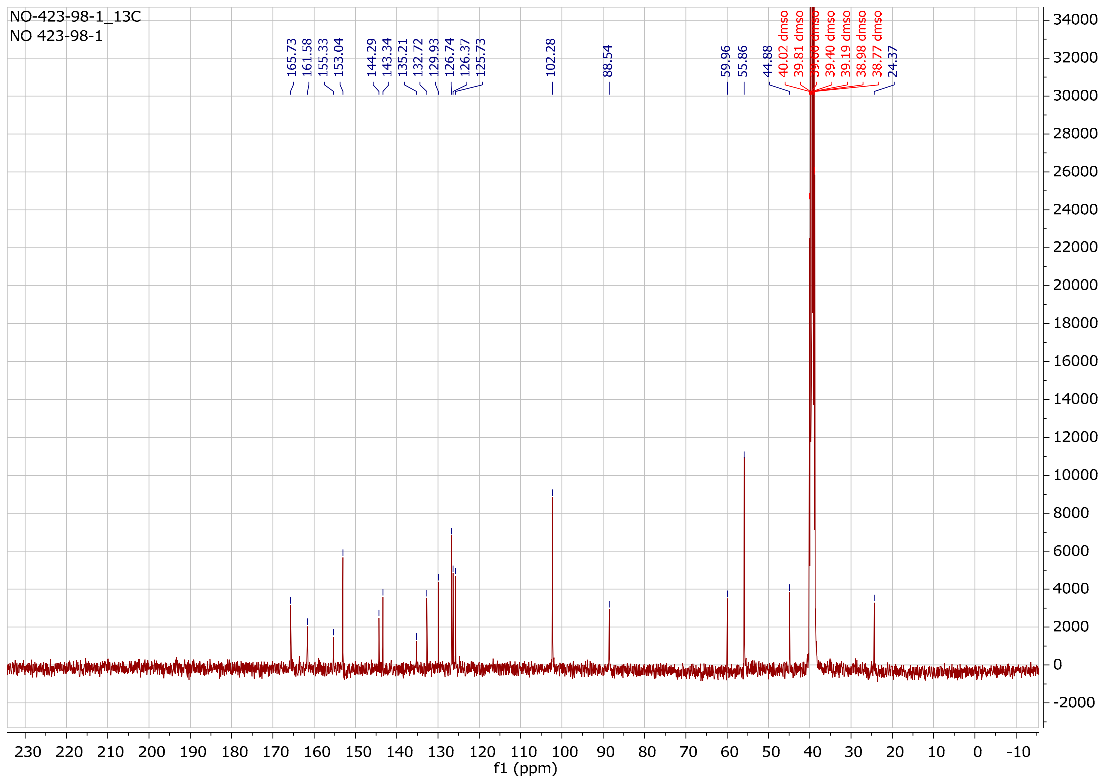

^{1}H-NMR and ^{13}C-NMR spectra of compound **7o**

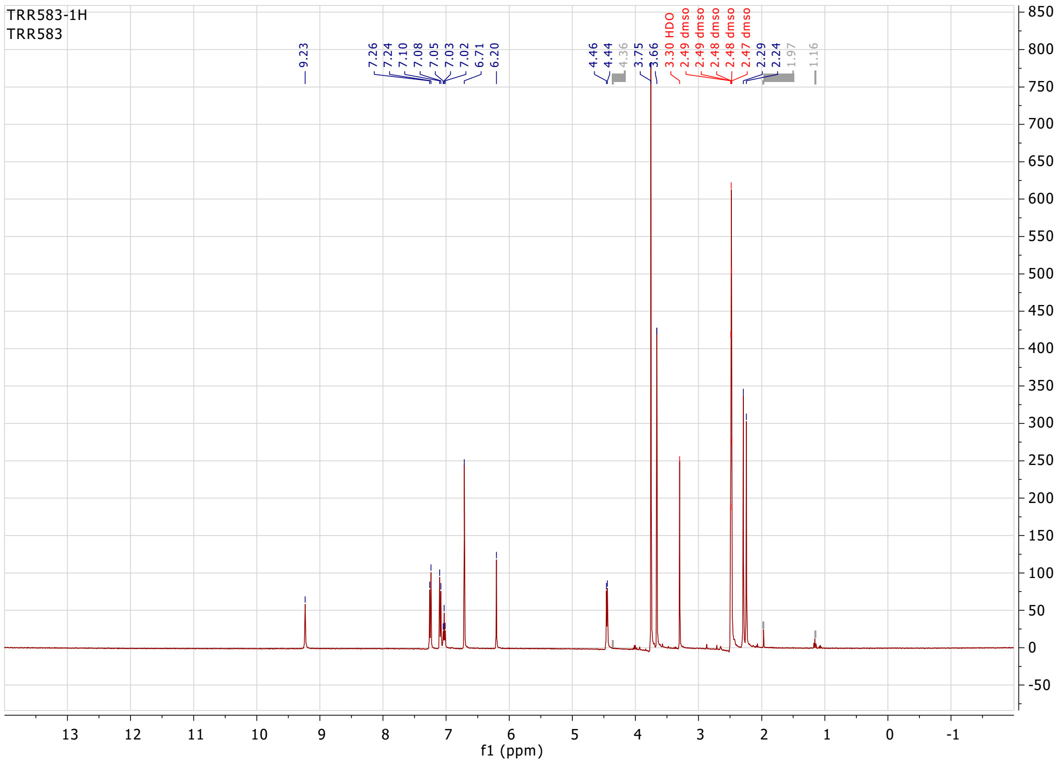

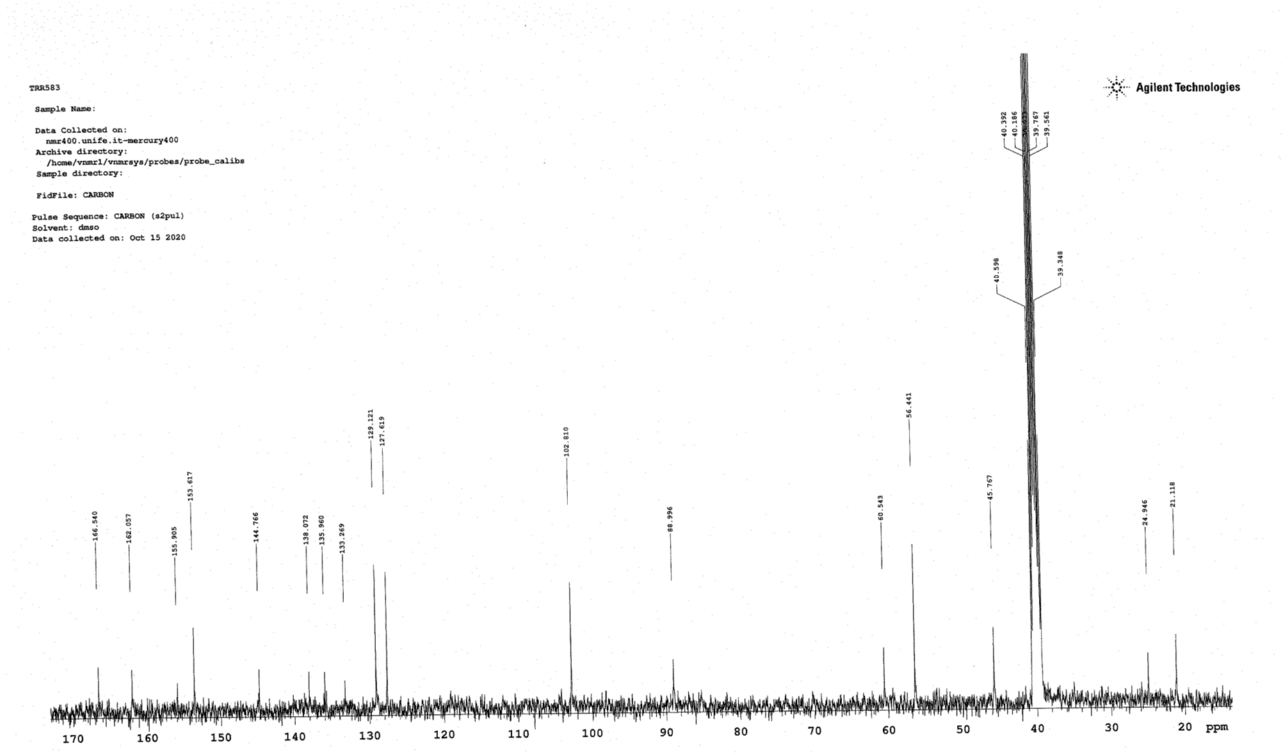

^{1}H-NMR and ^{13}C-NMR spectra of compound **7p**

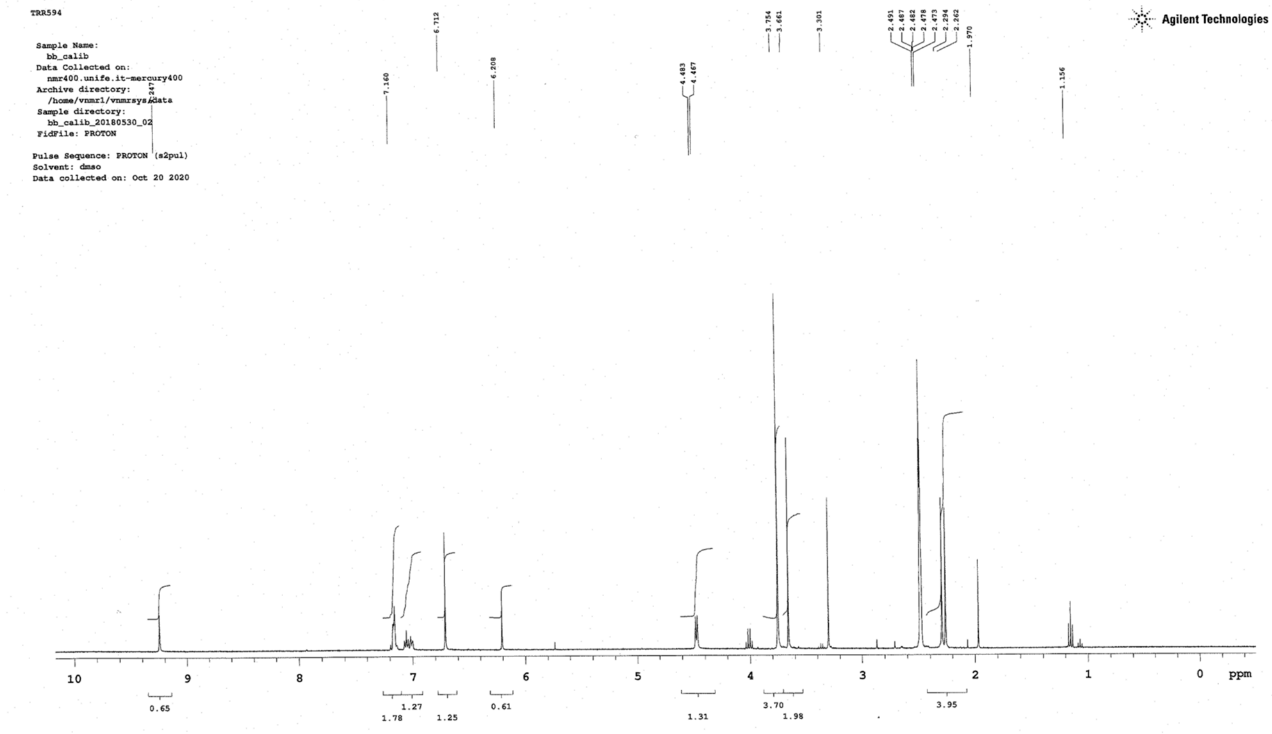

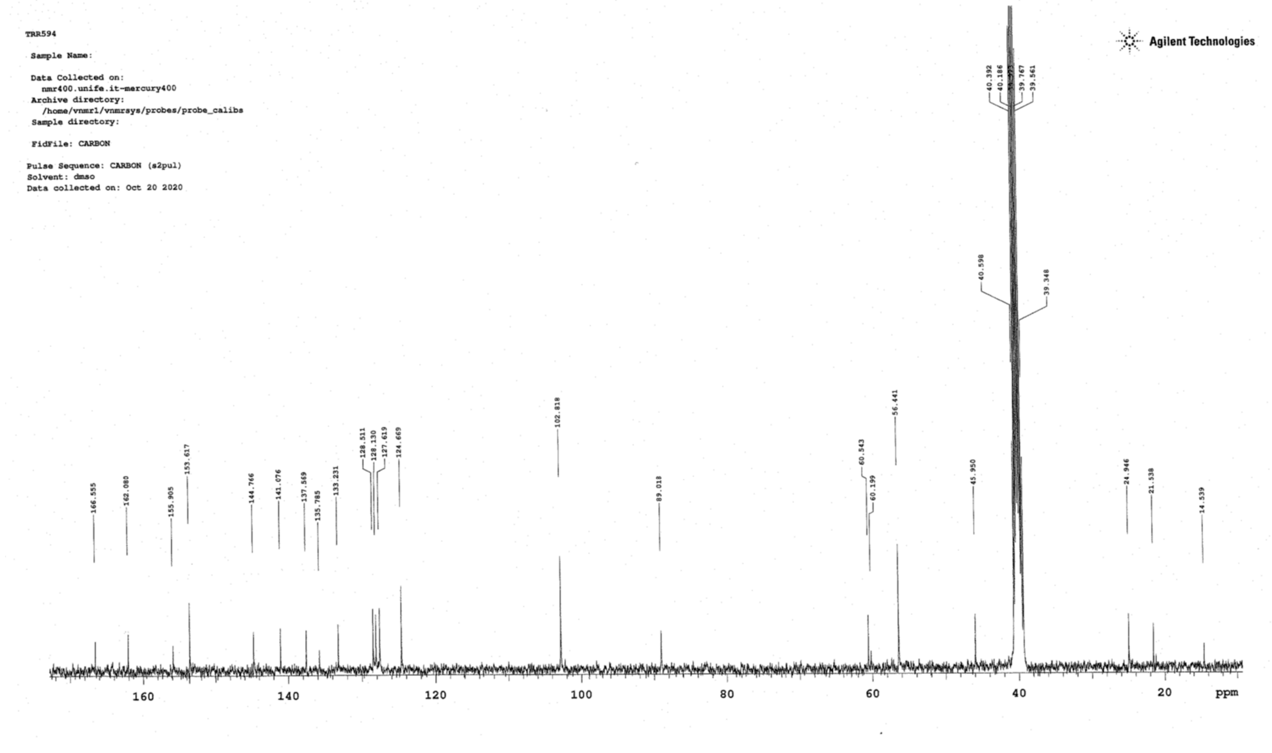

^{1}H-NMR and ^{13}C-NMR spectra of compound **7q**

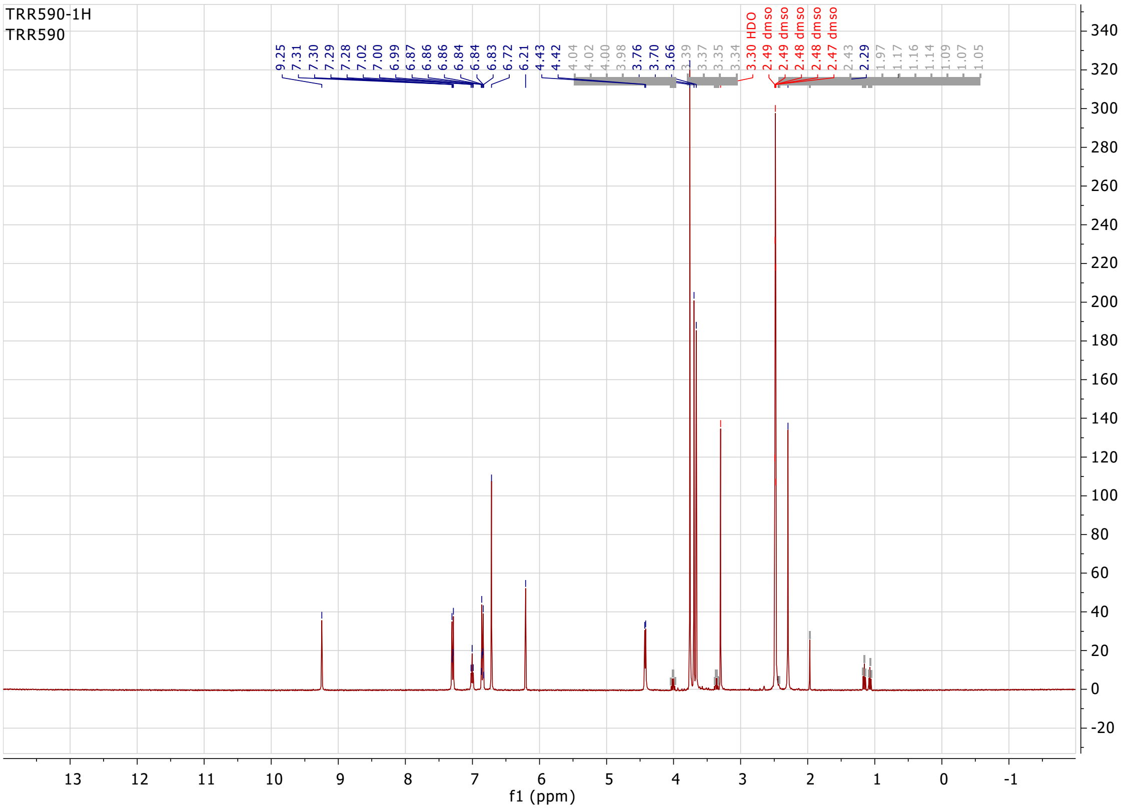

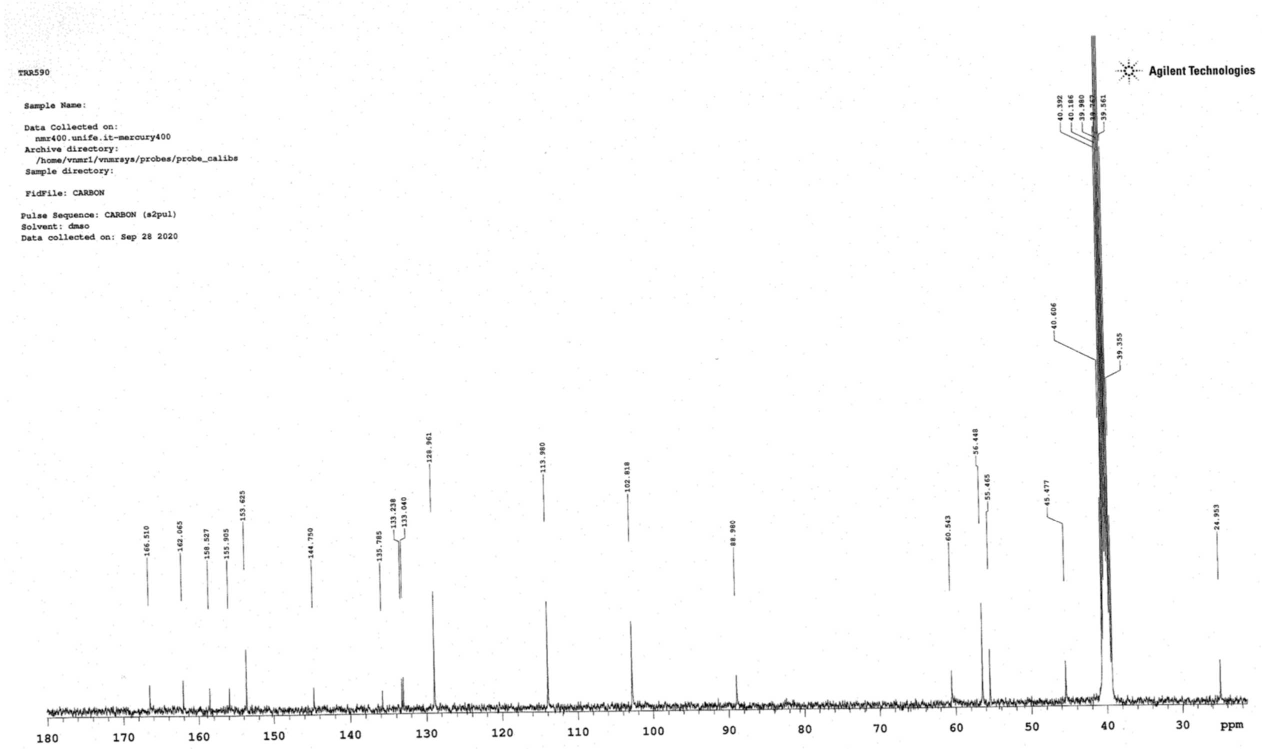

^{1}H-NMR and ^{13}C-NMR spectra of compound **7s**

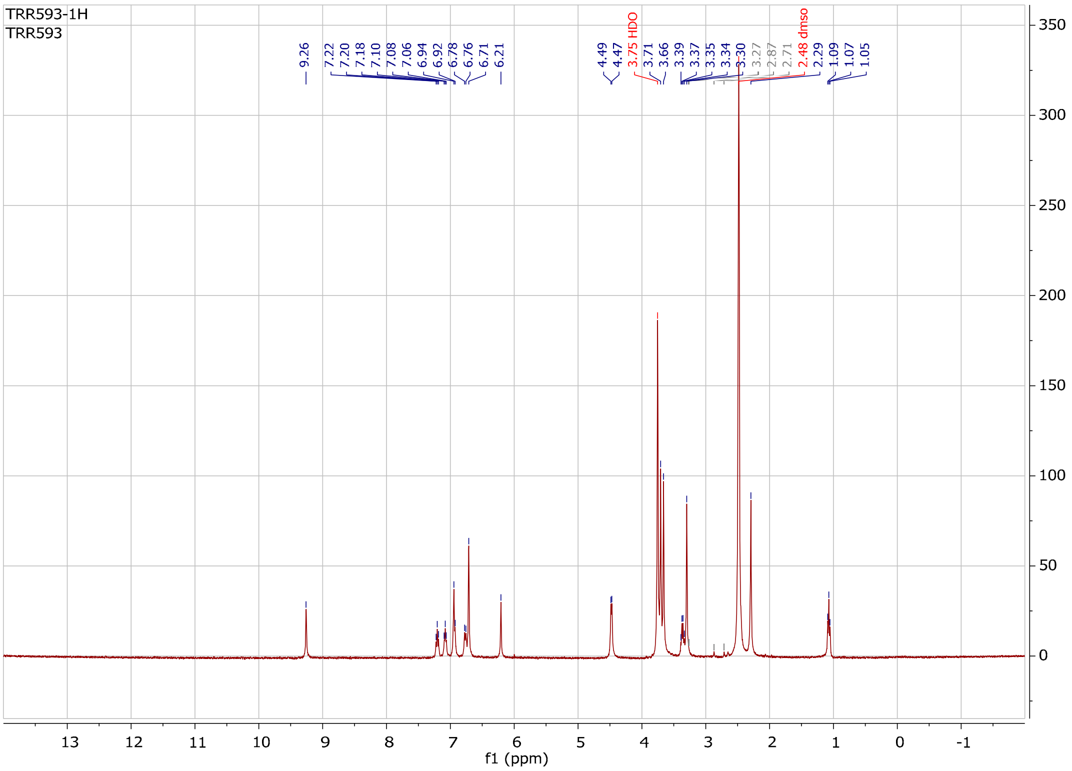

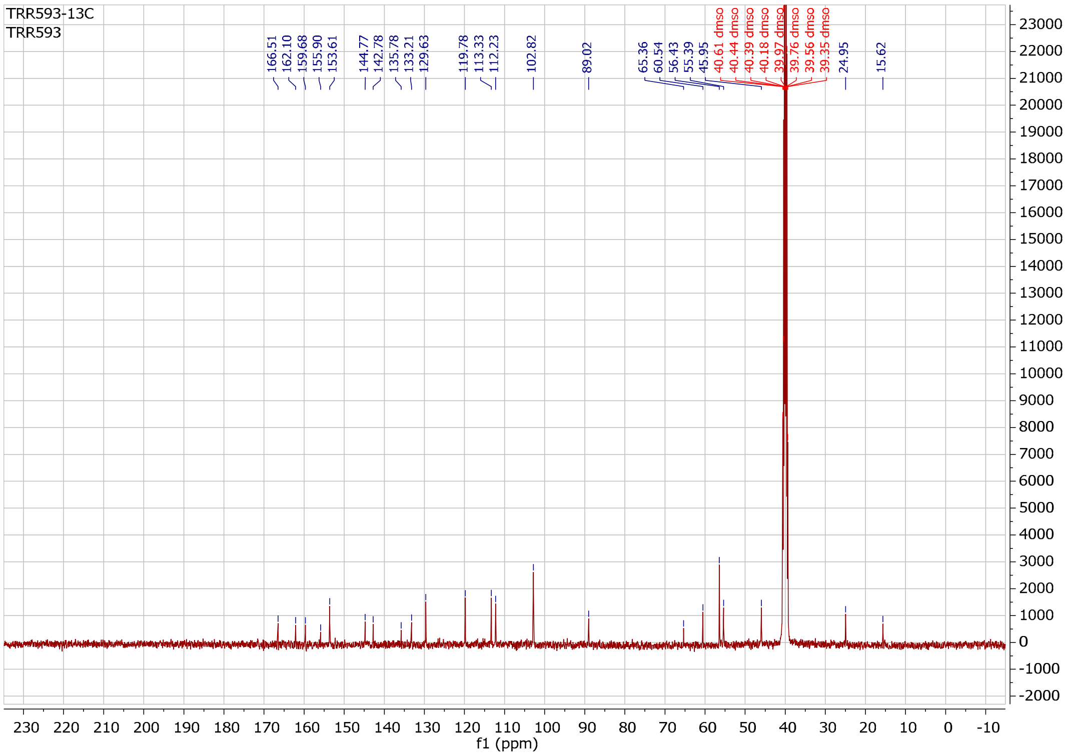

^{1}H-NMR and ^{13}C-NMR spectra of compound **7t**

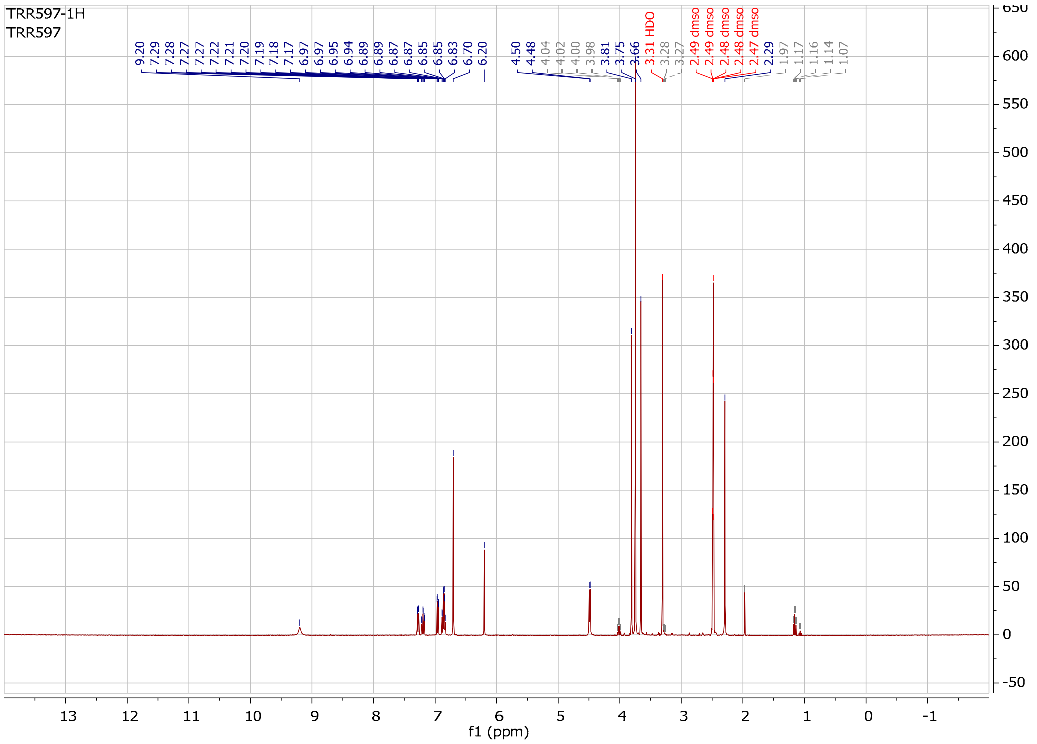

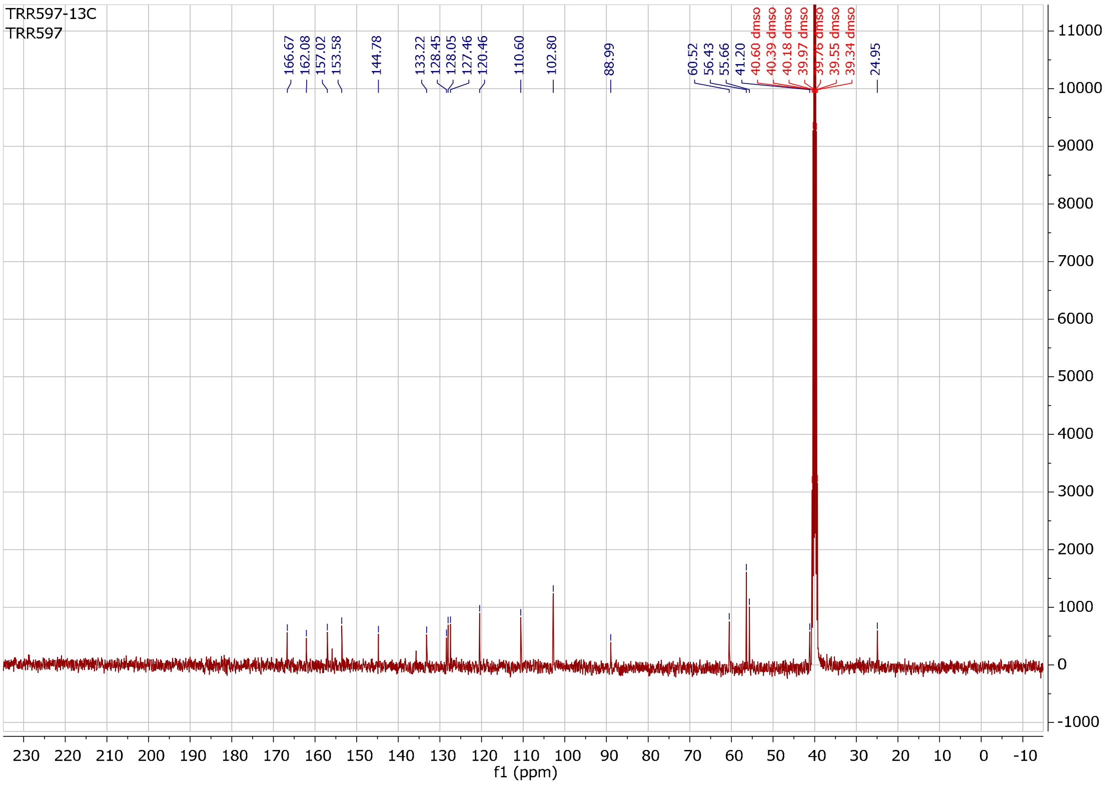

^{1}H-NMR and ^{13}C-NMR spectra of compound **7u**

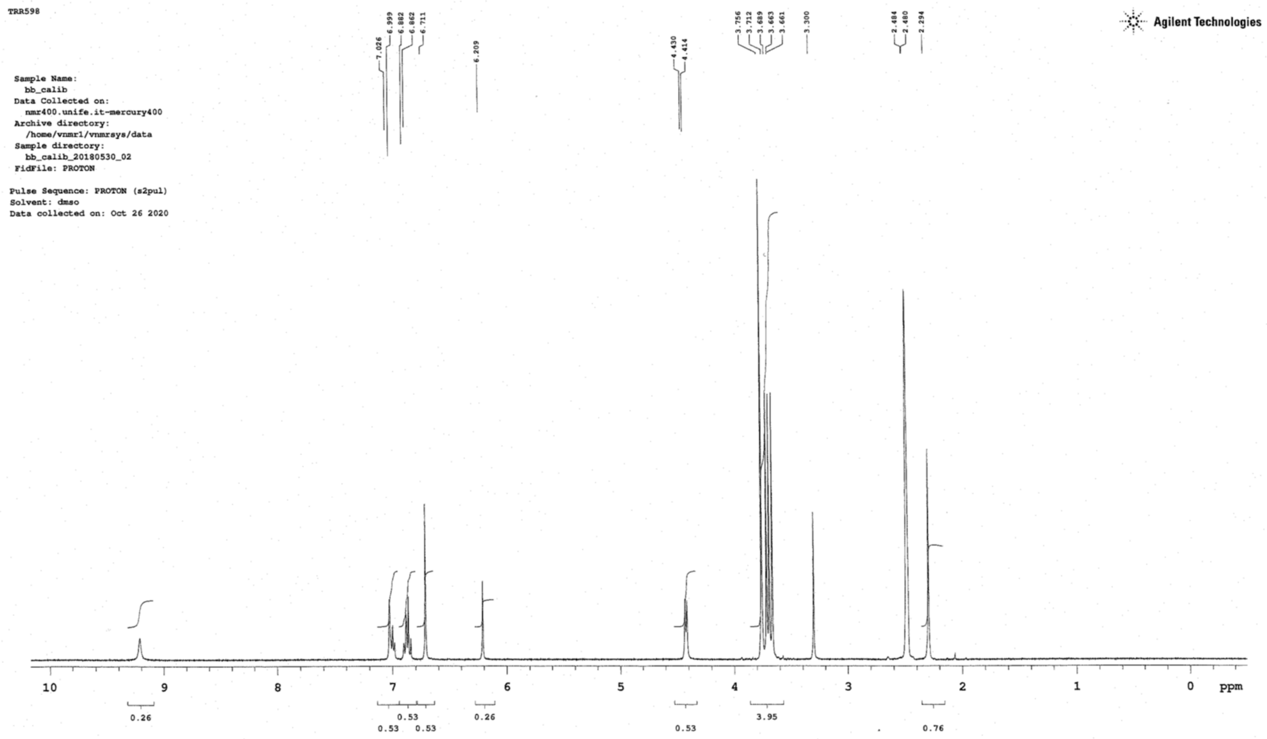

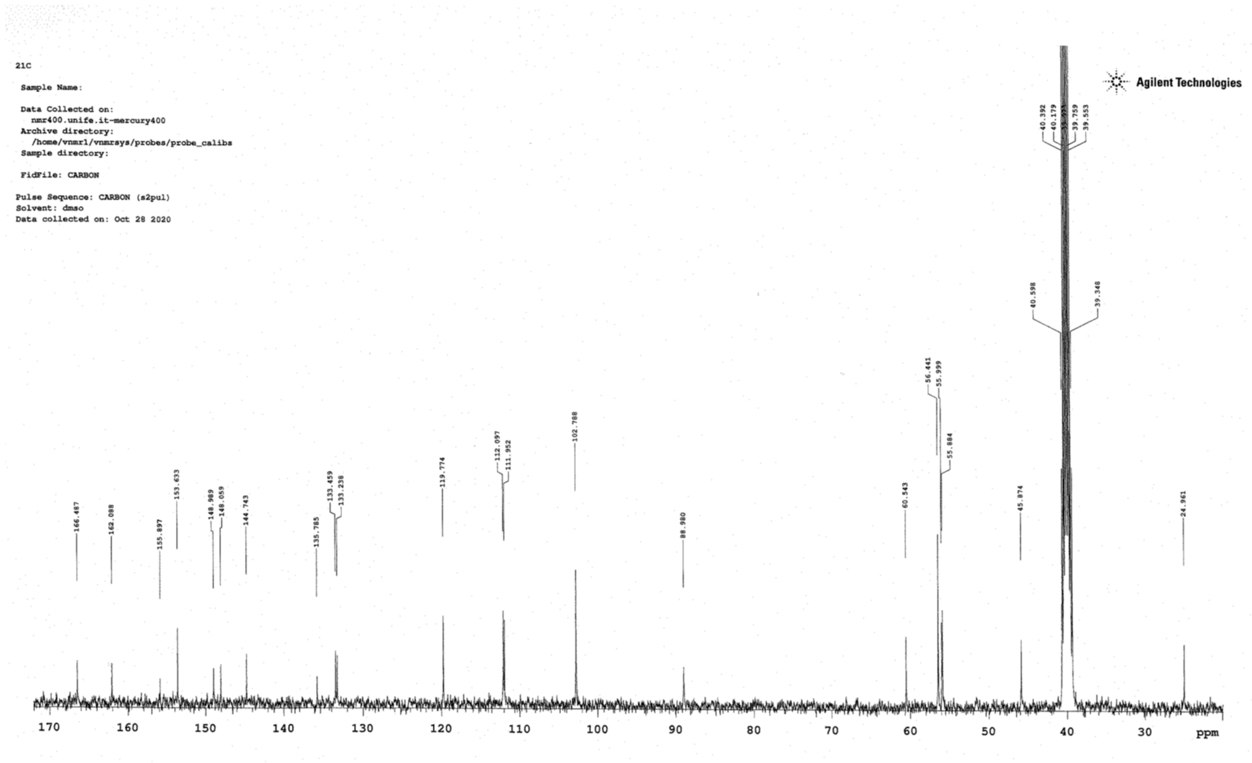

^{1}H-NMR and ^{13}C-NMR spectra of compound **7v**

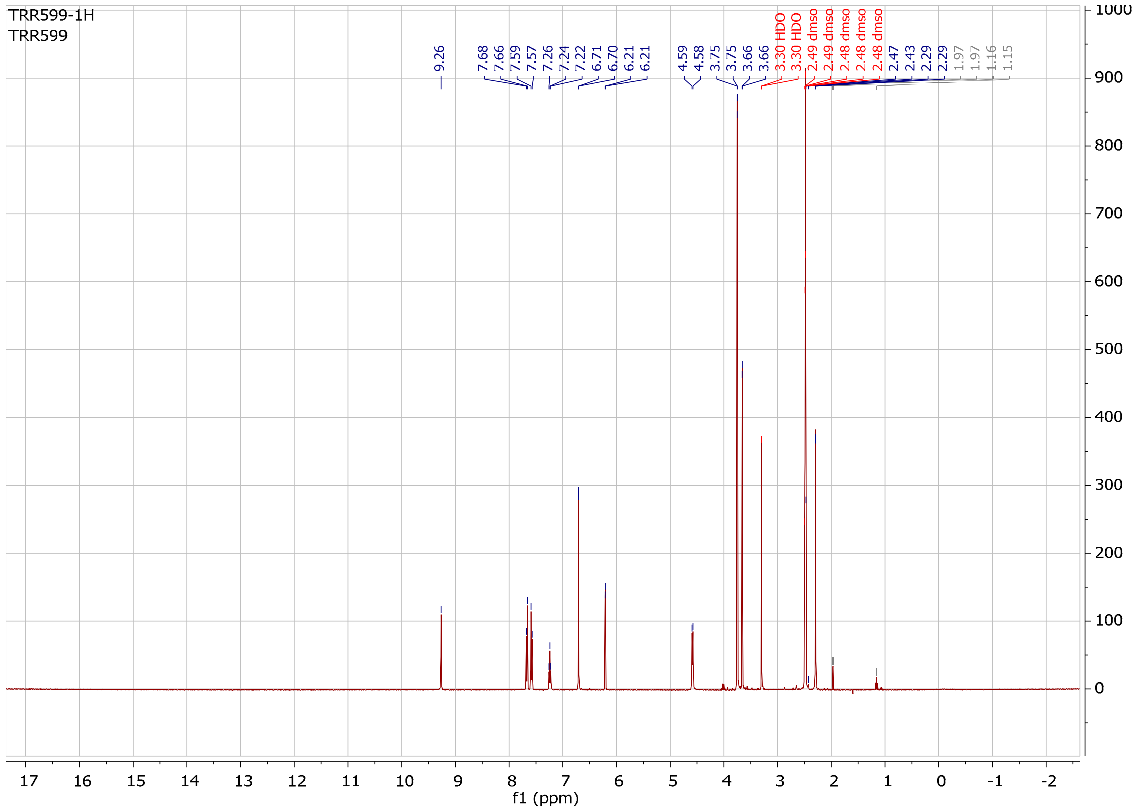

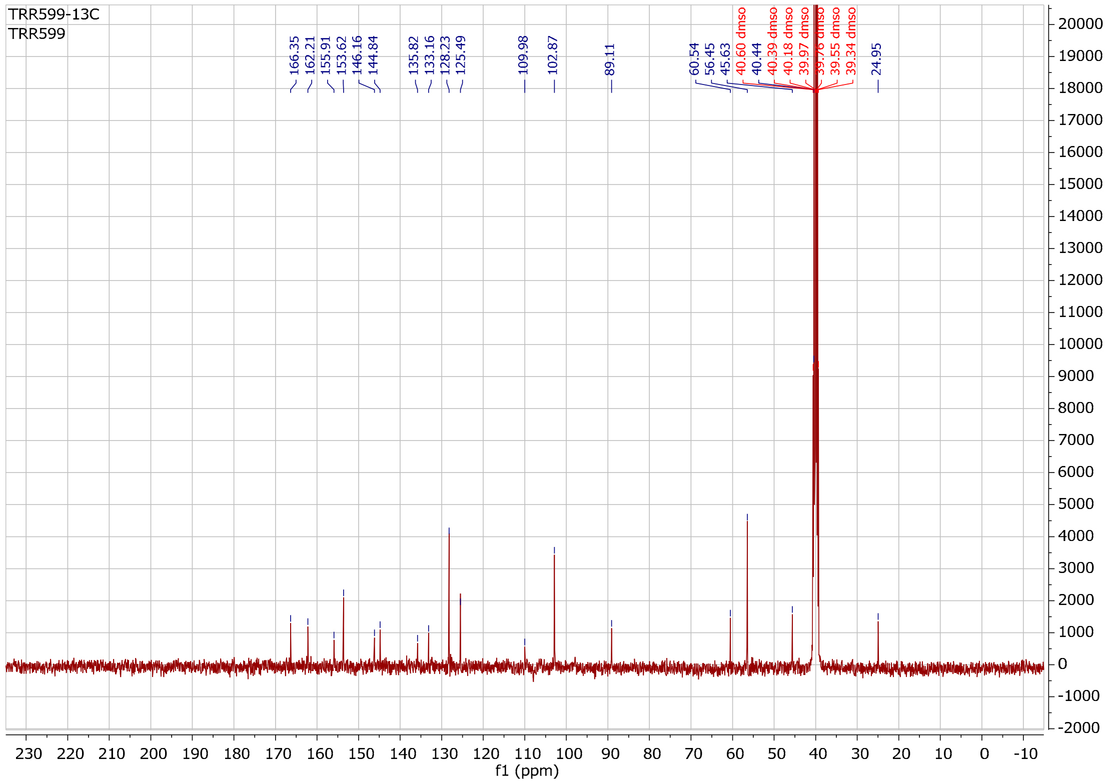

^{1}H-NMR and ^{13}C-NMR spectra of compound **7w**

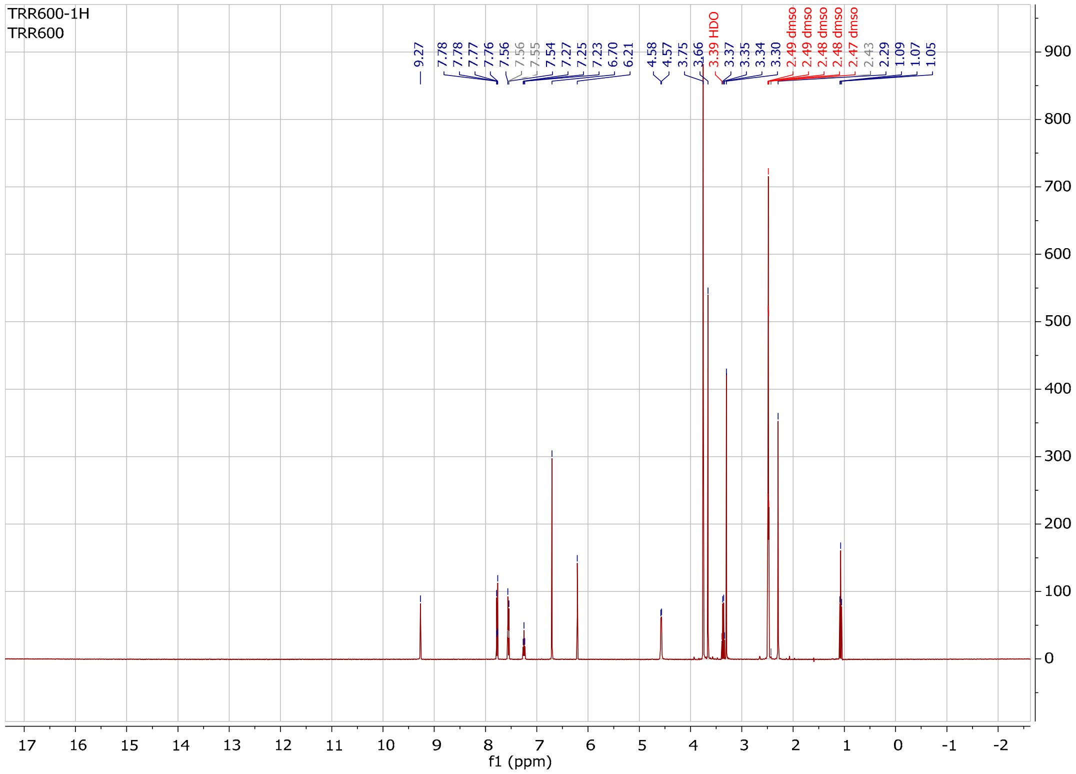

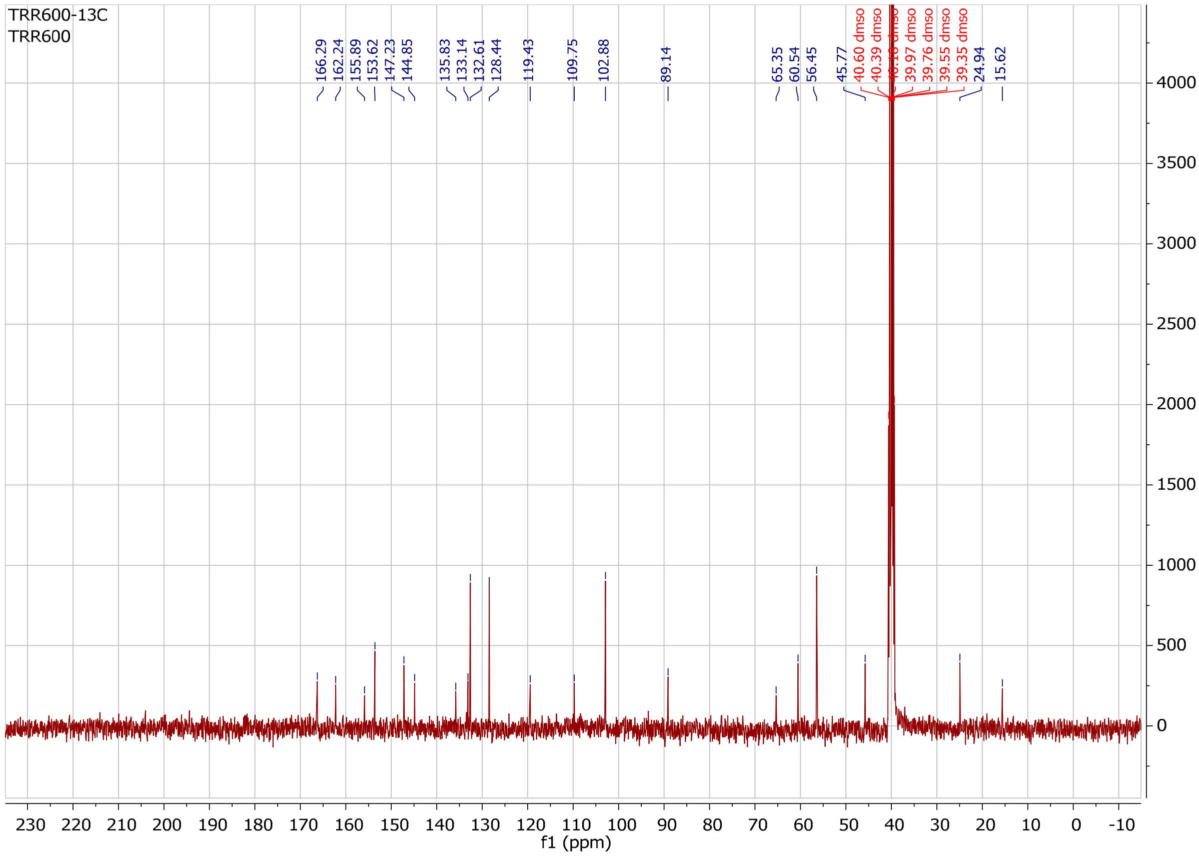

^{1}H-NMR and ^{13}C-NMR spectra of compound **7x**

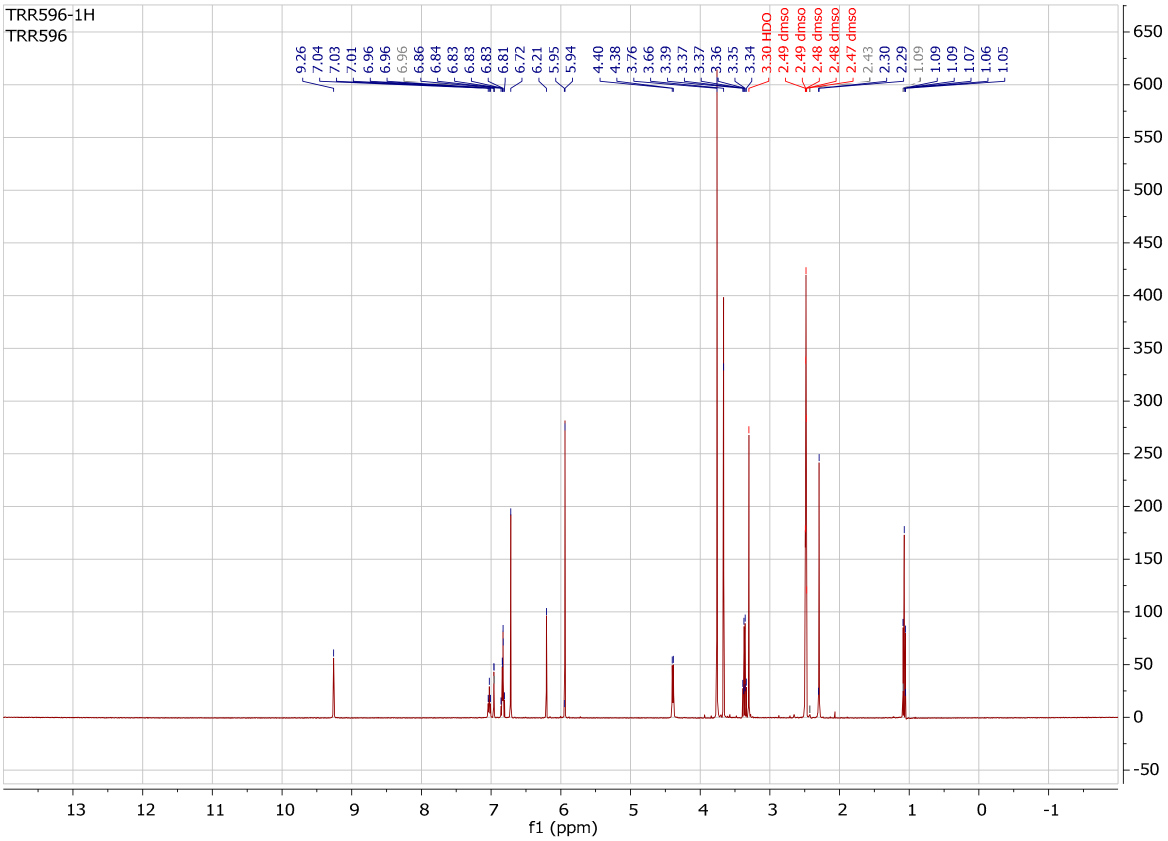

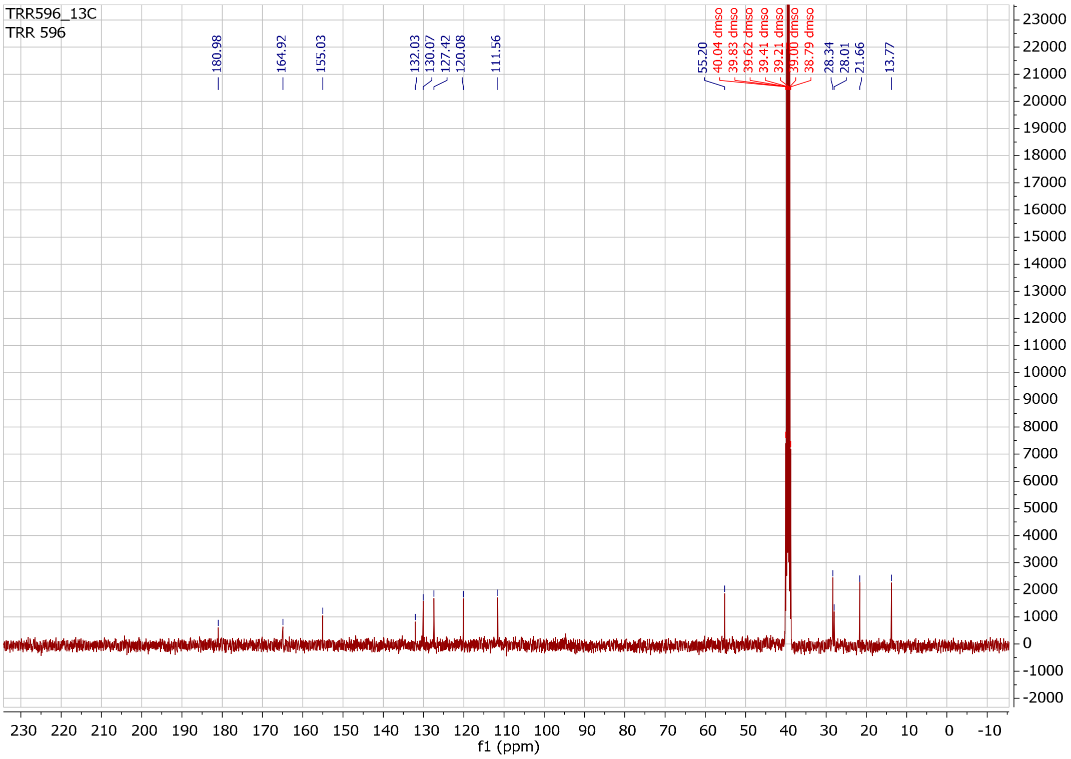

^{1}H-NMR and ^{13}C-NMR spectra of compound **7y**

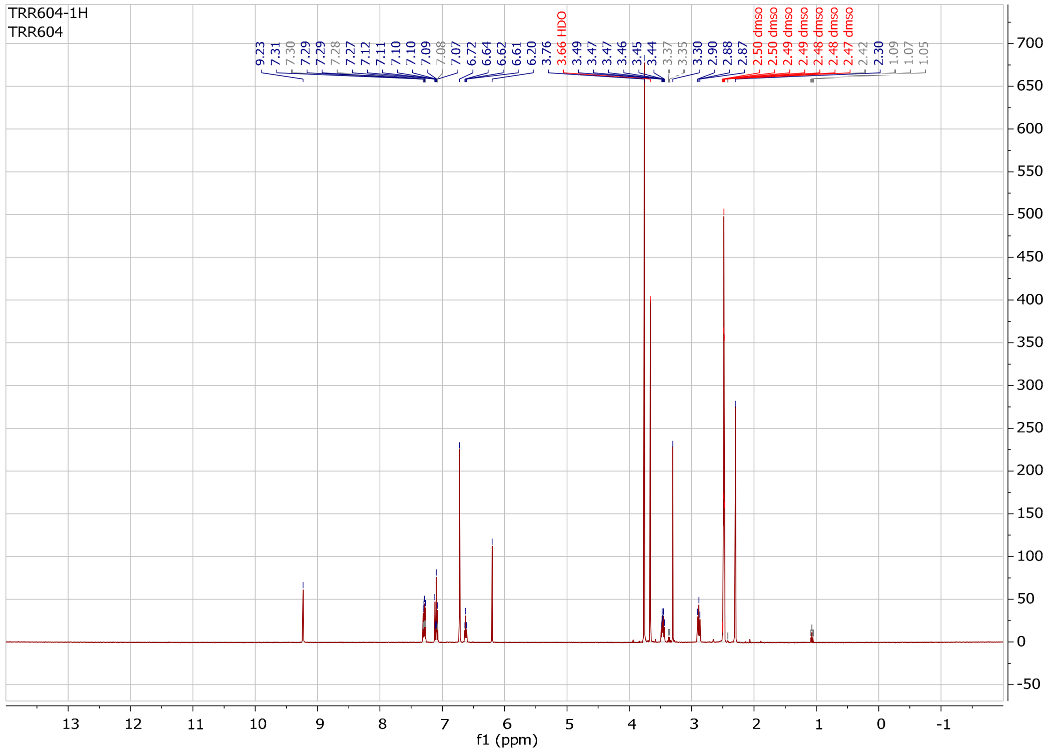

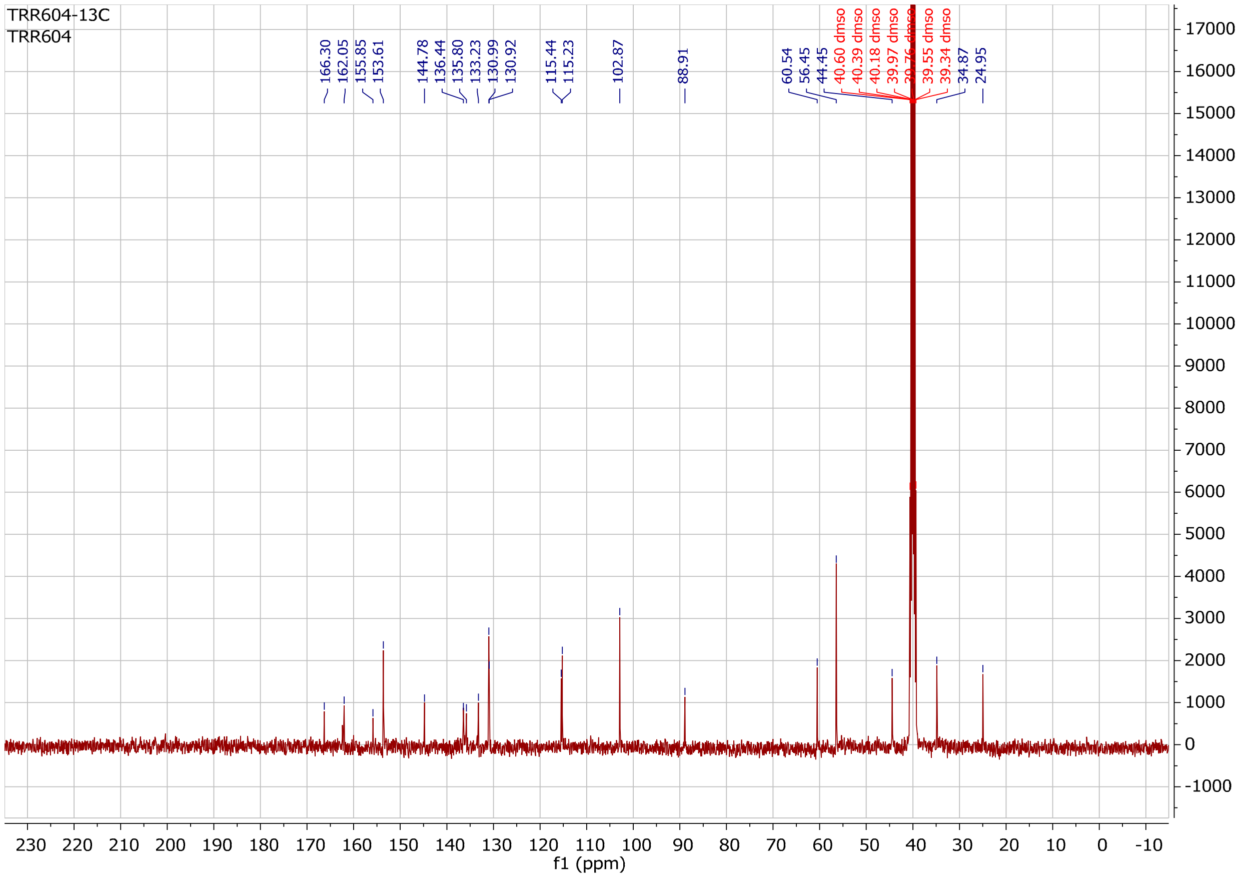

^{1}H-NMR and ^{13}C-NMR spectra of compound **7aa**

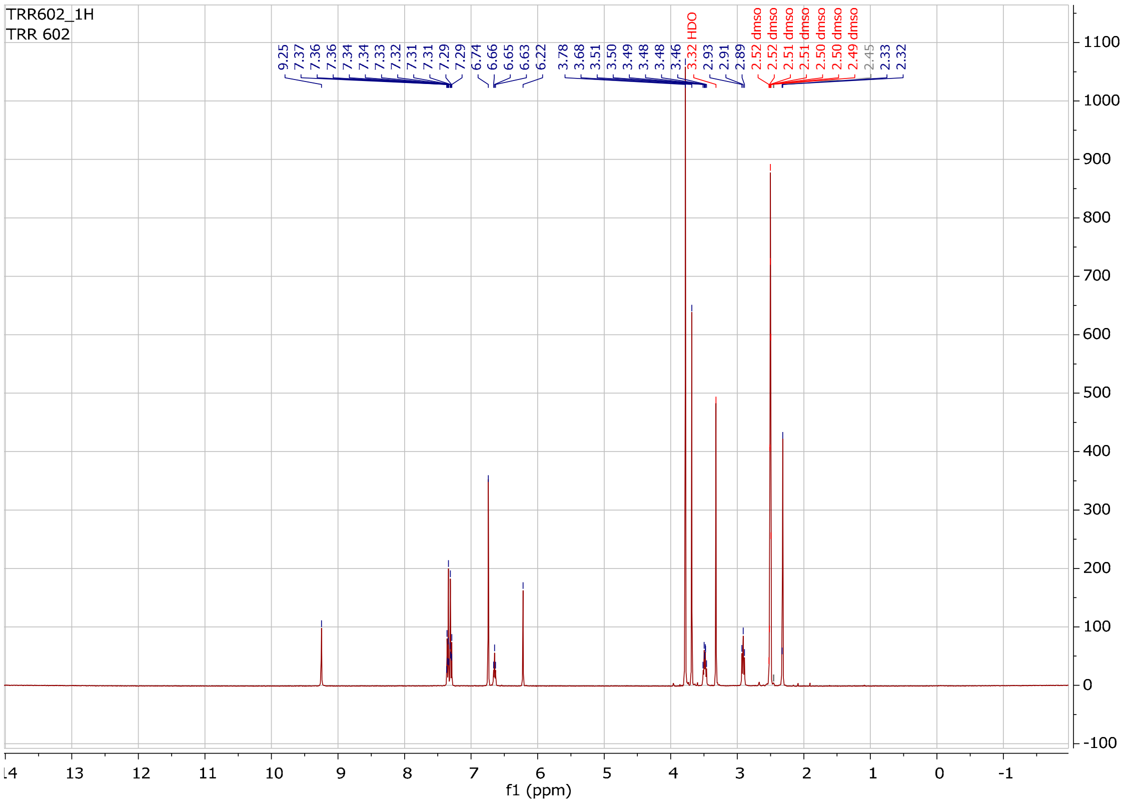

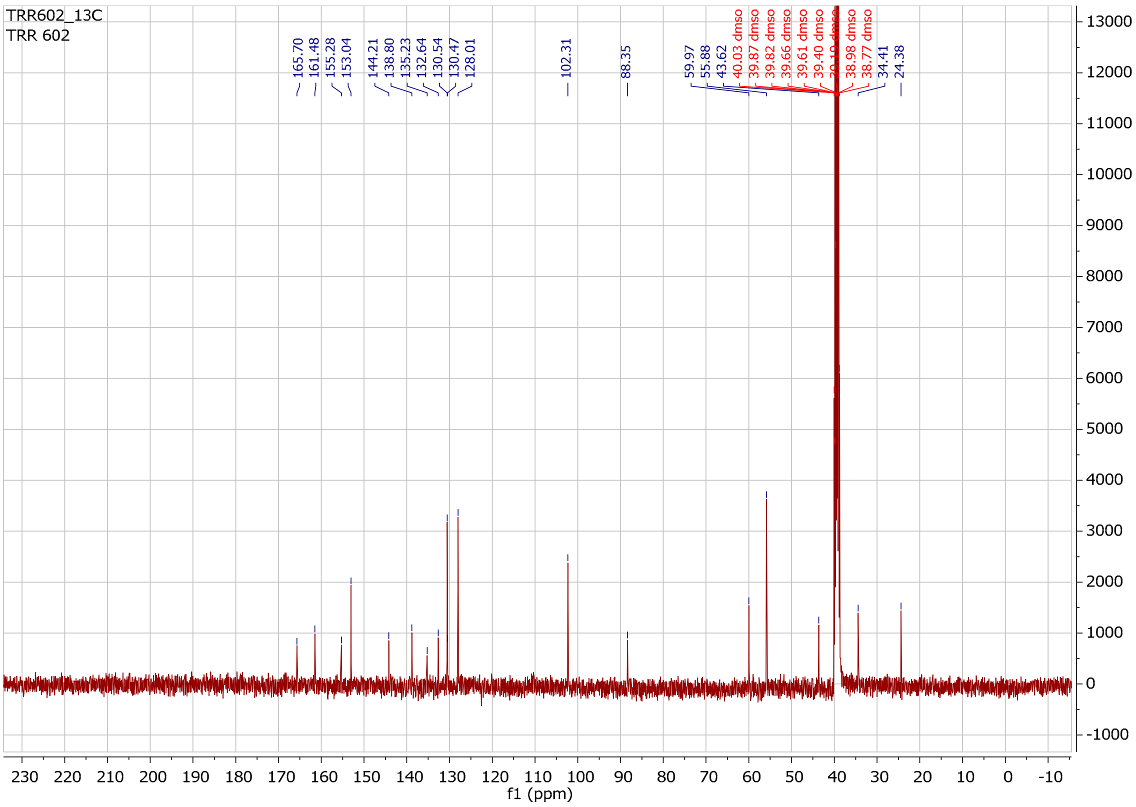

^{1}H-NMR and ^{13}C-NMR spectra of compound **7ab**

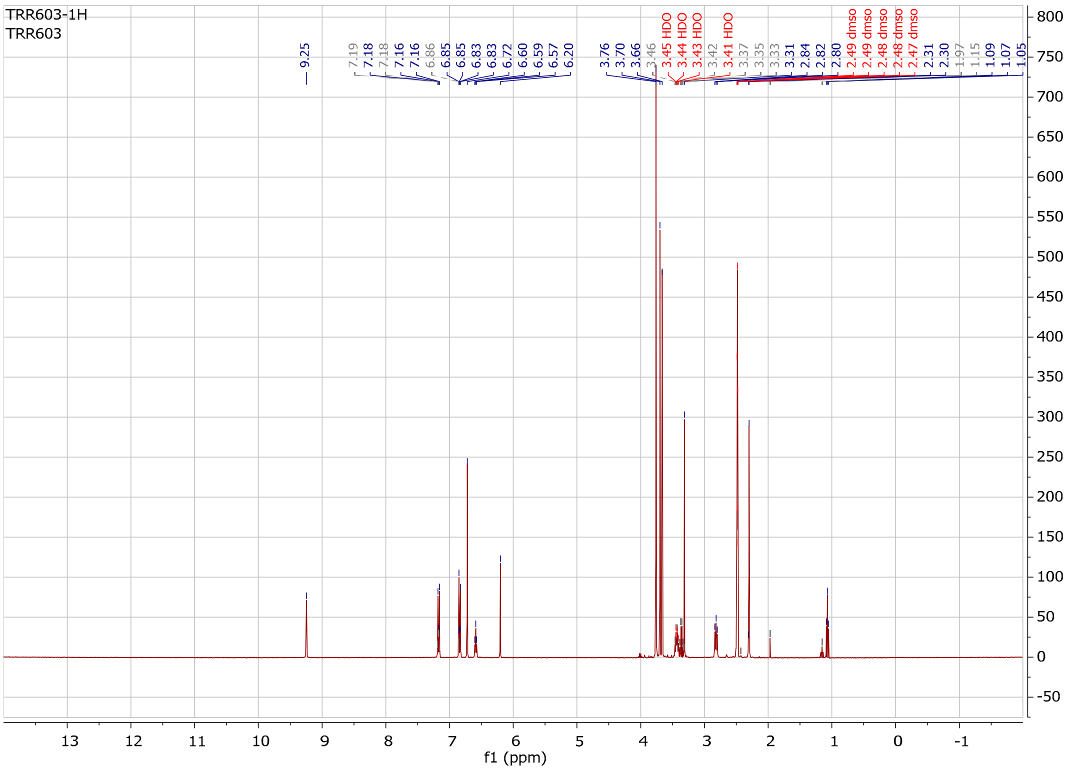

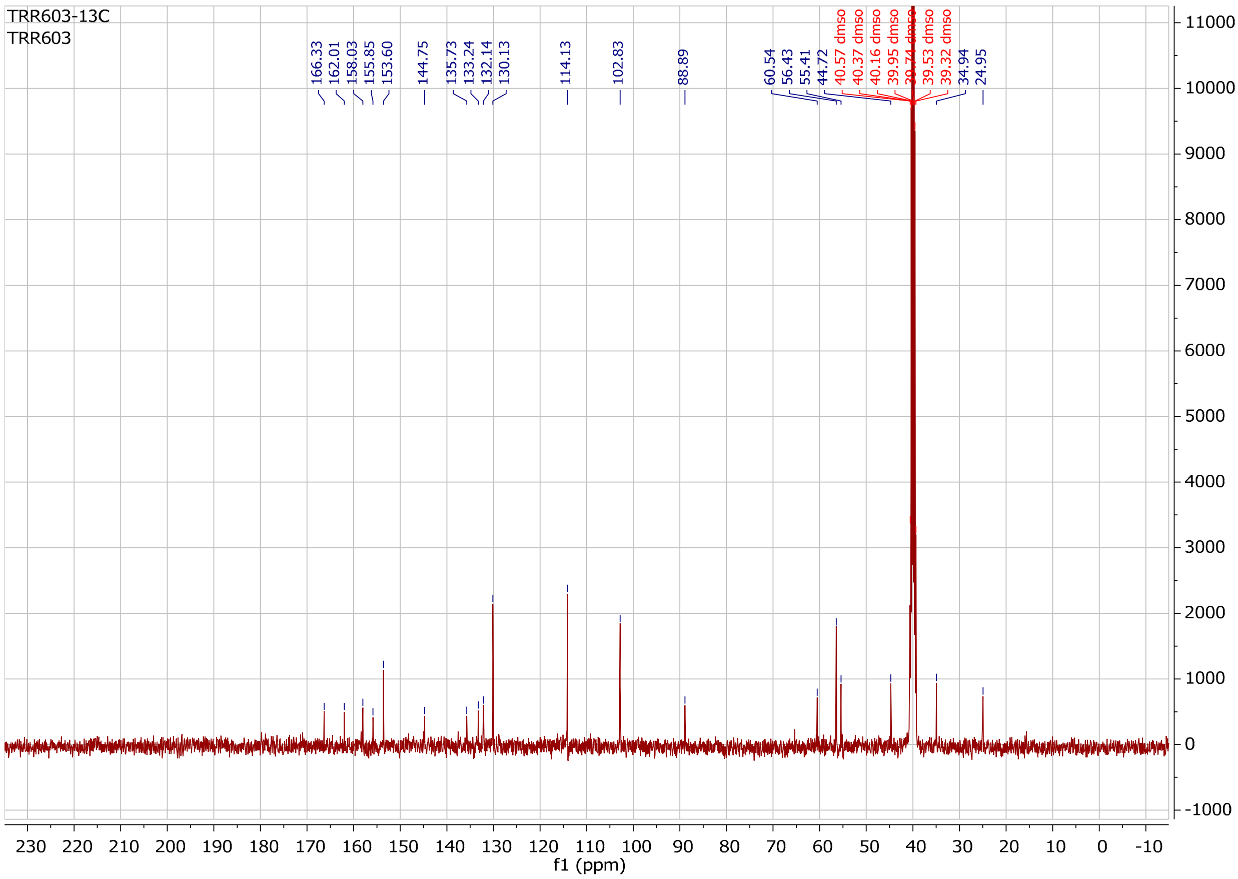

^{1}H-NMR and ^{13}C-NMR spectra of compound **7ac**

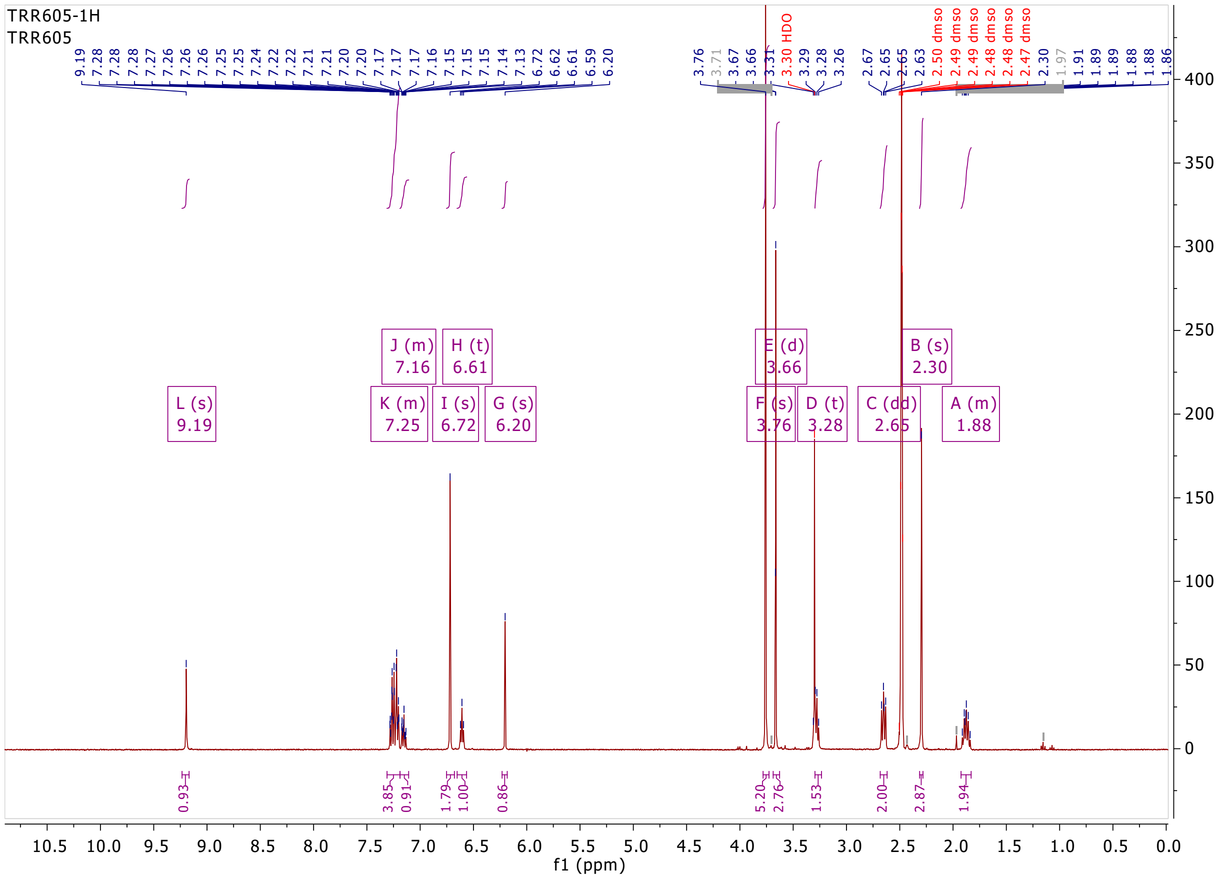

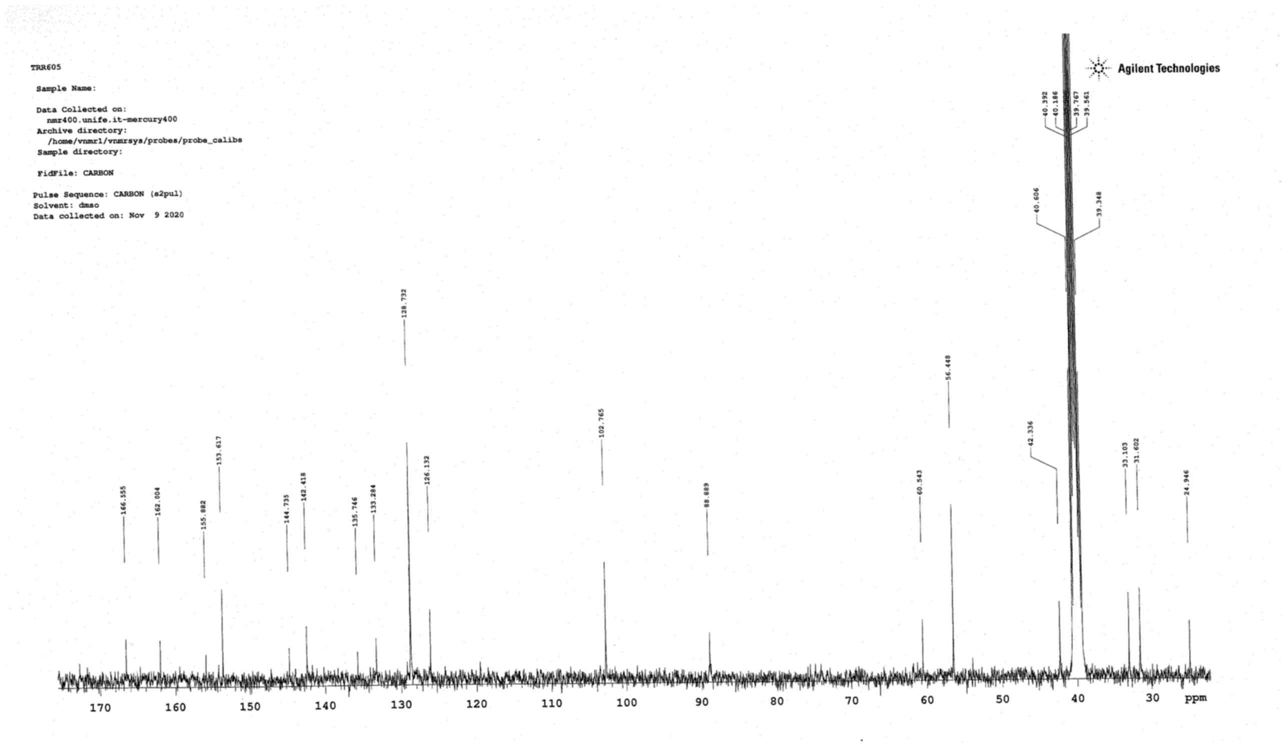

^{1}H-NMR and ^{13}C-NMR spectra of compound **7ad**

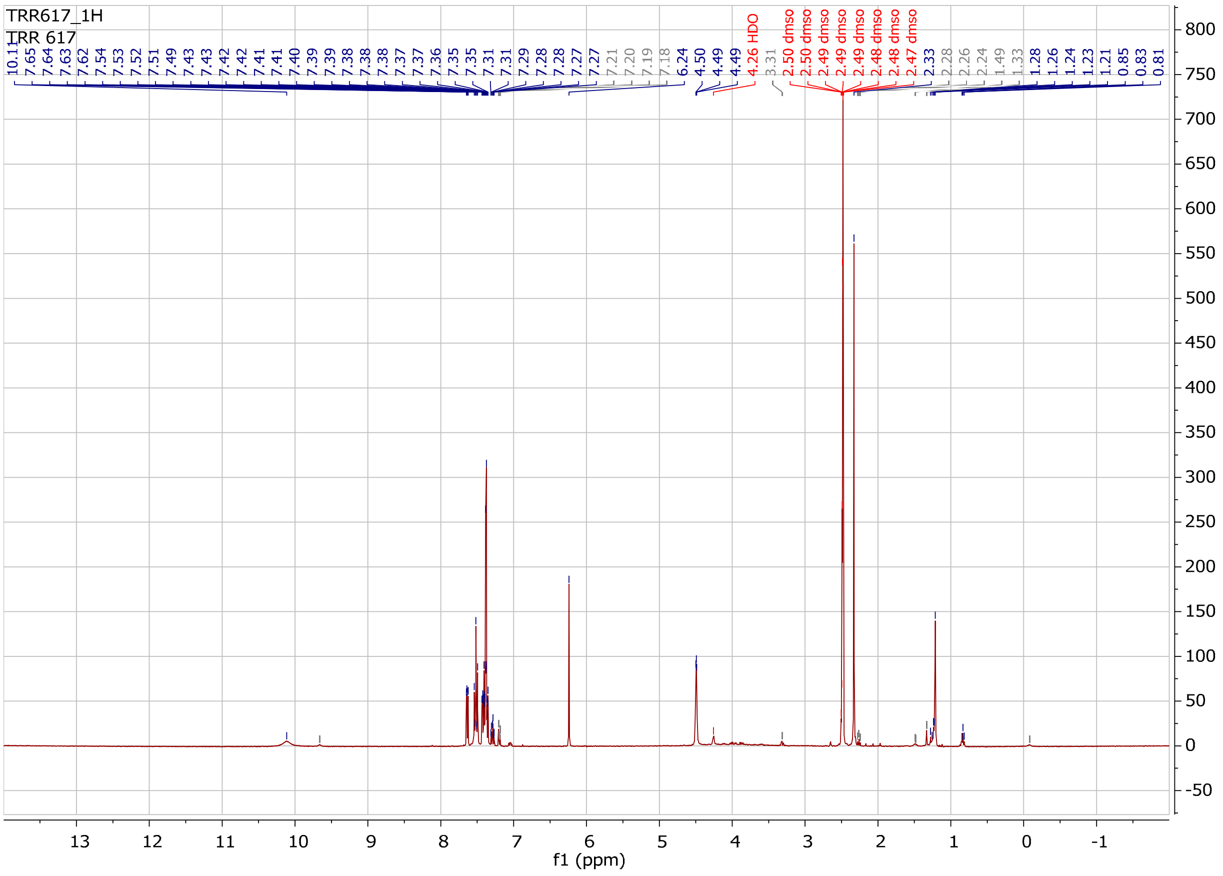

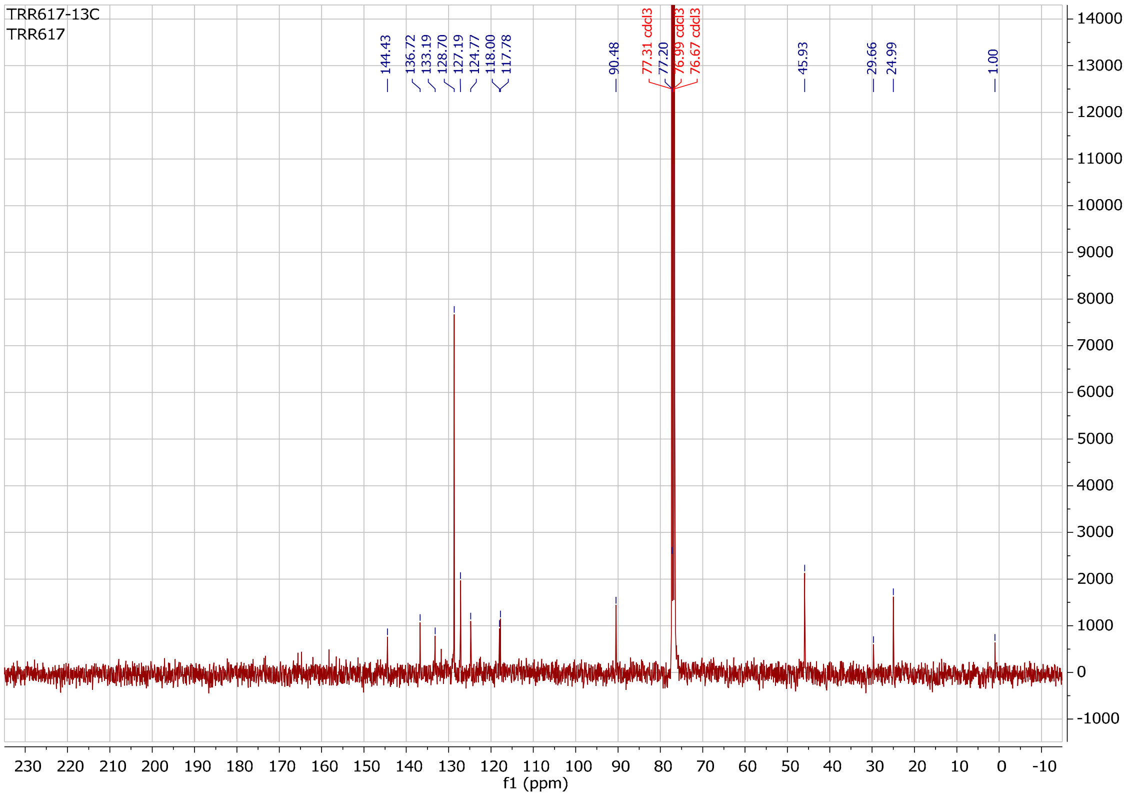

^{1}H-NMR and ^{13}C-NMR spectra of compound **8a**

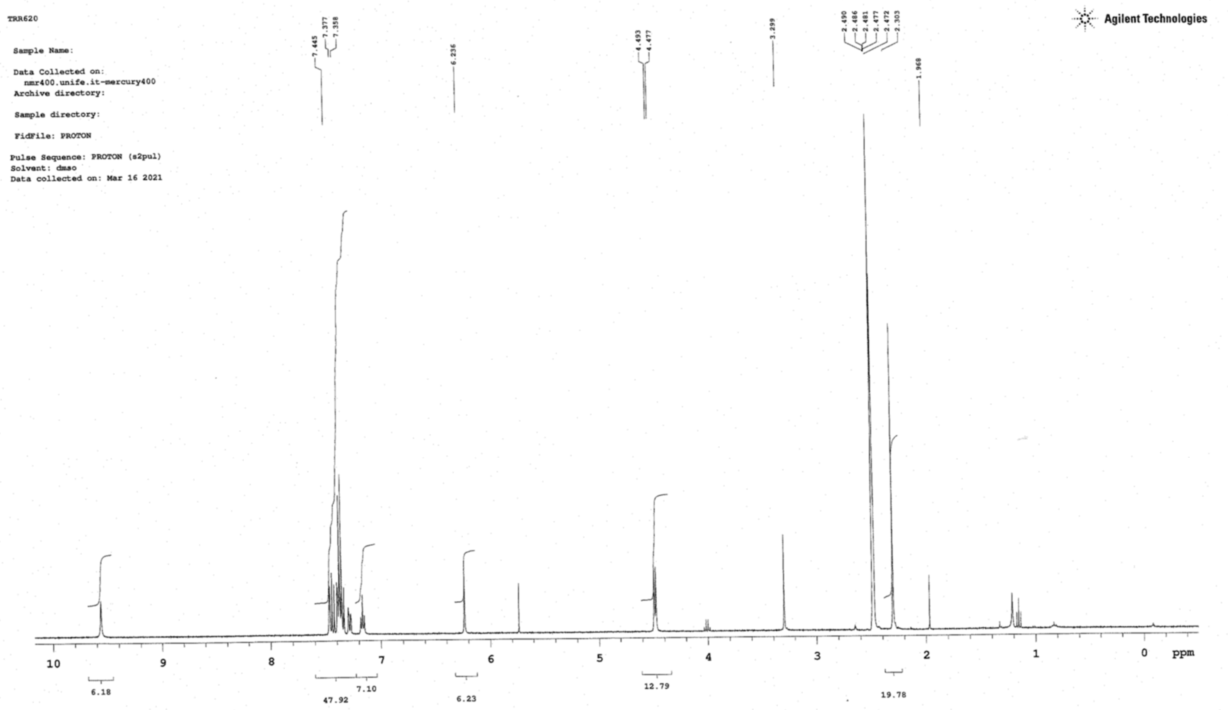

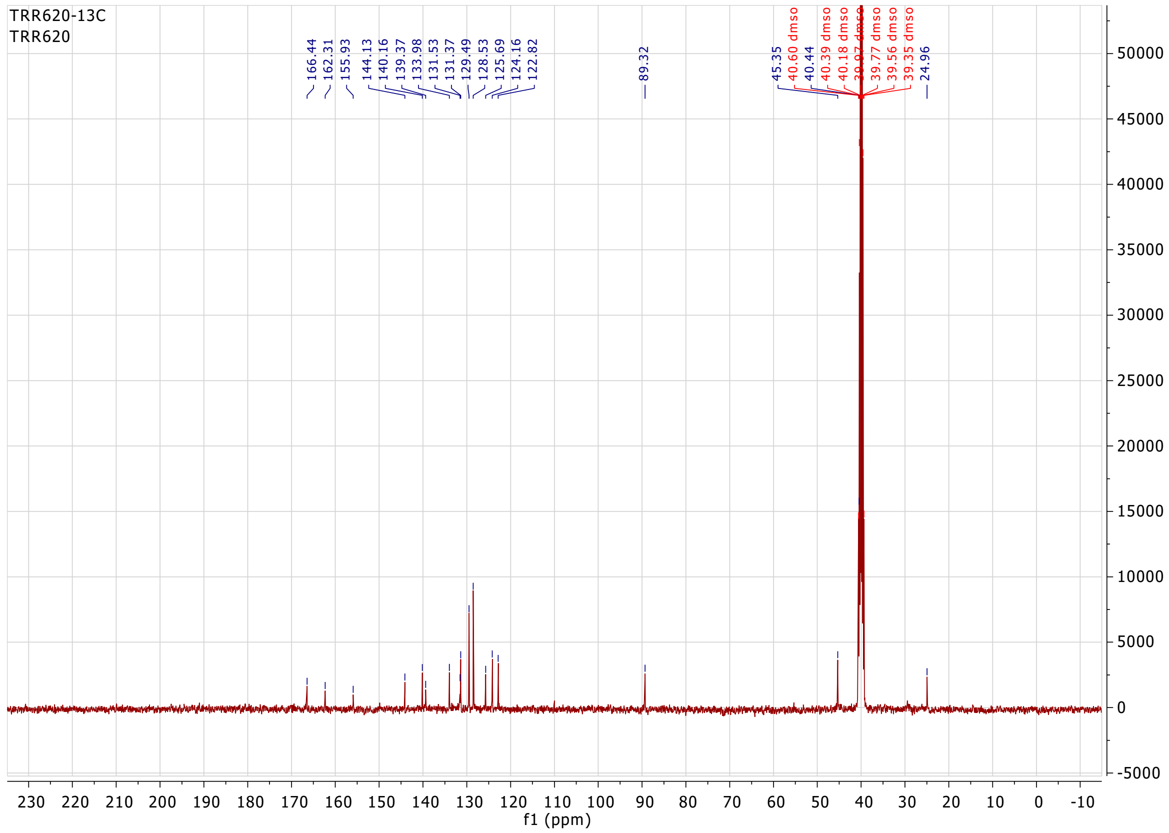

^{1}H-NMR and ^{13}C-NMR spectra of compound **8b**

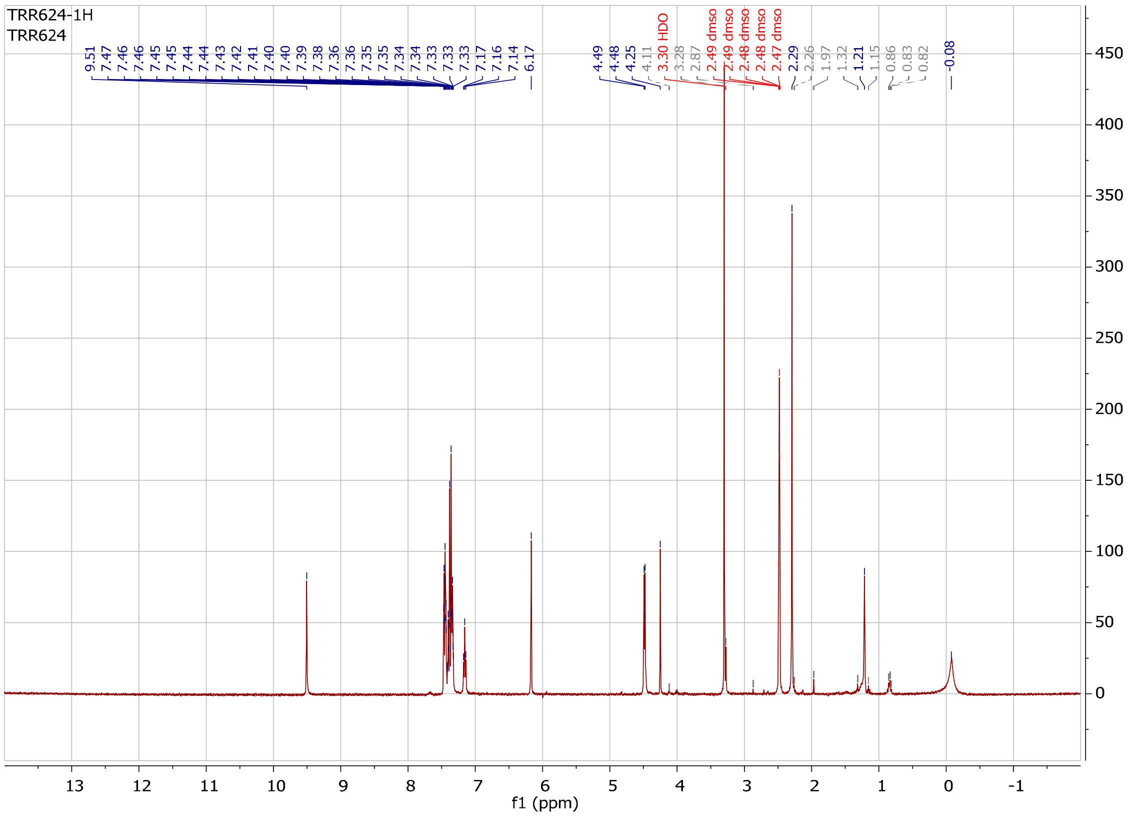

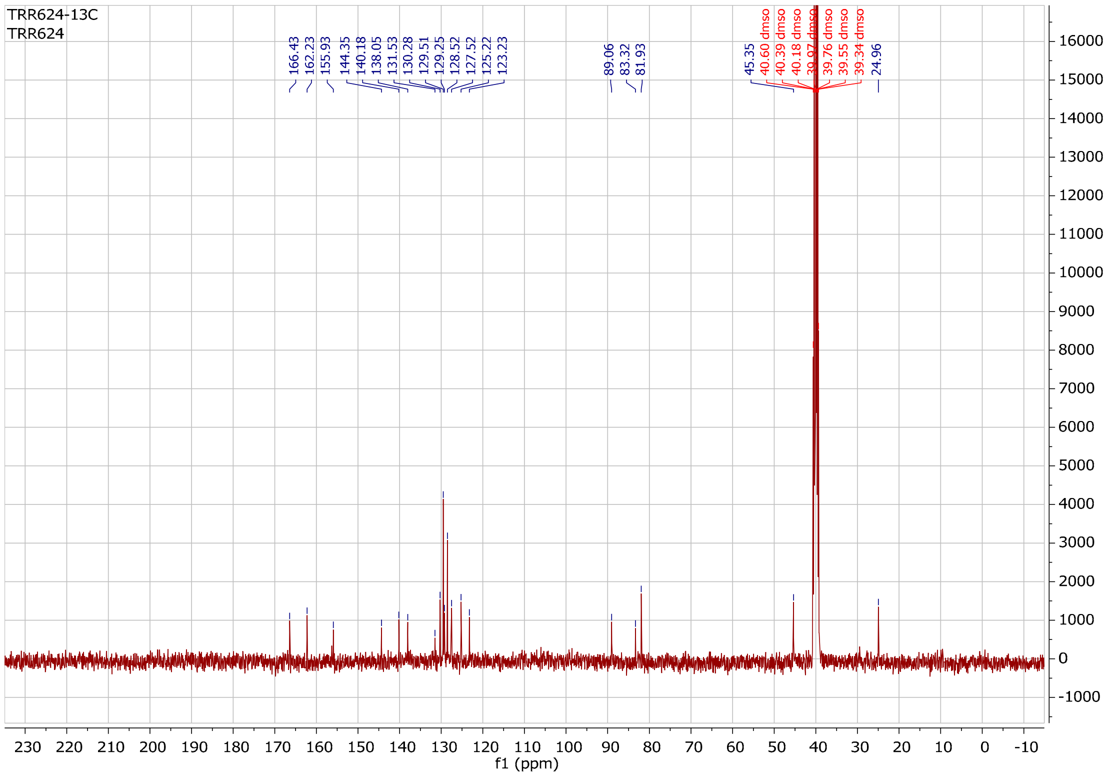

^{1}H-NMR and ^{13}C-NMR spectra of compound **8c**

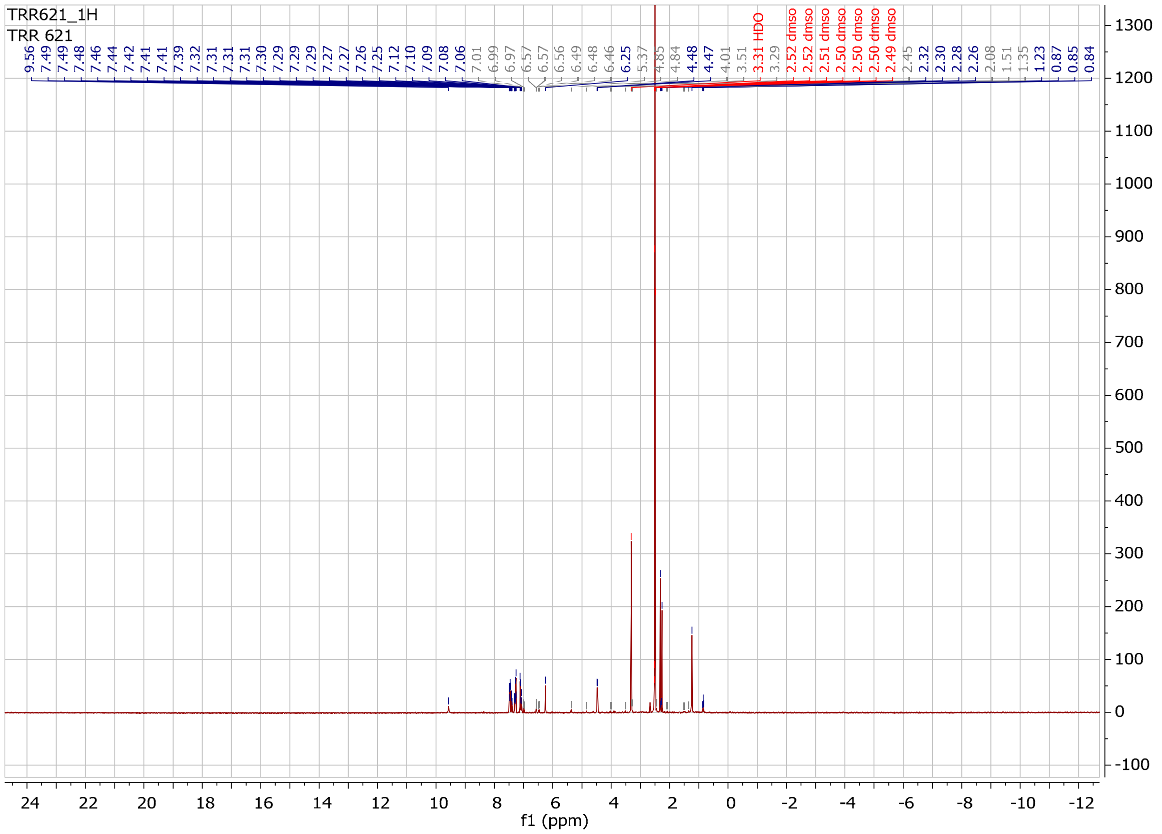

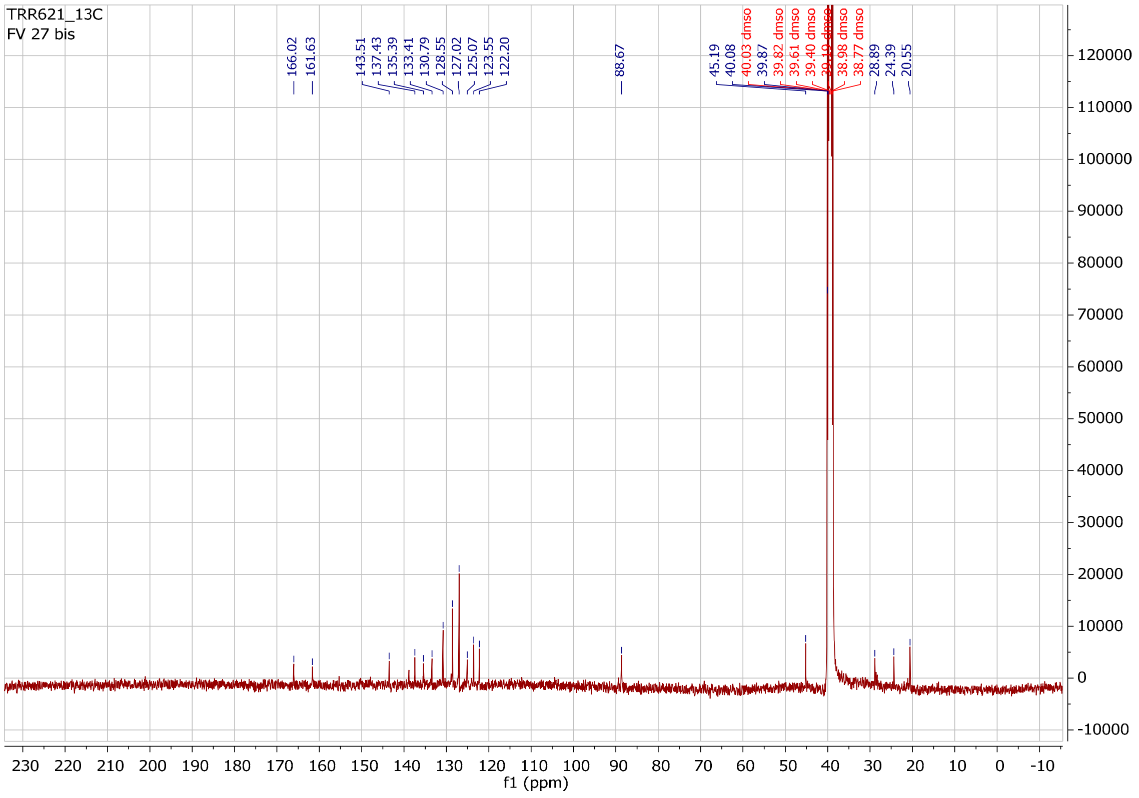

^{1}H-NMR and ^{13}C-NMR spectra of compound **8e**

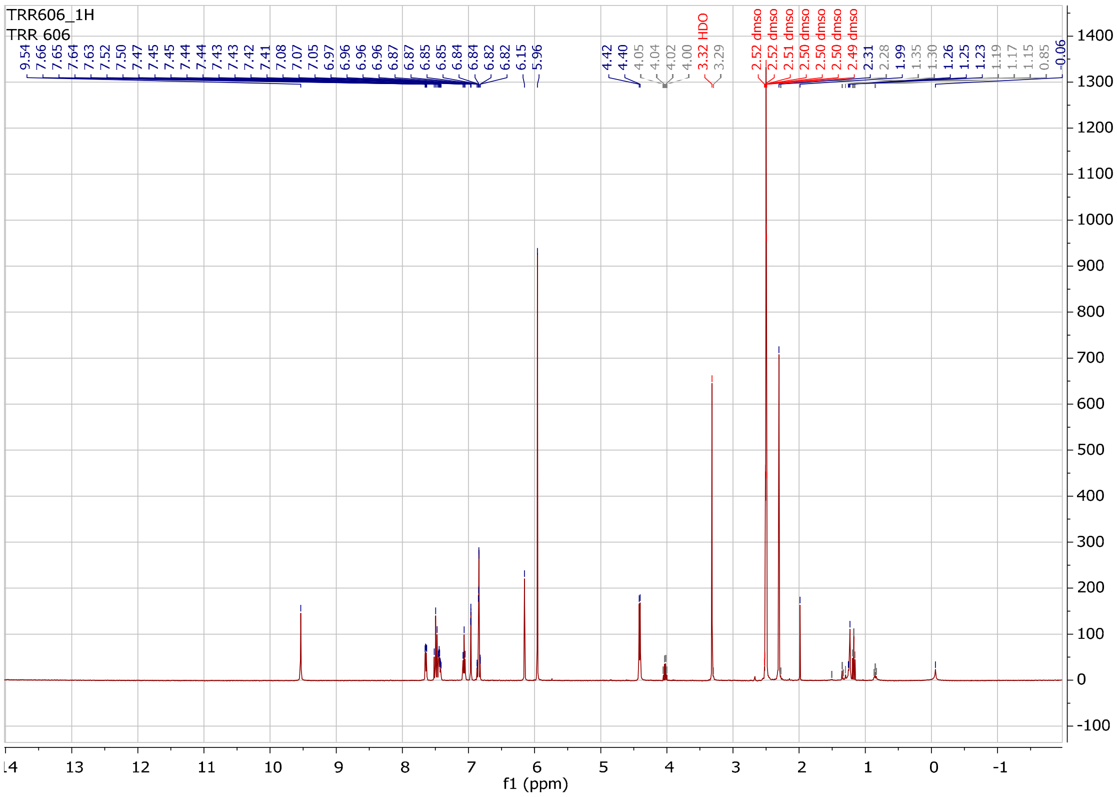

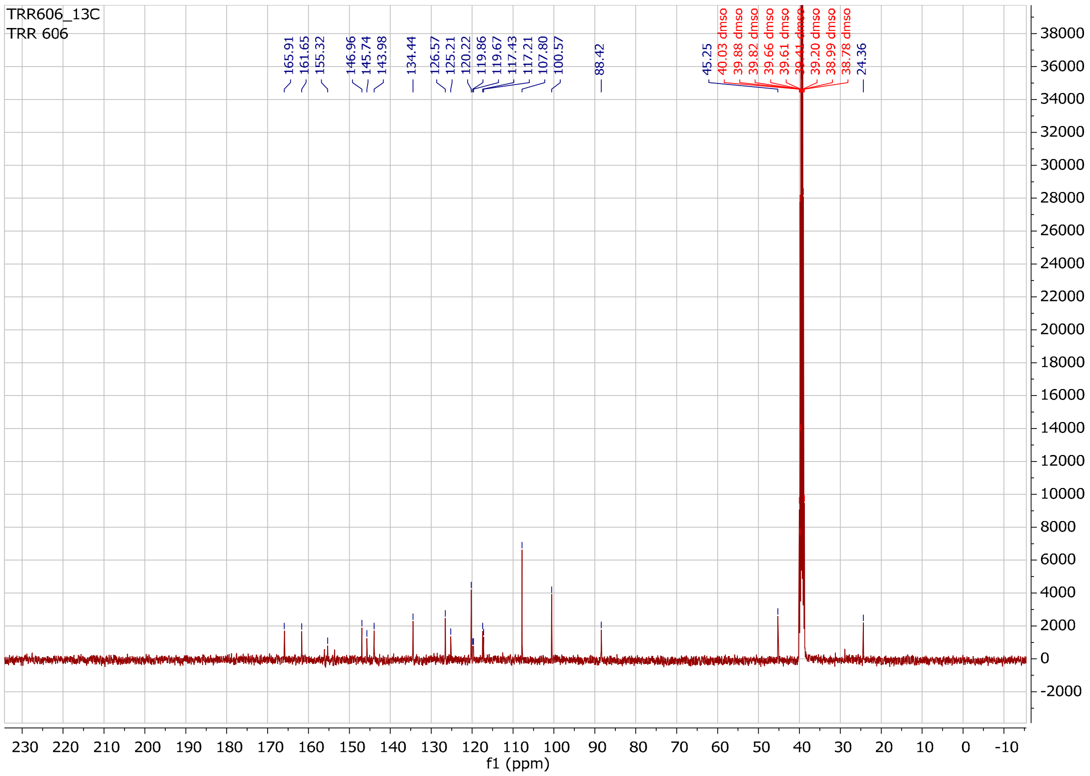

^{1}H-NMR and ^{13}C-NMR spectra of compound **8f**

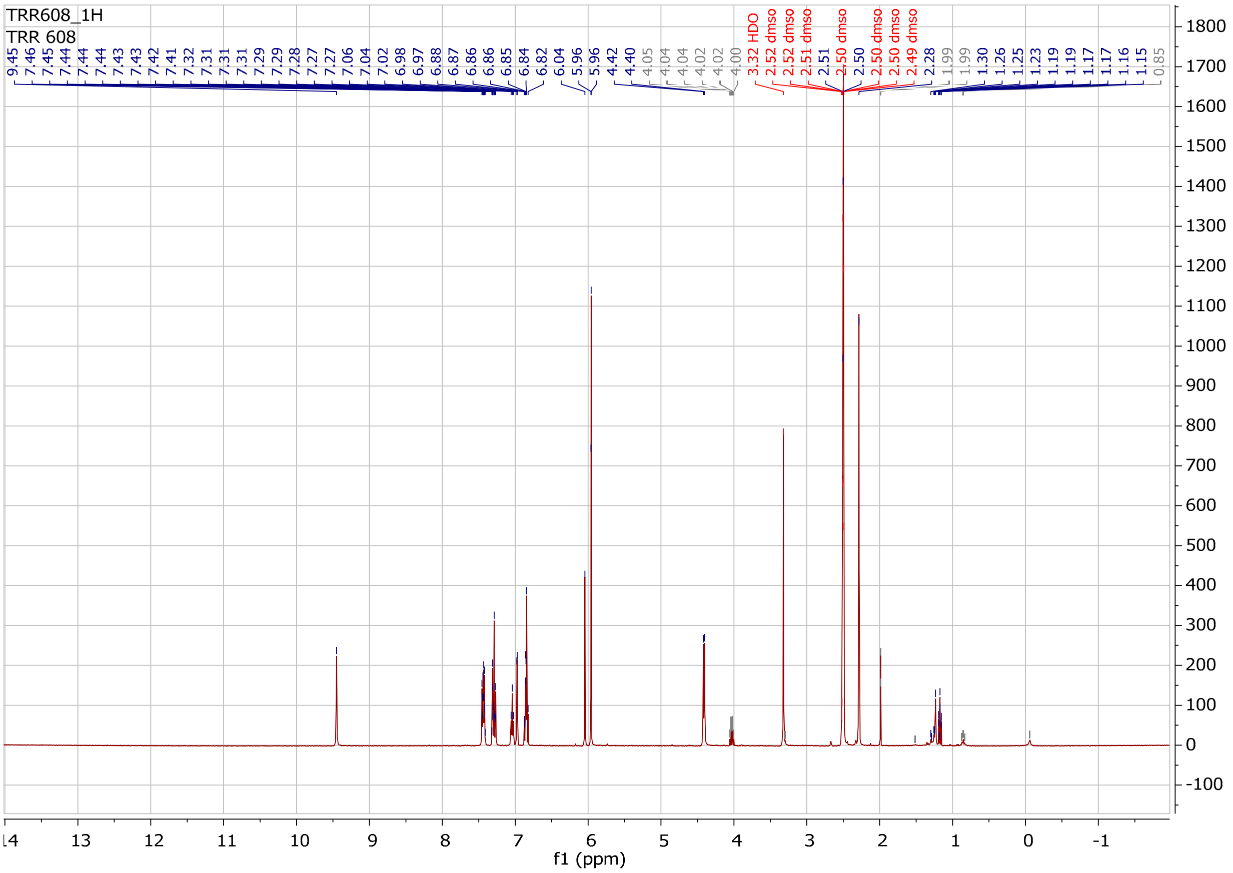

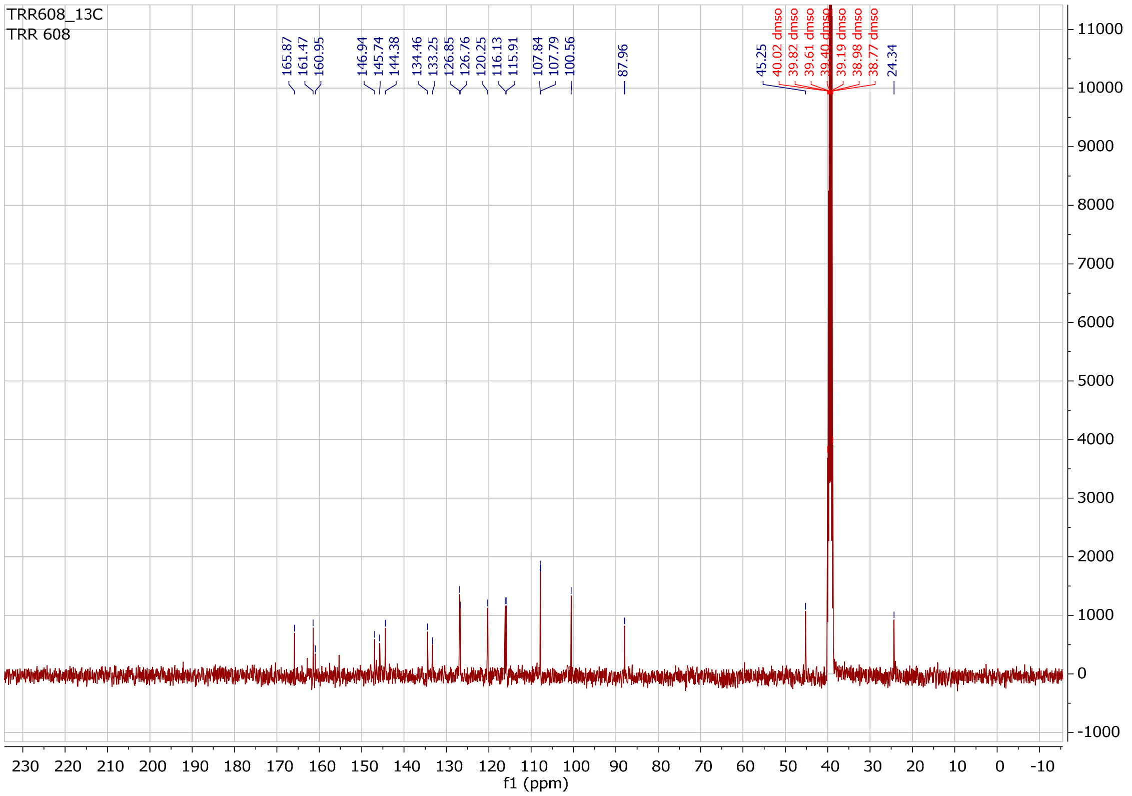

^{1}H-NMR and ^{13}C-NMR spectra of compound **8g**

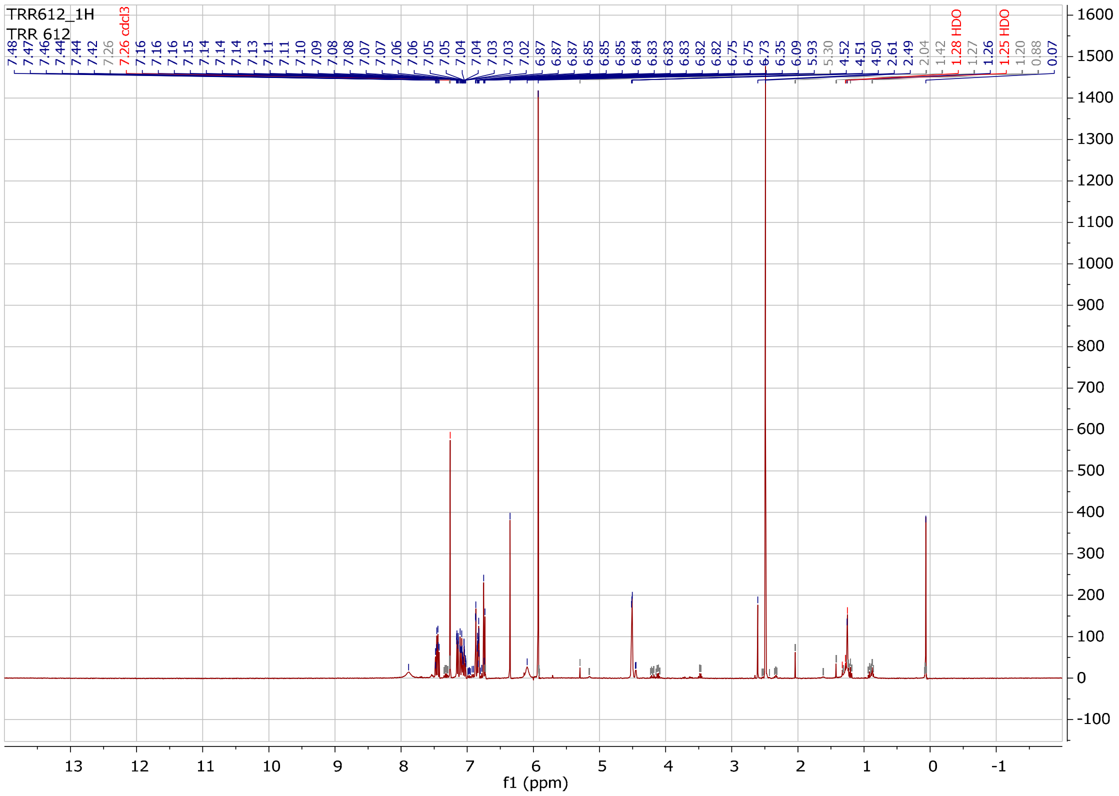

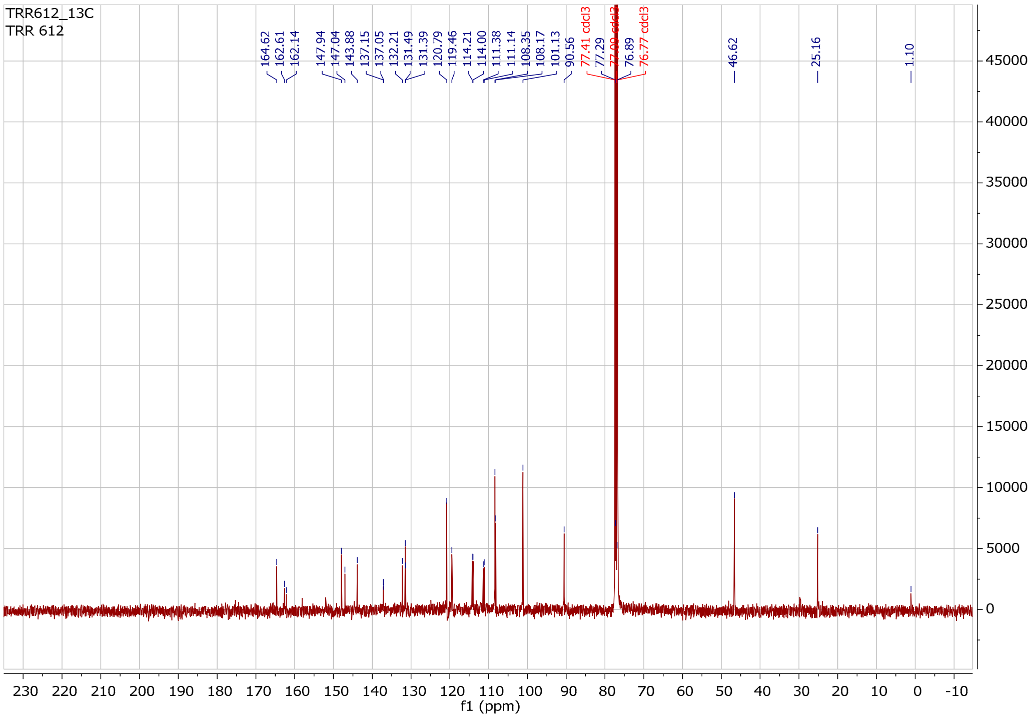

^{1}H-NMR and ^{13}C-NMR spectra of compound **8h**

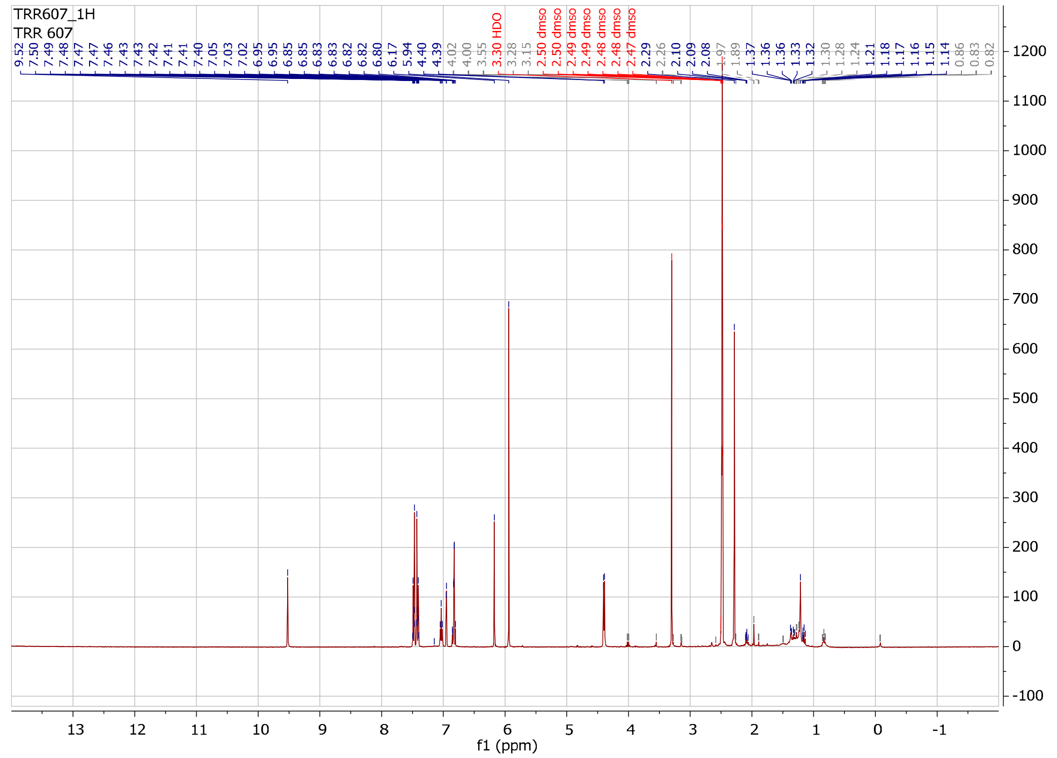

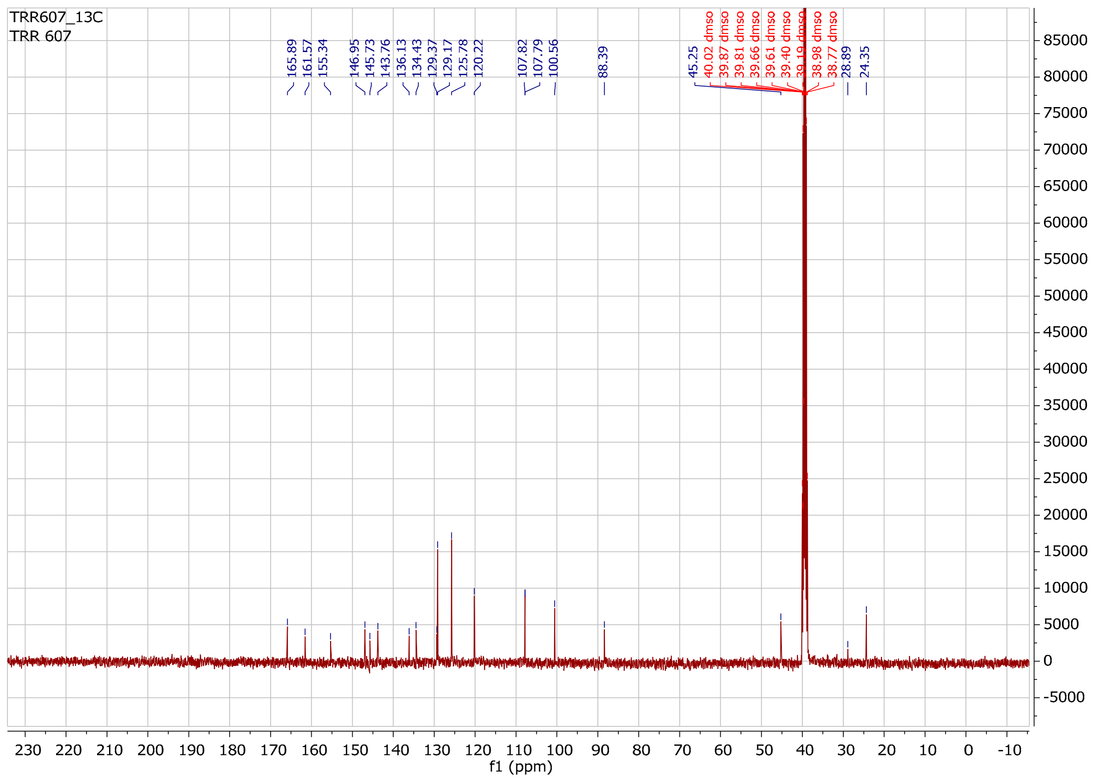

^{1}H-NMR and ^{13}C-NMR spectra of compound **8i**

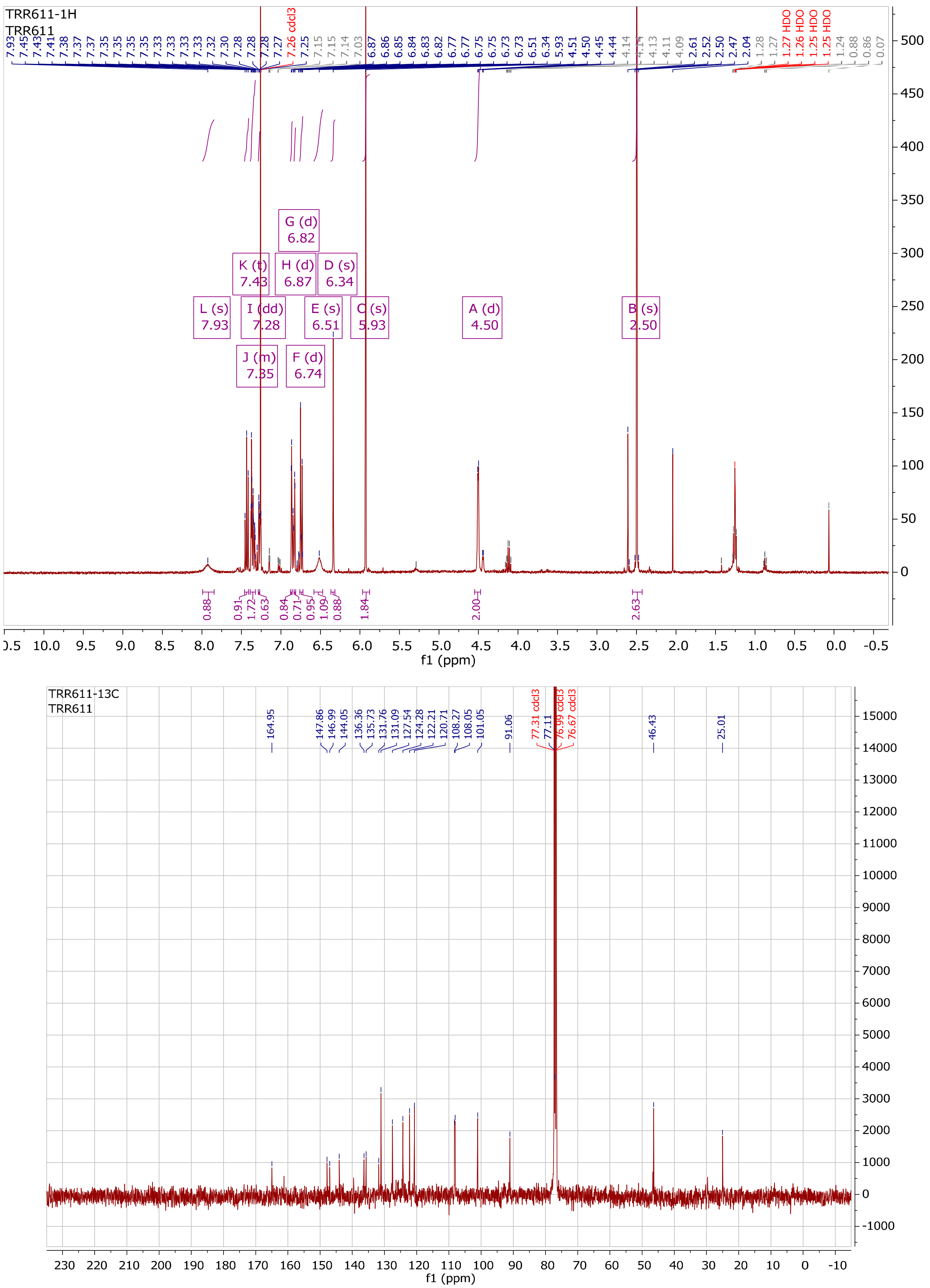

^{1}H-NMR and ^{13}C-NMR spectra of compound **8j**

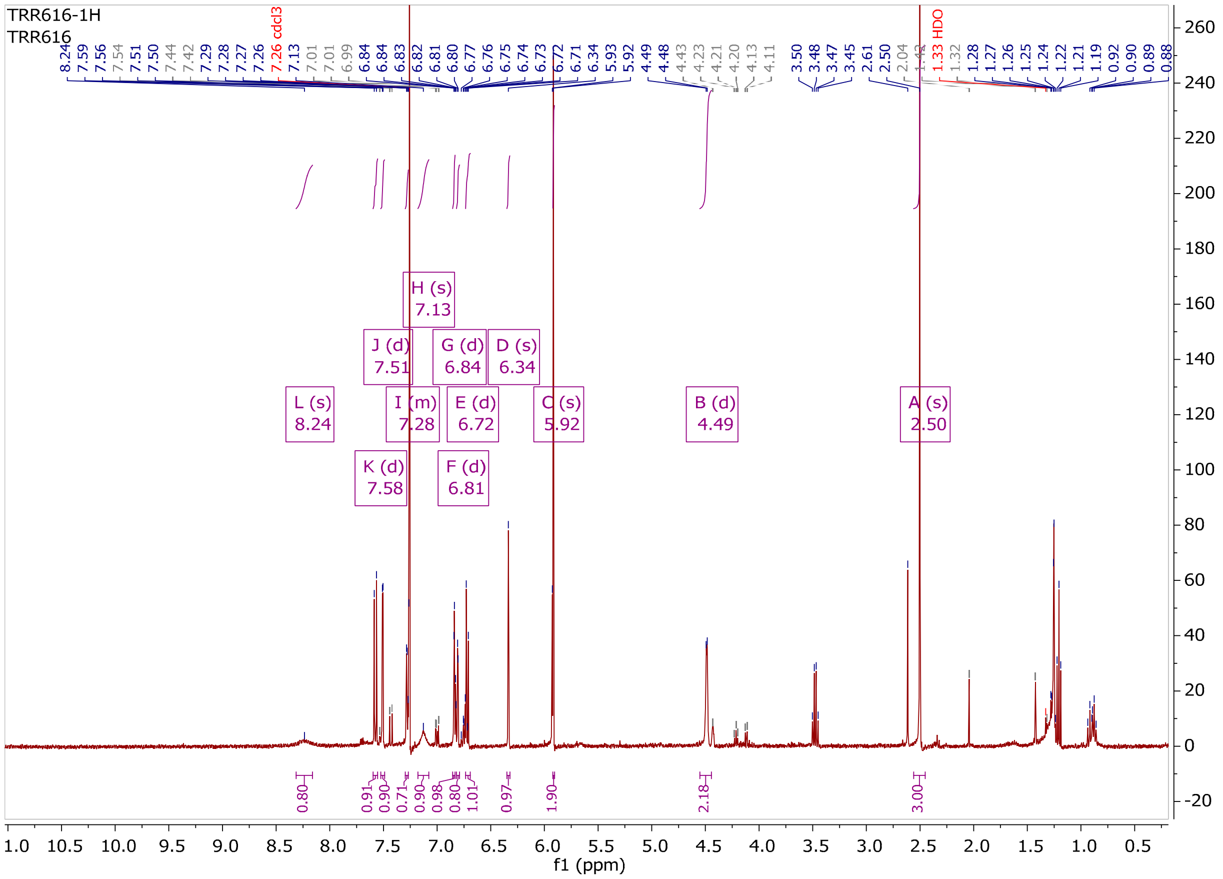

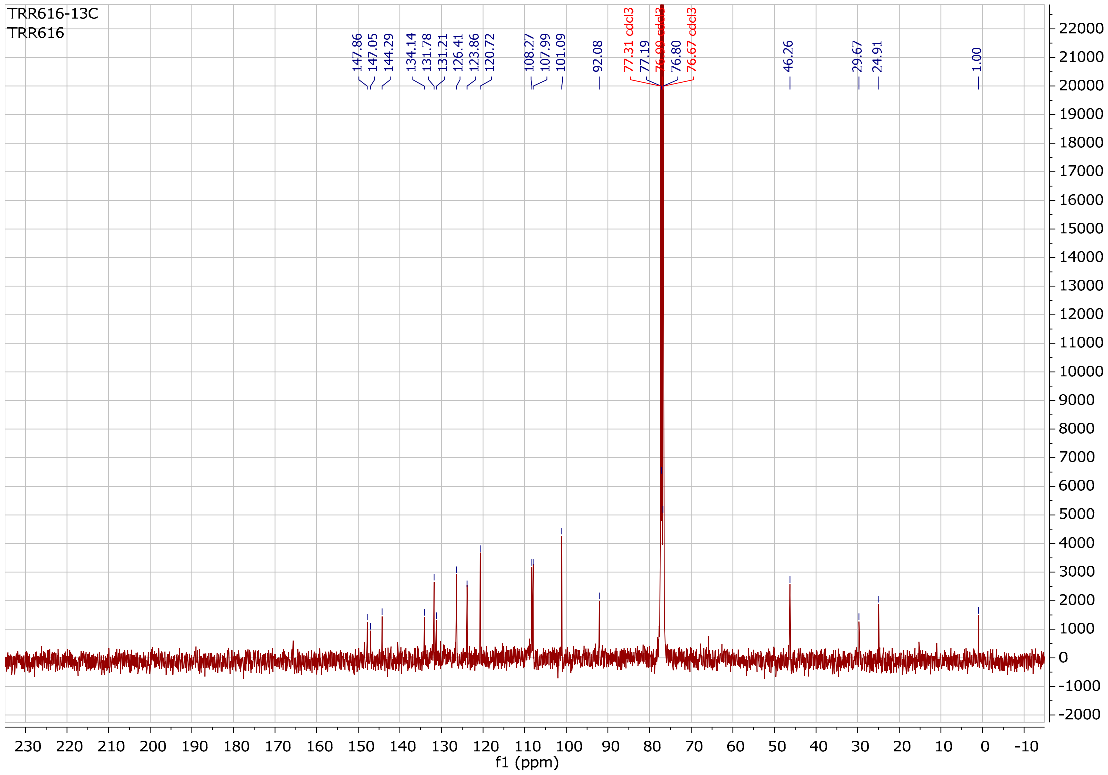

^{1}H-NMR and ^{13}C-NMR spectra of compound **8k**

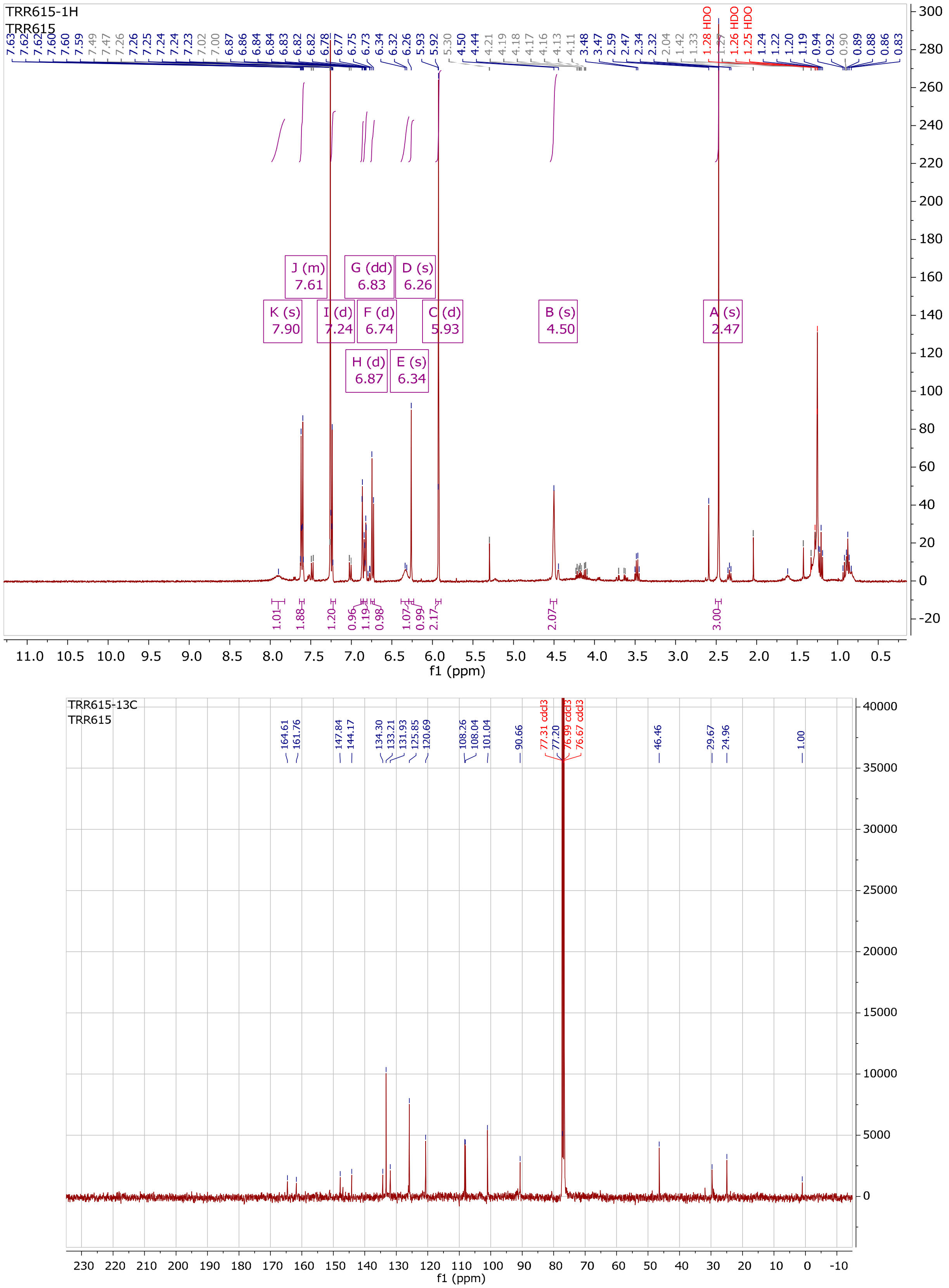

^{1}H-NMR and ^{13}C-NMR spectra of compound **8l**

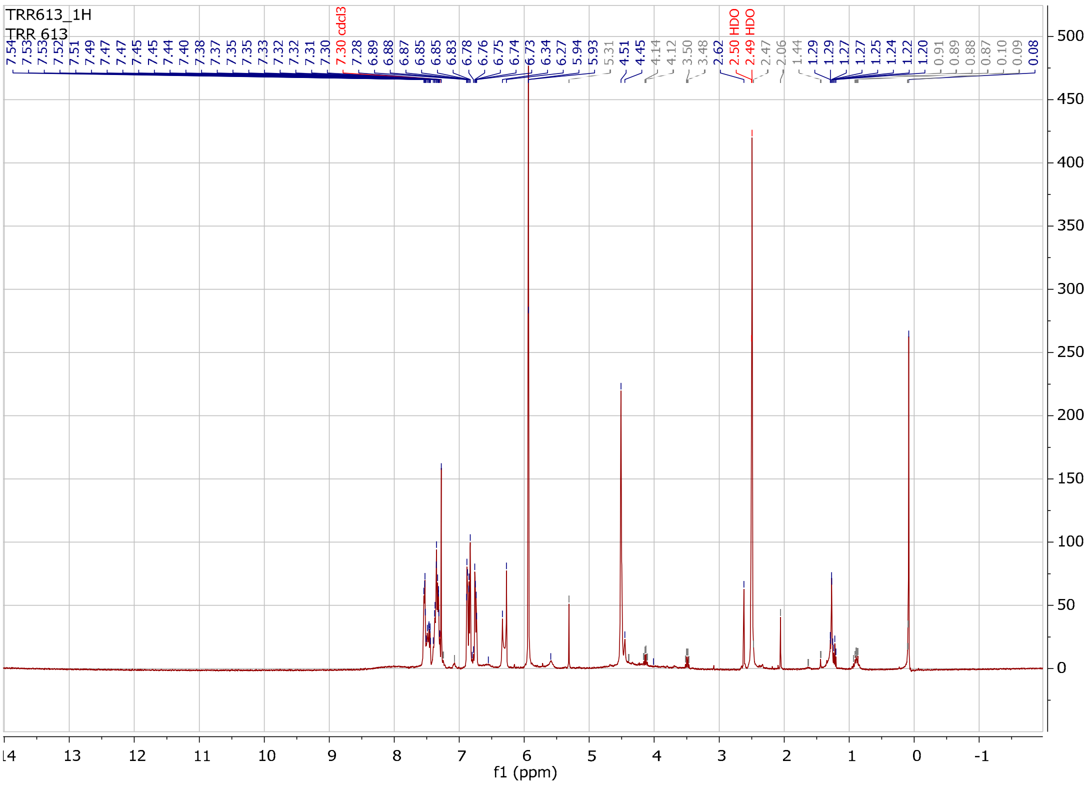

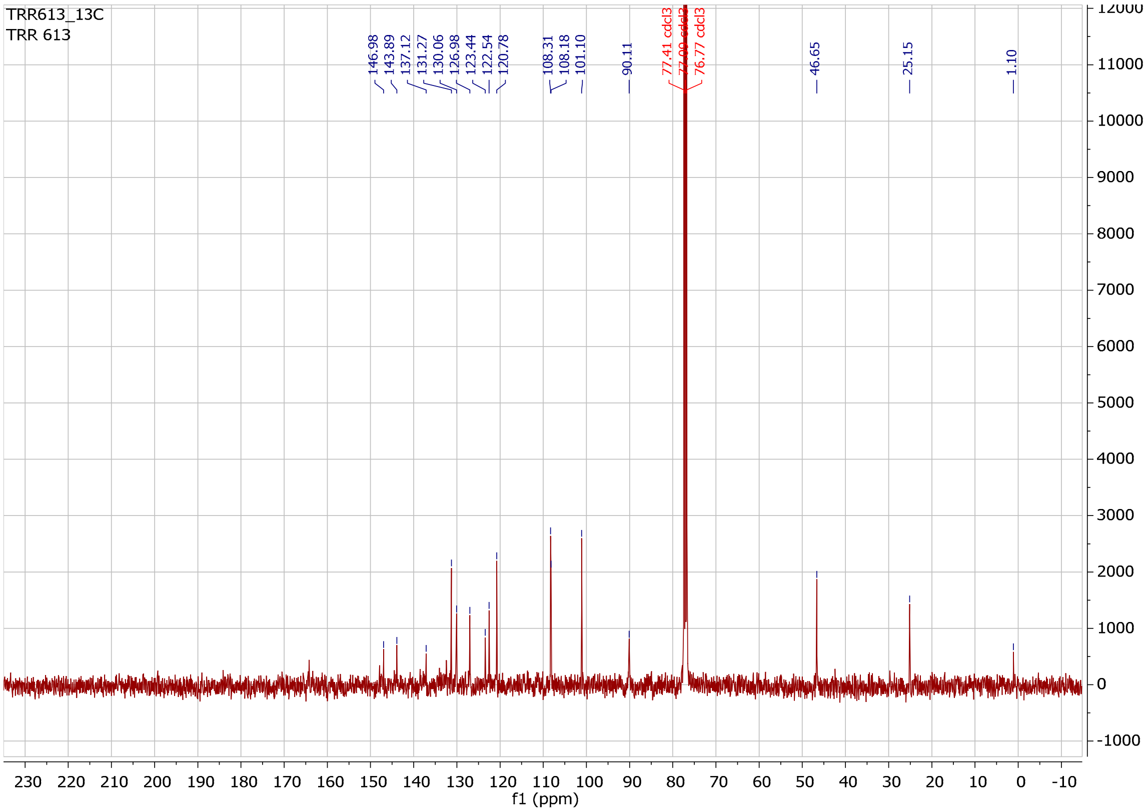

^{1}H-NMR and ^{13}C-NMR spectra of compound **8m**

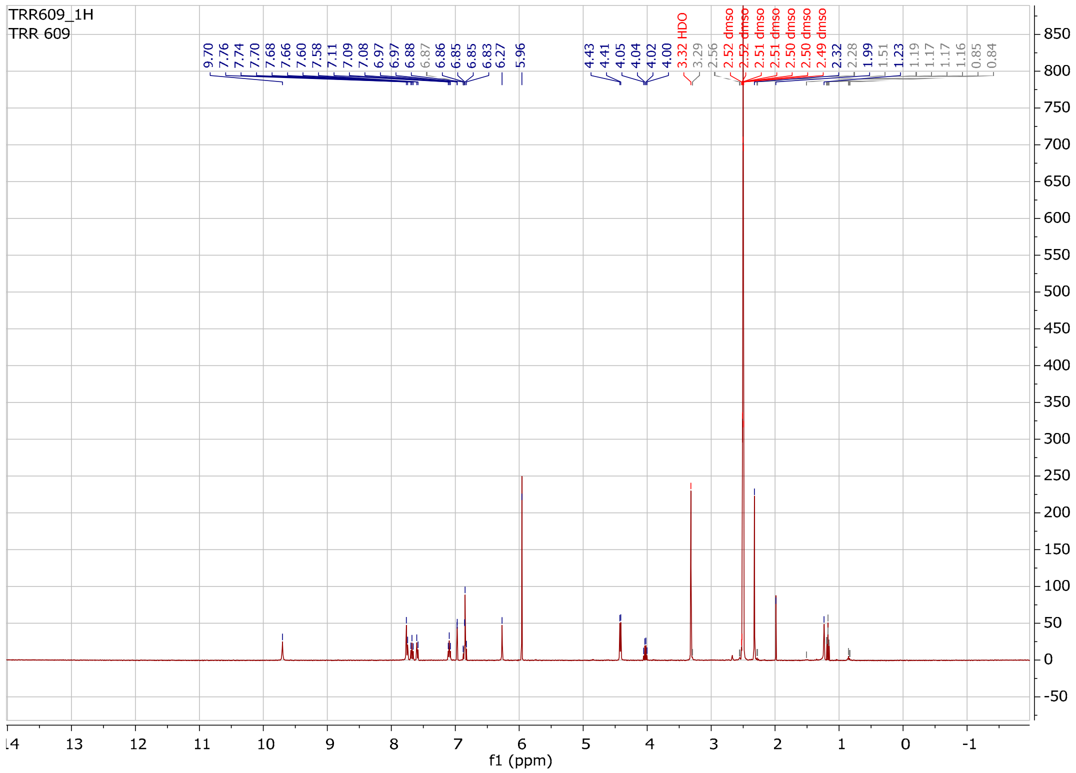

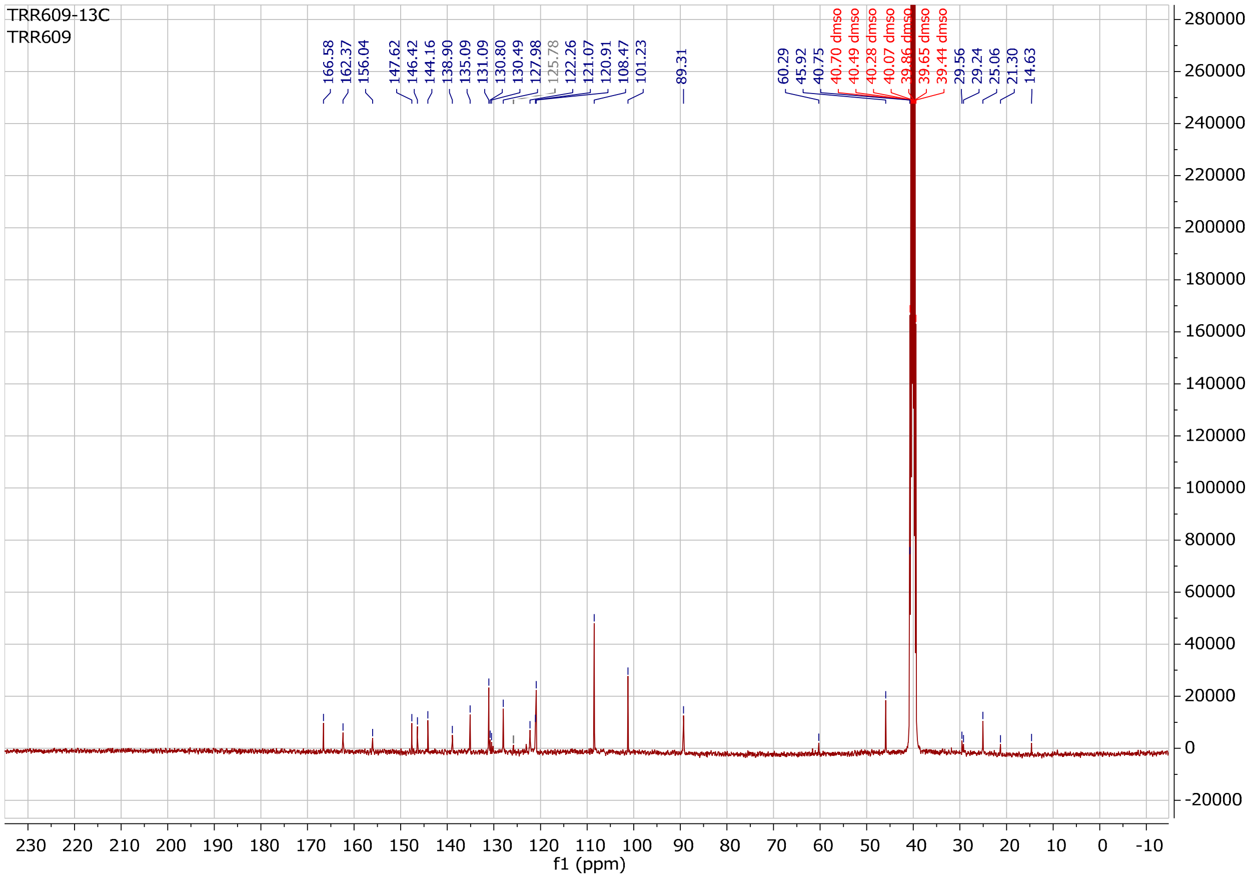

^{1}H-NMR and ^{13}C-NMR spectra of compound **8o**

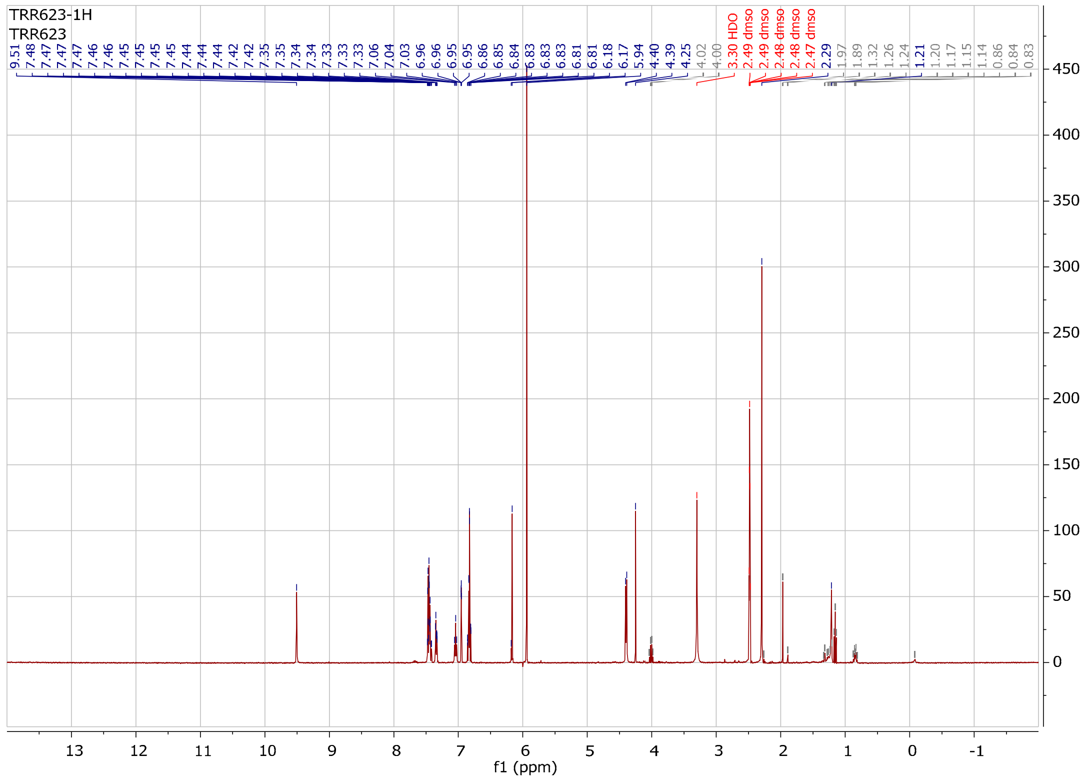

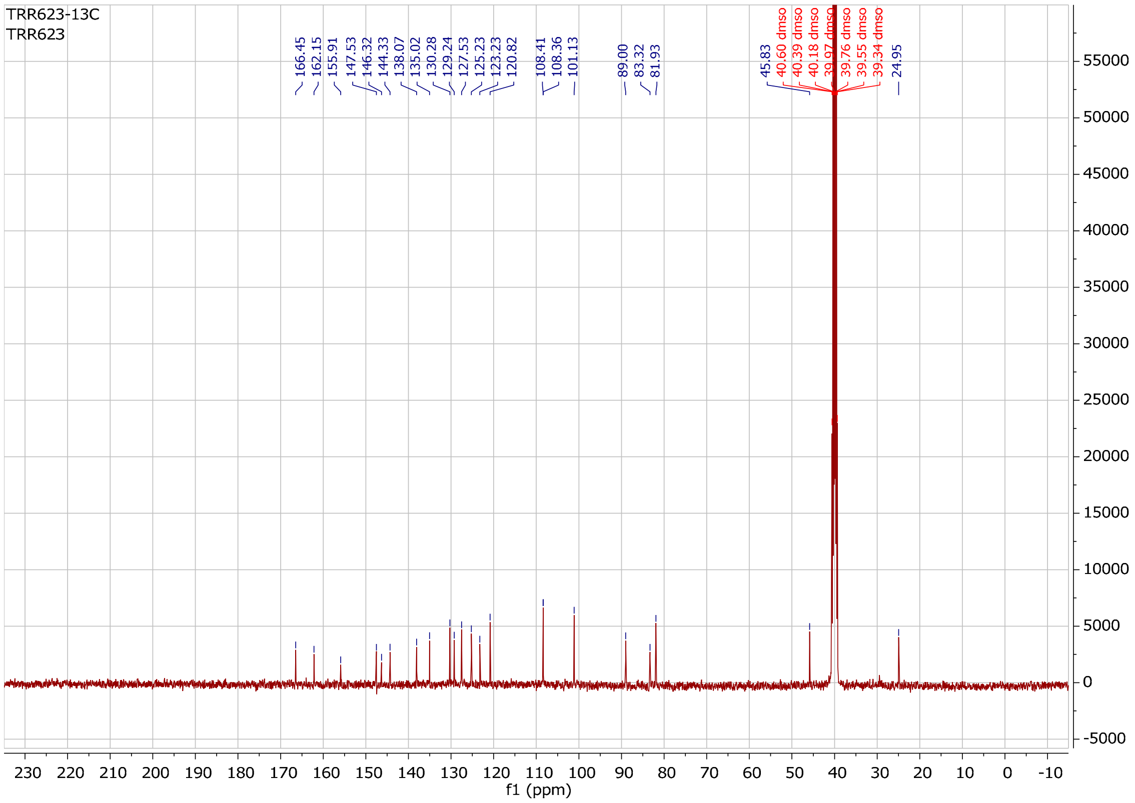

^{1}H-NMR and ^{13}C-NMR spectra of compound **8p**

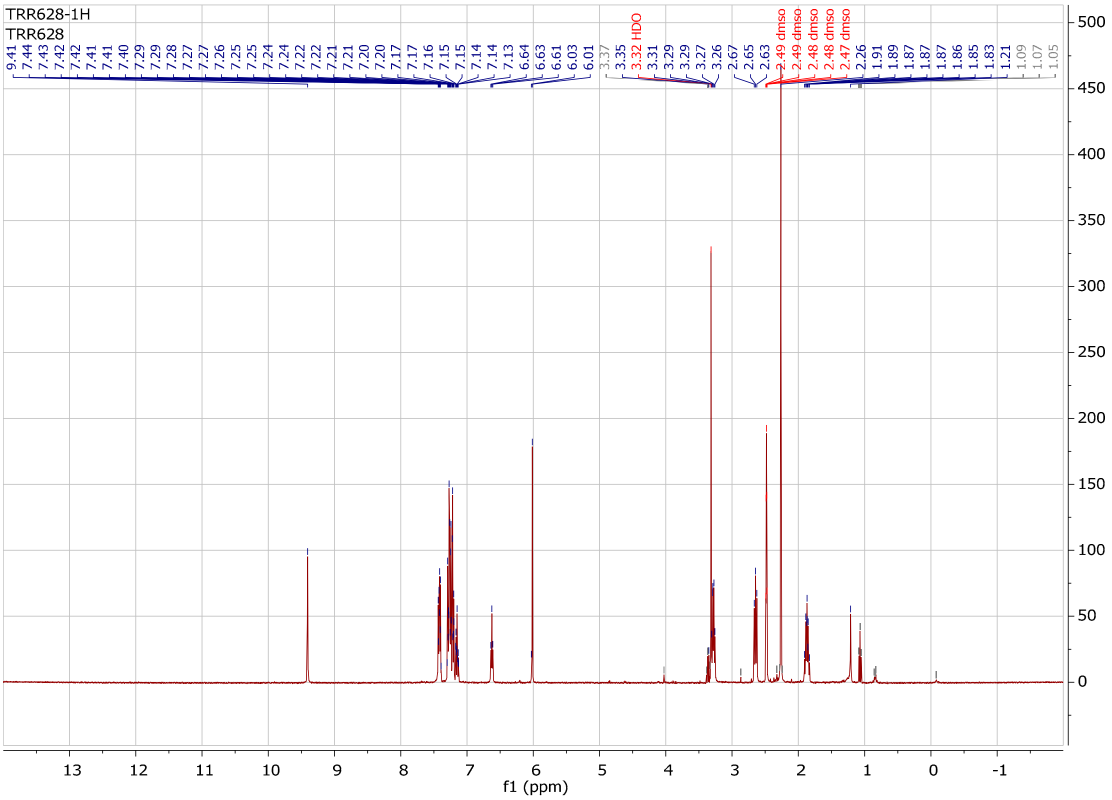

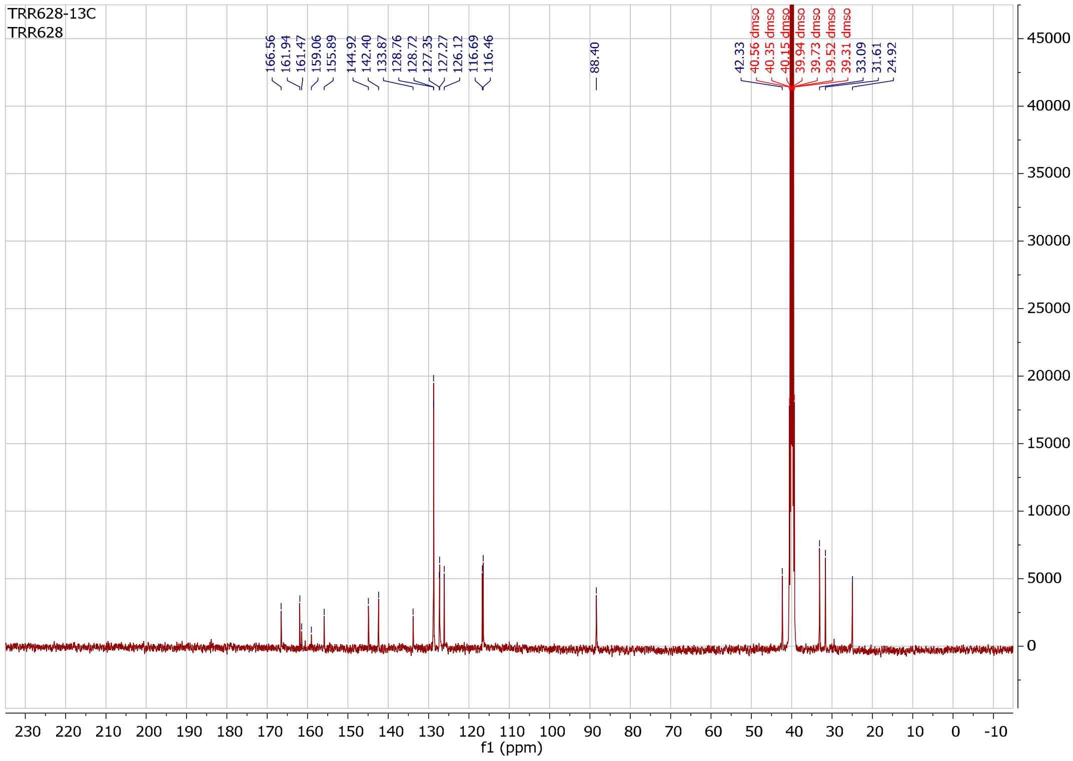

^{1}H-NMR and ^{13}C-NMR spectra of compound **8q**

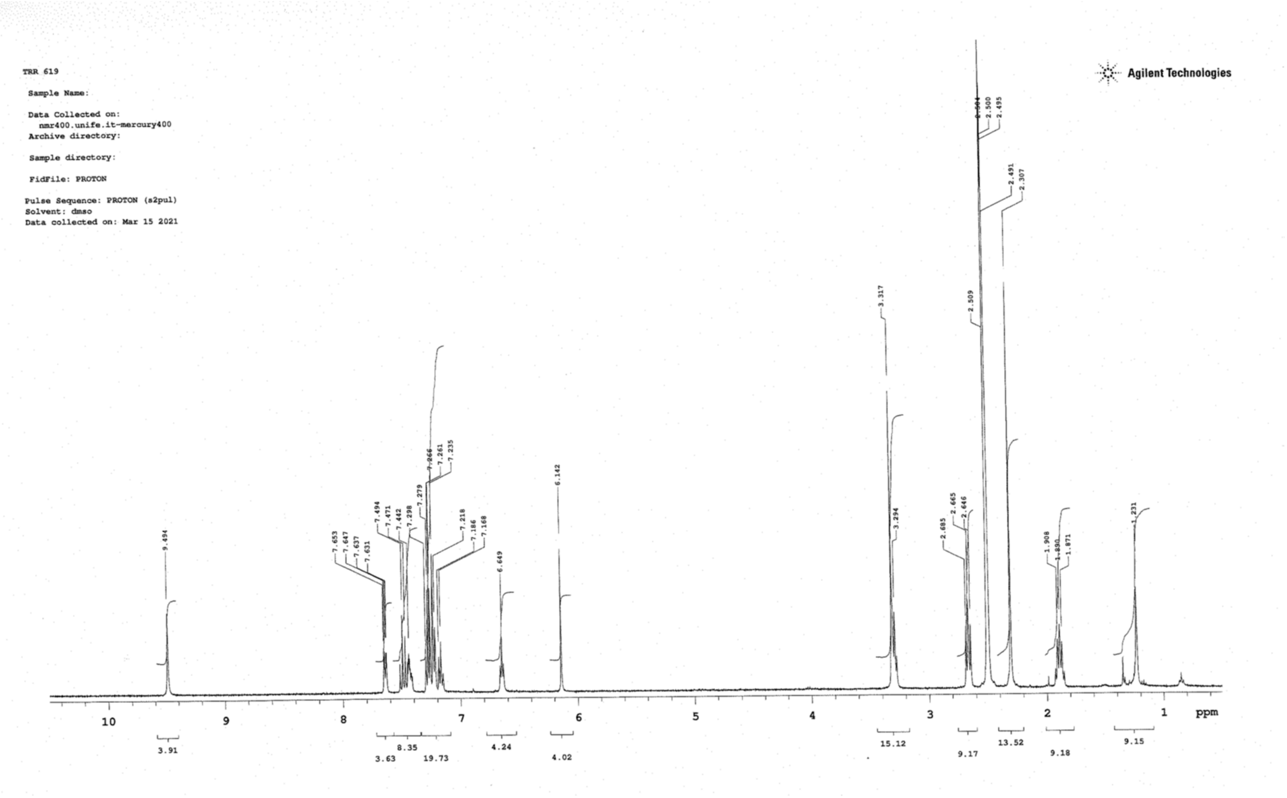

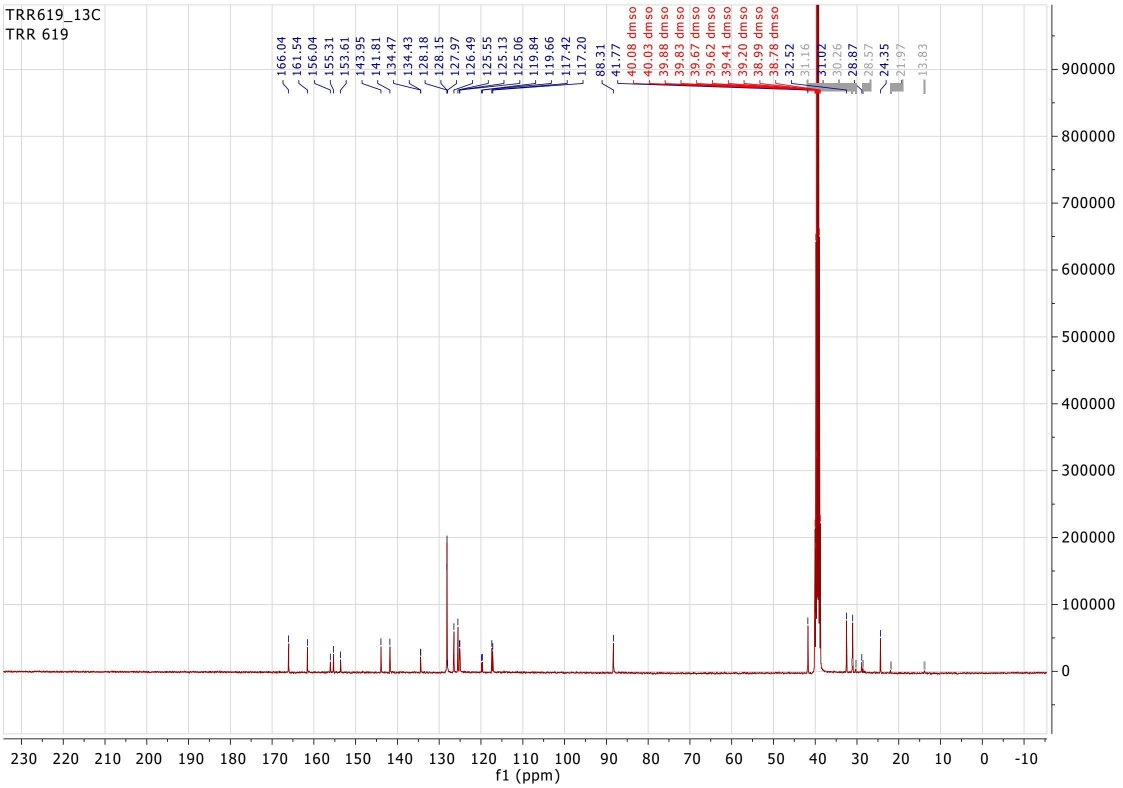

^{1}H-NMR and ^{13}C-NMR spectra of compound **8r**

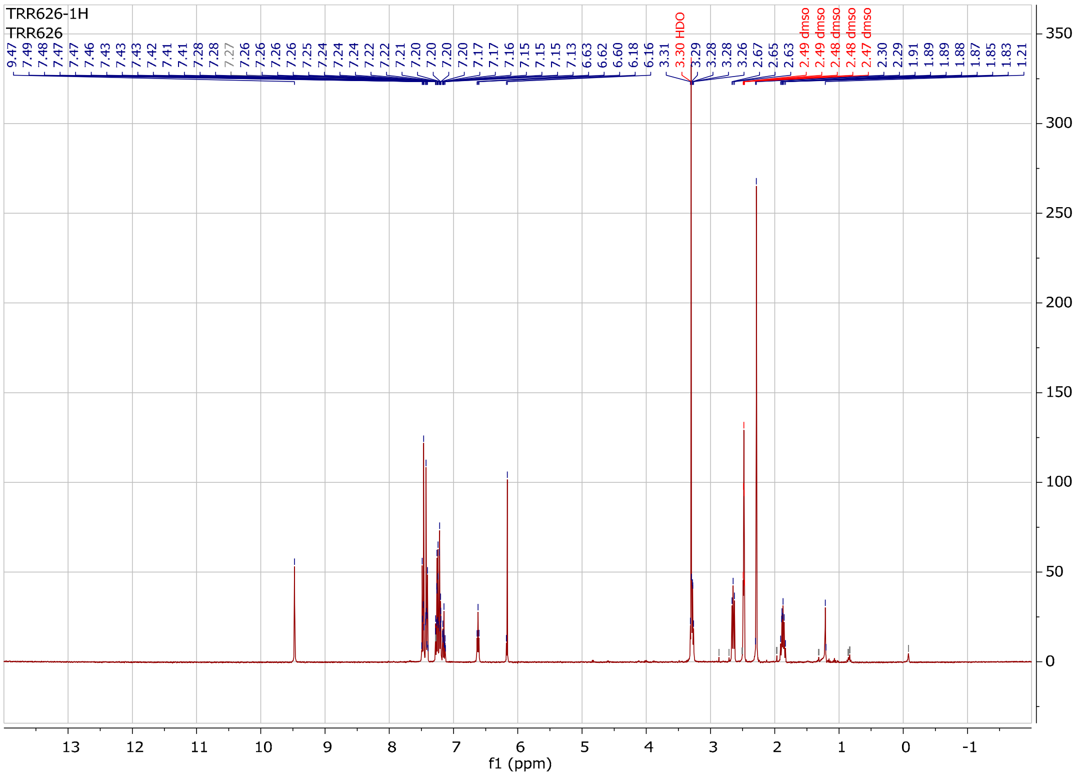

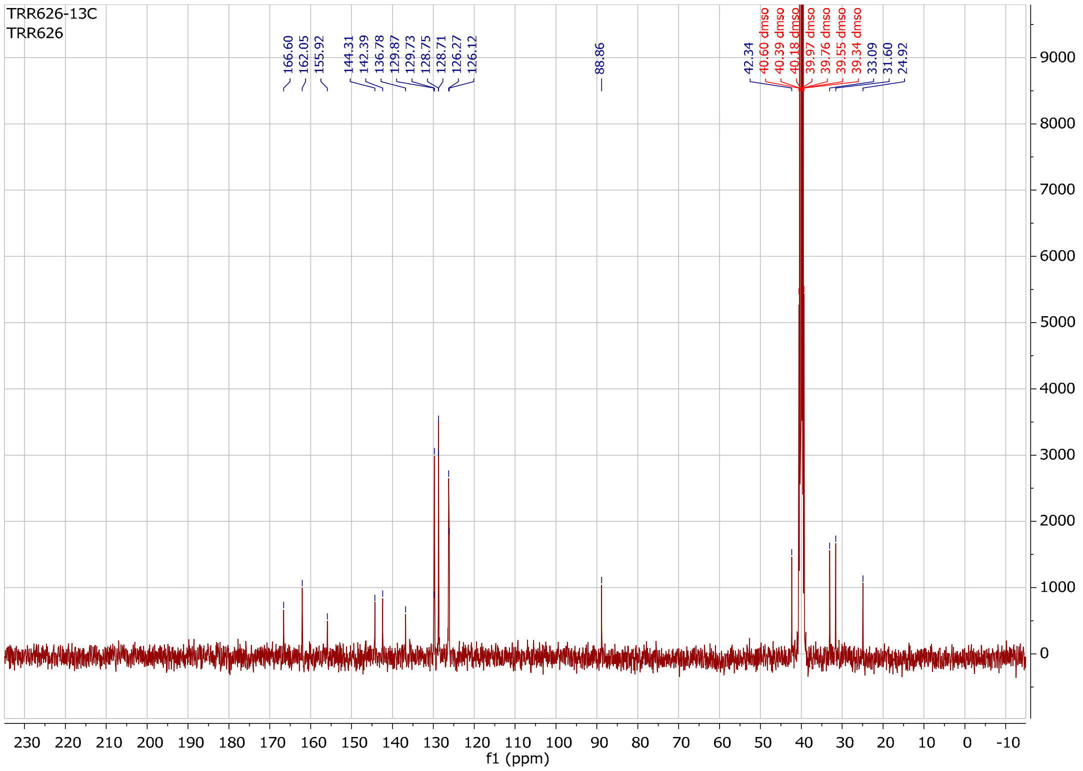

^{1}H-NMR and ^{13}C-NMR spectra of compound **8s**

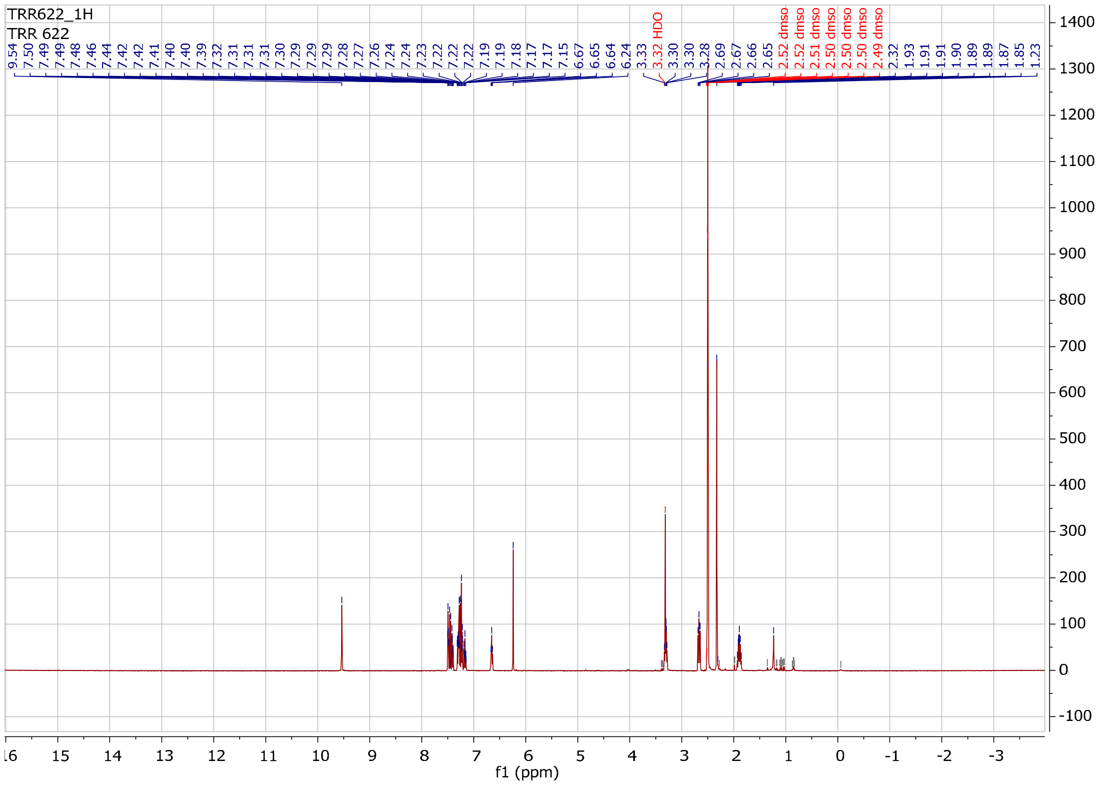

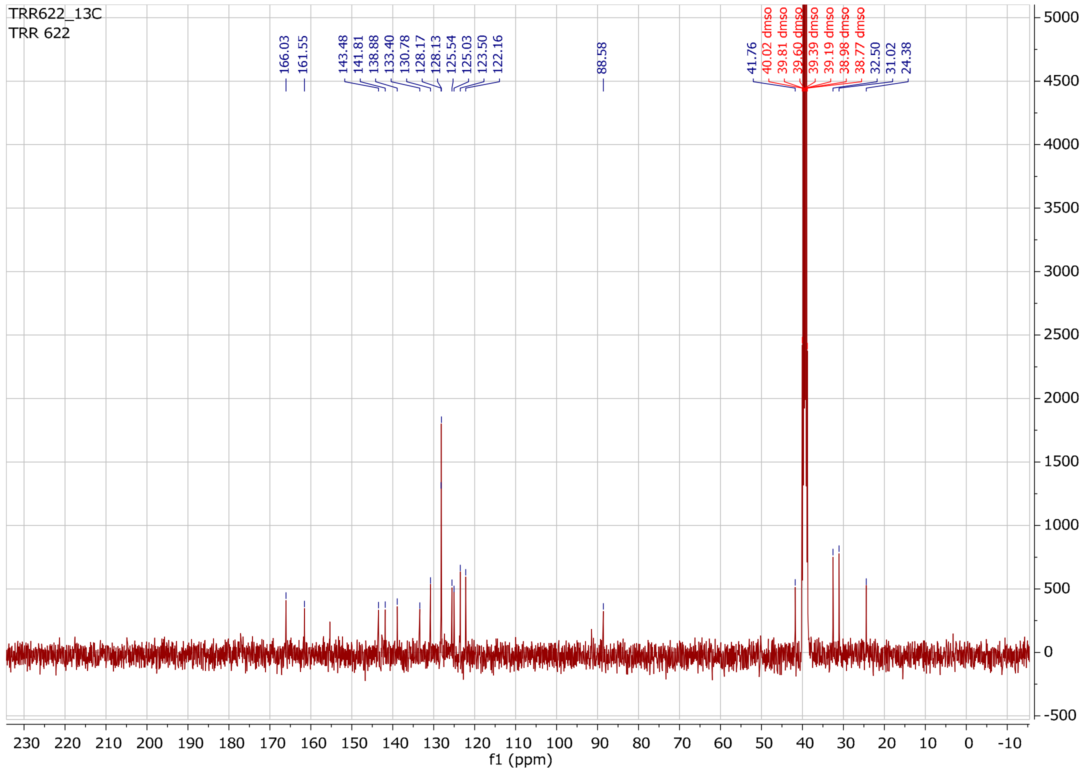

^{1}H-NMR and ^{13}C-NMR spectra of compound **8t**

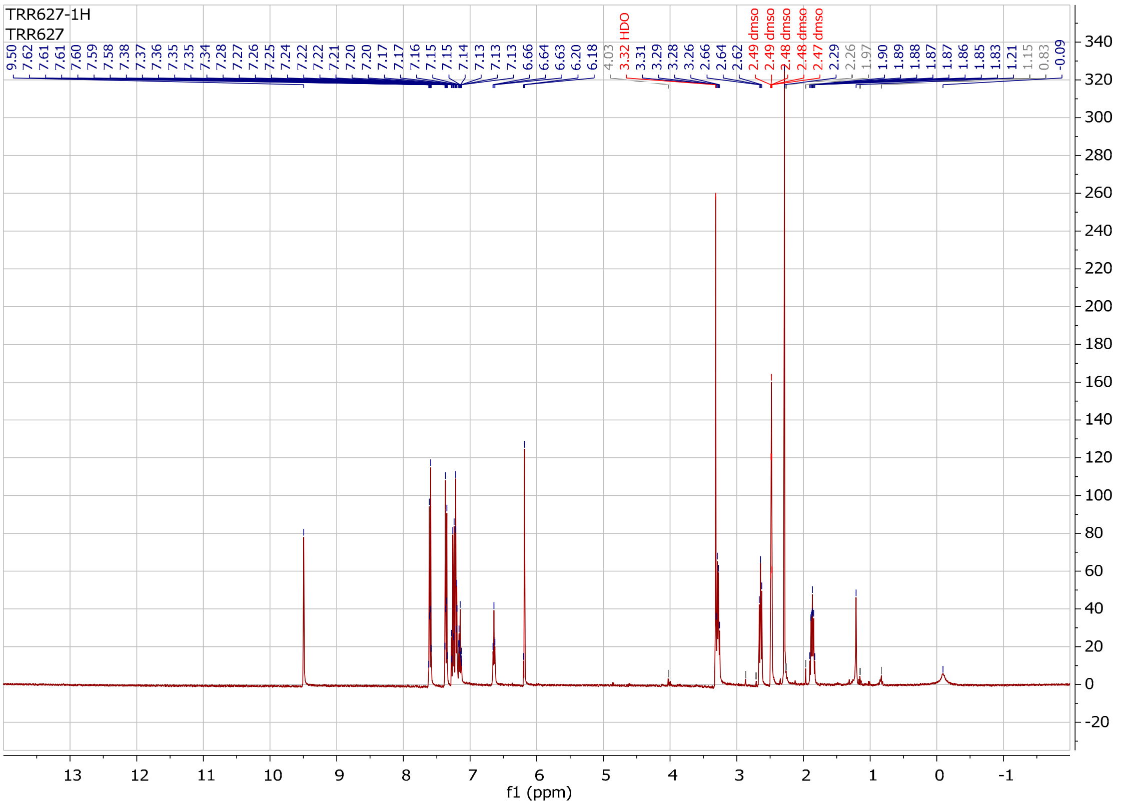

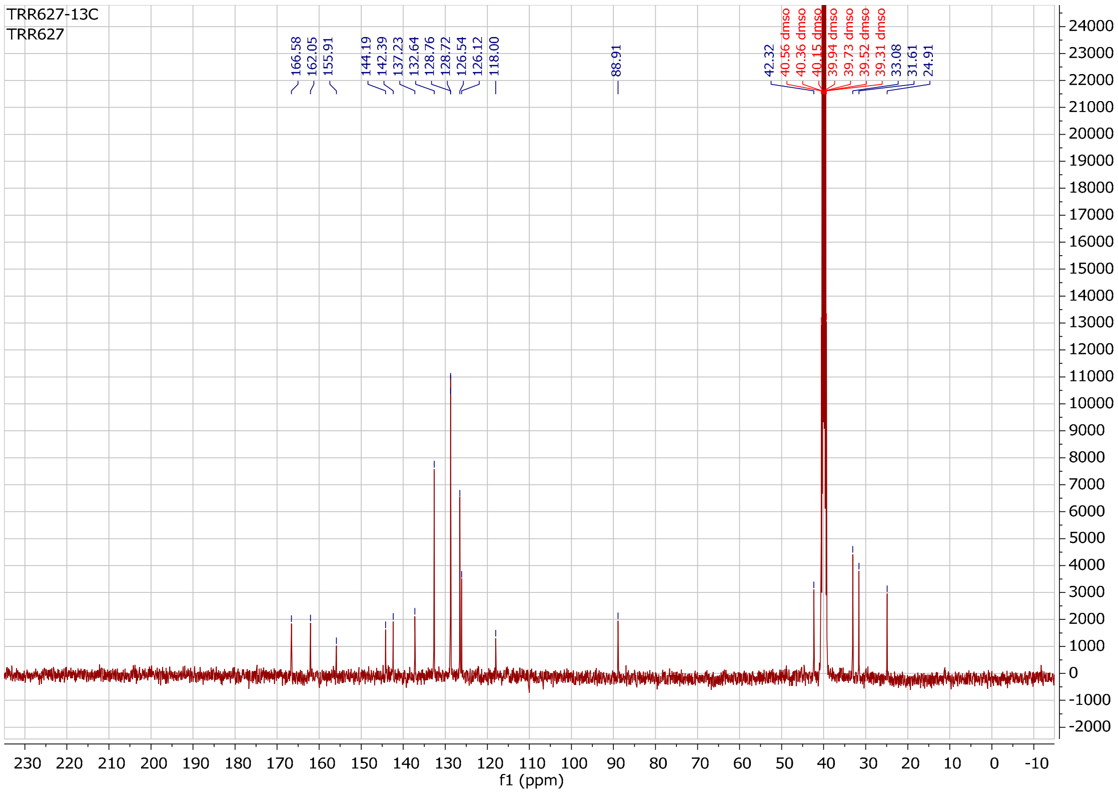

^{1}H-NMR and ^{13}C-NMR spectra of compound **8u**

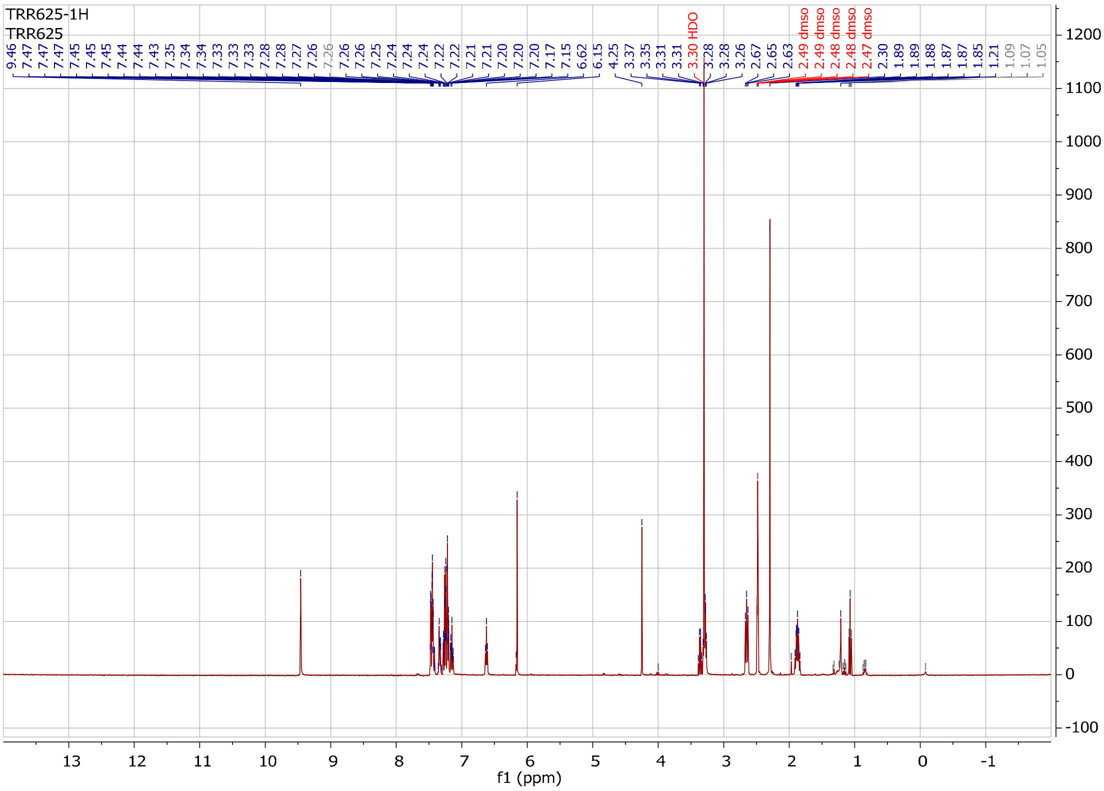

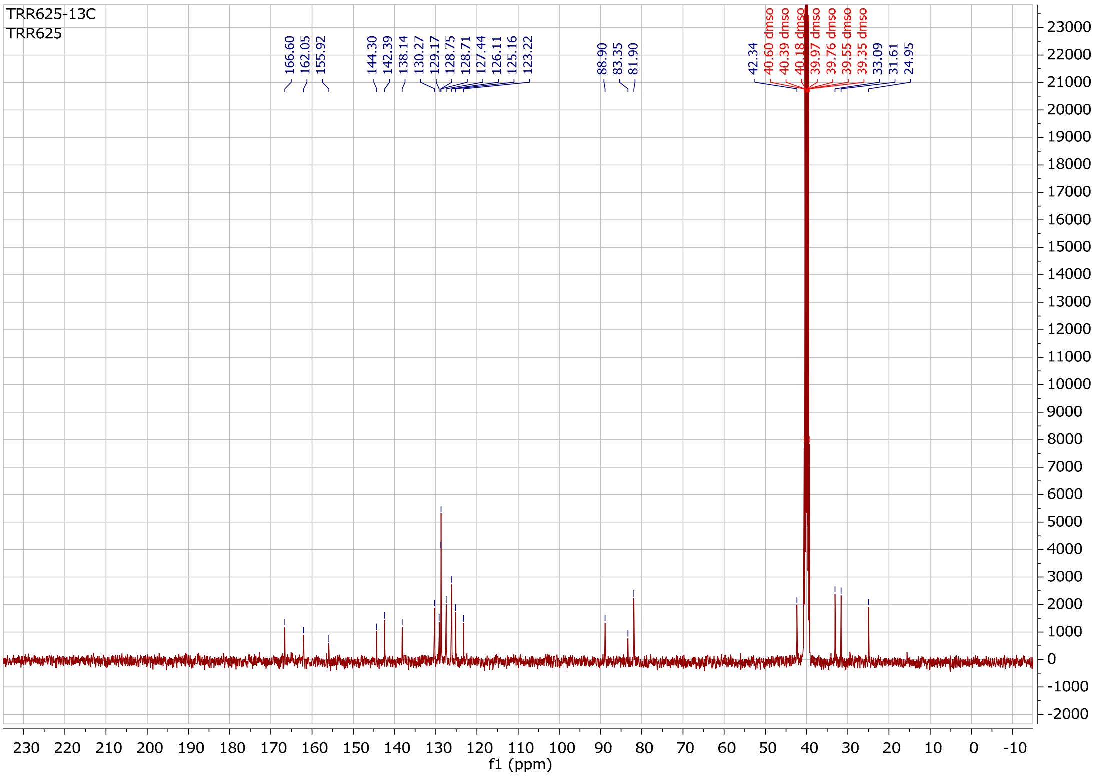

^{1}H-NMR and ^{13}C-NMR spectra of compound **8v**
